# Comparative transcriptome analysis of dikaryotic mycelia and mature fruiting bodies in the edible mushroom *Lentinula edodes*

**DOI:** 10.1038/s41598-018-27318-z

**Published:** 2018-06-12

**Authors:** Ha-Yeon Song, Dae-Hyuk Kim, Jung-Mi Kim

**Affiliations:** 1 Department of Bio-Environmental Chemistry, Institute of Life Science and Natural Resources, Wonkwang University, Iksan, Chonbuk 54538 Korea; 2 Institute for Molecular Biology and Genetics, Center for Fungal Pathogenesis, Chonbuk National University, Jeonju, Chonbuk 54896 Korea

## Abstract

*Lentinula edodes* is a popular cultivated edible mushroom with high nutritional and medicinal value. To understand the regulation of gene expression in the dikaryotic mycelium and mature fruiting body in the commercially important Korean *L*. *edodes* strain, we first performed comparative transcriptomic analysis, using Illumina HiSeq platform. *De novo* assembly of these sequences revealed 11,675 representative transcripts in two different stages of *L*. *edodes*. A total of 9,092 unigenes were annotated and subjected to Gene Ontology, EuKaryotic Orthologous Groups, and Kyoto Encyclopedia of Genes and Genomes (KEGG) analyses. Gene expression analysis revealed that 2,080 genes were differentially expressed, with 1,503 and 577 upregulated in the mycelium and a mature fruiting body, respectively. Analysis of 18 KEGG categories indicated that fruiting body-specific transcripts were significantly enriched in ‘replication and repair’ and ‘transcription’ pathways, which are important for premeiotic replication, karyogamy, and meiosis during maturation. We also searched for fruiting body-specific proteins such as aspartic protease, gamma-glutamyl transpeptidase, and cyclohexanone monooxygenase, which are involved in fruiting body maturation and isolation of functional substances. These transcriptomes will be useful in elucidating the molecular mechanisms of mature fruiting body development and beneficial properties, and contribute to the characterization of novel genes in *L*. *edodes*.

## Introduction

Basidiomycetous fungus *Lentinula edodes*, the shiitake mushroom, is the second most popular edible and medicinal mushroom in terms of total global output and economic value in East Asia^1,2^. *L*. *edodes* is broadly cultivated and managed and is one of two commercially important mushrooms in Korea^3^. Among Korean *L*. *edodes* cultivars, the dikaryotic strain Sanjo 701 (SJ701) in particular is popular for sawdust-bag cultivation and has been important in the development of monokaryotic strains for mating and fruiting^4^ and antibacterial substances against different phytopathogenic bacteria^5^.

One of the higher basidiomycota mushrooms, *L*. *edodes*, contains many pharmaceutical compounds that exhibit antibacterial, antifungal, antitumor, antioxidant, and/or immune-enhancing properties^6–10^. In addition, enzymes with commercial potential have been explored in various mushrooms, such as *Pleurotus ostreatus*, *Flammulina velutipes*, and *Coprinopsis cinerea*, including *L*. *edodes*, for use in bioremediation, biofuels, and foods^11,12^. Lignocellulolytic enzymes, cellulase, and hemicellulase from basidiomycota mushrooms are characterized by their bioconversion and bioremediation abilities^11–14^. Some proteases have been purified from mushrooms and used in food production, e.g., in milk clotting and as food additives^6,15^.

Mushroom enzymes with various functional properties, including antibiotic, antioxidant, and antitumor activities, have been extracted from cell culture filtrates as well as mycelia and fruiting bodies^5^. Because *L*. *edodes* is an invaluable fungus in agriculture, medicine, and industry, it will be important for continual development of novel and superior *L*. *edodes* cultivars. However, the breeding of new mushroom strains using typical breeding methods is time consuming and laborious compared with the methods used for other mushrooms such as *F*. *velutipes* and *P*. *ostreatus*^16^. Hence, an effective genetic approach is essential for the development of new breeding methods^4,17^. In addition, gene expression of a target gene related to a specific beneficial property must be performed at different stages, e.g., mycelia, brown mycelial film, primordium, and the fruiting body^18,19^, to determine the stage at which optimum activity or the maximum yield of the desired substance occurs. Therefore, determining genetic changes compared with the parental strains at different developmental stages using high-throughput sequencing technology (next-generation sequencing technology)^20^ will be necessary to generate improved strains and varieties of mushrooms by genetic modification.

To analyze differentially expressed genes (DEGs) between two major developmental stages (i.e., mycelium and fruiting body), high-throughput sequencing has been used for genome and transcriptome analyses of basidiomycota mushrooms, including *Agrocybe aegerita*, *Auricularia polytricha*, *Cordyceps militaris*, and *Ganoderma lucidum*. Fruiting body formation is regulated at the transcriptional level and results in morphological changes during development and stage-specific expression to produce beneficial properties^21–24^. To date, there have been no transcription-level, genome-wide comparative studies of the development stages of mycelia and mature fruiting bodies of *L*. *edodes* commercial strains and their relation to yield. Two monokaryotic strains of *L*. *edodes* have been sequenced and annotated by high-throughput sequencing^25,26^ and extensive expressed gene sequence data of a Chinese strain was obtained by deep Solexa sequencing^27^. Differences in expression between the dikaryotic mycelium and primordium have also been investigated by serial analysis of gene expression^28^. Comparative transcriptome analysis has been performed to assess fruiting bodies at different stages of development^19,29^. In addition, transcriptome analyses have been conducted in light-induced brown film, different growth stages of the fruiting body, and a post-harvested fruiting body^16,17,19^, and post genome-wide studies have been performed to understand the complex molecular mechanisms underlying gene regulation in *L*. *edodes*. However, despite the great importance of investigating the specific stages for the optimal production of beneficial nutritional and medicinal properties and the generation of improved *L*. *edodes* cultivars, the transcriptomes of dikaryotic mycelia and mature fruiting bodies of this species remain poorly understood. In particular, there have been no reports of comparative transcriptomic analysis of a Korean *L*. *edodes* cultivar using the NGS technique, despite active culturing and consumption of great quantities of edible *L*. *edodes* mushrooms in other East Asian countries. In this study, we performed the first comparative transcriptome analysis of two developmental stages, the mycelium and mature fruiting body.

In this study, we performed the first comparative transcriptome analysis of two developmental stages, the mycelium and a mature fruiting body, of *L*. *edodes* using Illumina sequencing technology to explore the transcriptome during maturation of the fruiting body at the genome level. Approximately 30 million reads were obtained from each sample and mapped to the *L*. *edodes* genome. A total of 11,675 unigenes were identified by *de novo* assembly, and analyses of the DEGs revealed genes involved in fruiting body maturation of *L*. *edodes*. A functionally annotated and classified transcriptome dataset will provide valuable genomic information for further studies in *L*. *edodes*. The identification of differentially expressed genes (DEGs) involved in mycelium and fruiting body development will improve our understanding of the maturation and stage-specific expression of functional properties of this edible mushroom, and provide valuable genetic information for further studies of the molecular mechanism aiming to improve productivity.

## Results and Discussion

### Transcriptome sequencing of *L*. *edodes* and *de novo* assembly

**Figure 1:**
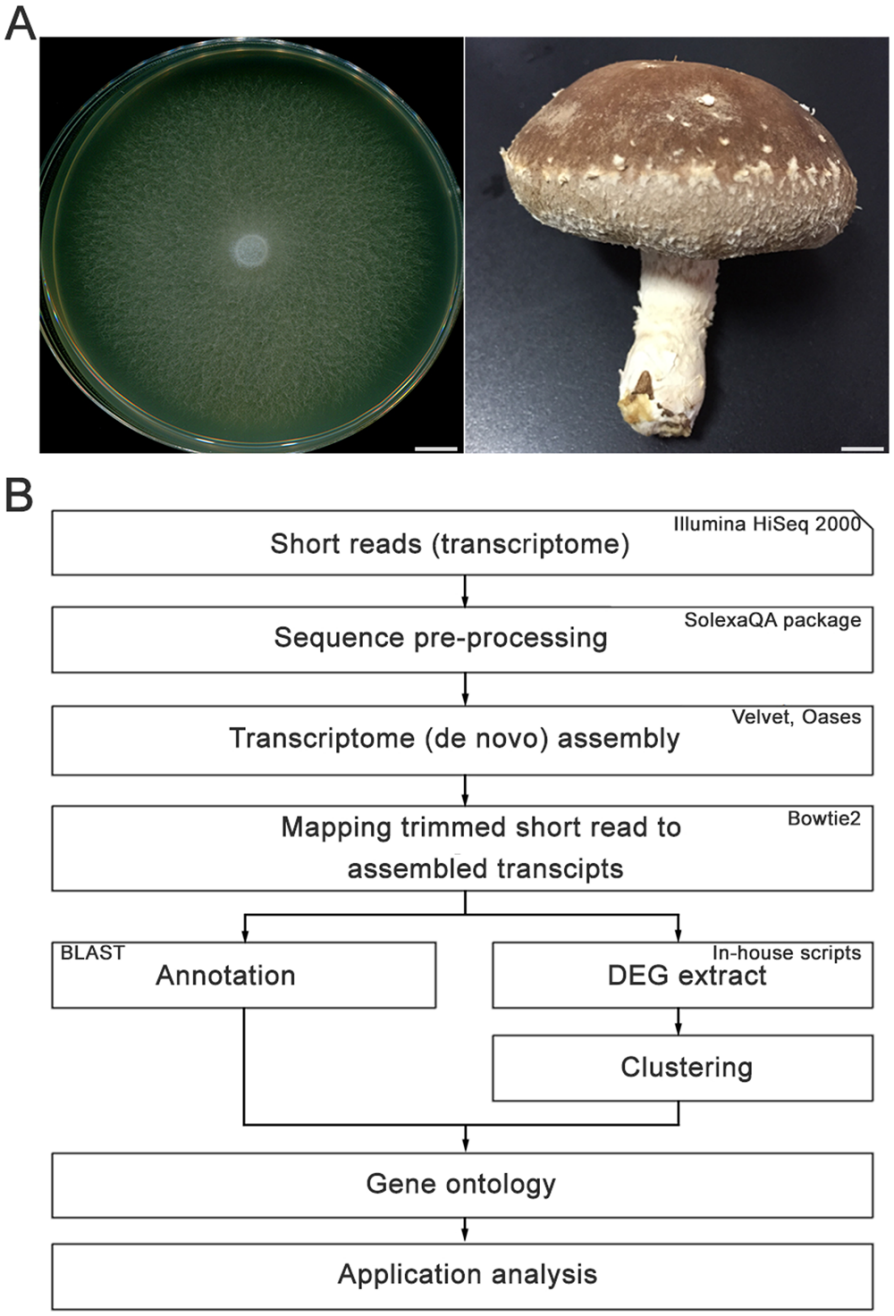
Schematic of the comparative transcriptome analysis in *Lentinula edodes*. (**A**) Representative images of different developmental stages: vegetative mycelium (left) and mature fruiting body of *L*. *edodes* (right). Scale bar = 1 cm. (**B**) Overall workflow of the transcriptome assembly and analysis of the RNA sequencing (RNA-seq) data in *L*. *edodes*.

To identify transcriptomic changes in two developmental stages of the commercially important Korean *L*. *edodes* strain Sanjo701 (SJ701)^3,4^, high-throughput sequencing in RNA samples extracted from *L*. *edodes* dikaryotic mycelia and mature fruiting bodies was the first performed using Illumina paired-end sequencing technology according to the transcriptome analysis workflow (Fig. 1).

In total, 27,646,278 and 30,109,768 clean paired-end sequence reads were obtained from the mycelium and mature fruiting body, respectively, with a Q20 percentage >72.2%, using the DynamicTrim and LengthSort programs^30^. Trimming data resulted in an average read length of 82.8 bp across all samples, with a minimum read length of 25 bp, using *de novo* transcriptome assemblers. A total of 57,756,046 high-quality reads (4,780,938,320 bp) were obtained after combining the mycelium and mature fruiting body libraries of the dikaryon *L*. *edodes* strain SJ701 (ASI no. 3305) (Table 1). These reads were then assembled into 32,001 extended transcripts (k-mer = 51) with a mean length of 2,152 bp and N50 length of 2,900 bp (Table 2). The transcript length ranged from 200 to >4,500 bp (Supplementary Fig. S1). Finally, 11,675 loci were predicted from the extended transcripts, and the average length of the assembled loci was 1,702 bp with an N50 length of 2,443 bp (Table 2).

**Table 1:** Throughput and quality of Illumina sequencing of the *L*. *edodes* transcriptome.

Samples	Raw data		DynamicTrim data^a^		LengthSort data^b^	
	Mycelium	Fruiting body	Mycelium	Fruiting body	Mycelium	Fruiting body
Total reads	33,810116	37,248796	33,810116	37,248,796	27,646,278	30,109,768
Total length (bp)	3,414,821,716	3,762,128,396	2,464,553,530	2,691,940,823	2,296,017,777	2,484,920,543
Avg. length	101	101	72.91	72.26	83.06	82.50

^a^Quality value: each read was trimmed individually based on a quality score of less than 20.

^b^Standard minimum read length: reads from which low-quality regions were trimmed, and reads with 25 base pairs (bp) were filtered out.

**Table 2:** Functional annotation statistics of the *L*. *edodes* assembly data.

Data	Num. of transcripts	Length of transcripts (bp)				
		Total	Min	Max	Average	N50^a^
Total transcripts^b^	32,001	68,886,143	200	13,637	2,152	2,900
Representative transcripts^c^	11,675	19,872,247	200	13,637	1,702	2,443

^a^Half of all bases are in transcripts at least as long as N50.

^b^Total number of transcripts assembled using k-mer 51.

^c^Transcripts of multi-copy genes are collapsed into a single sequence.

The genome size of *L*. *edodes* ranged from 41.8 to 46.1 Mb^25,26^. As a result, approximately 4.78 Gbp of transcriptome sequence data represented at least 103-fold coverage. A reference-based assembly should reconstitute full-length transcripts with more than 10-fold coverage, and higher coverage is considered more challenging^31^. Therefore, the high coverage in this study will allow for more accurate assembly of the transcriptome. According to a recent report, 13,028 genes in *L*. *edodes* have been predicted by whole genome *de novo* sequencing and genome annotation^26^. Despite identifying approximately 89.6% (11,675) of these previously predicted genes (13,028), our results will allow for further annotation and characterization of the dikaryotic *L*. *edodes* strain.

### Functional annotation and classification of *L*. *edodes* transcripts

**Figure 2:**
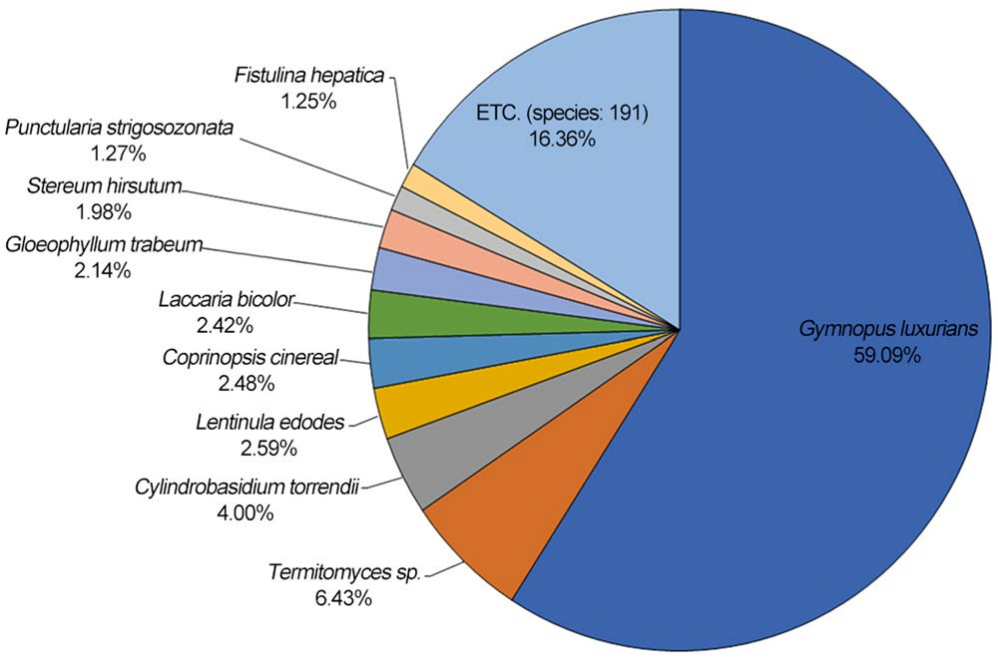
Species distribution of the BLASTx results. Species distribution of BLASTx results against the NCBI non-redundant protein database (e-value < 1e^−10^) and the proportions for each species are shown. Different colors represent different species, and the top 10 species are shown. Species with proportions >1% are shown.

Genome annotation and functional classification of 9,092 unigenes (77.9% of 11,675) were determined from the six datasets for the *L*. *edodes* SJ701 strain. All unigenes were aligned against sequences from each gene database using the BLASTx algorithm (e-value ≤ 1e^−10^). A total of 8,980 (98.8%) unigenes were found to match known proteins in the NR database, as well as the UniProtKB fungi (8,190, 90.1%), GO (5,834, 64.2%), KOG (4,904, 53.9%), KEGG (1,717, 18.9%), and InterProscan (1,231, 13.5%) databases (Supplementary Table S1). The annotated unigenes with BLASTx alignments in the NR database showed the highest homology to Basidiomycota *Gymnopus luxurians* FD-317 M1 (accession code, JJNP00000000; Bioproject, PRJNA68535)^32^, with 5,306 matching genes (59.1%). The distribution of proportional sequences matching the remaining top nine Basidiomycota that produce mushrooms (*Termitomyces* sp. *J132*, *Cylindrobasidium torrendii*, *L*. *edodes*, *Coprinopsis cenereal*, *Laccaria bicolor*, *Gloeophyllum trabeum*, *Stereum hirsutum*, *Punctularia strigosozonata*, and *Fistulina hepatica*), are shown in Fig. 2 and Supplementary Table S2. Despite *de novo* characterization of the *L*. *edodes* transcriptome^16,17,19,25^, the majority of the 11,675 representative transcripts did not match to known proteins (2.59%) because of a lack of *L*. *edodes* genomic information in the GenBank open access database.

To assess the enrichment of DEGs between *L*. *edodes* mycelia and mature fruiting bodies, we performed Gene Ontology (GO), Eukaryotic Orthologous Groups (KOG), and Kyoto Encyclopedia of Genes and Genomes (KEGG) analyses. The transcripts were functionally classified as follows. First, GO enrichment analysis is a bioinformatics tool used to annotate genes and gene products to terms in specific ontologies. GO terms are composed of three primary ontologies: biological process, cellular component, and molecular function^33^. To classify the functions of the annotated unigenes based on those in other fungi, 5,834 of the 9,092 unigenes were classified into the three primary GO categories and 18 functional subcategories, with 6, 6, and 6 annotated to biological process, cellular component, and molecular function, respectively, at an ontology depth score of 1^34^ (Supplementary Table S3). To investigate further, the level of differentiated depth was used for GO classification, unlike the depth of other reported results in *L*. *edodes*^19,27^. A total of 5,834 unigenes were assigned to at least one GO term. Cellular process (2,698 unigenes; 45.8%) and metabolic process (2,415; 41.0%) were the most common terms in the various biological process subcategories, cell (2,479; 75.3%) and membrane (749; 22.8%) were the most common terms in the cellular component category, and catalytic activity was the most common term in the molecular function category (Supplementary Table S3). These five GO terms (88.8% of all GO terms) represented the majority of the subcategories in the GO classification. Furthermore, the major processes represented by the *L*. *edodes* GO terms were consistent with previously reported results of comparisons between mycelia and fruiting bodies in *A*. *aegerita*, *A*. *polytricha*, *Hypsizygus marmoreus*, and *Pleurotus tuoliensis*^21,22,35,36^. In contrast, GO functional classification in two different studies^19,27^, which simply characterized the mycelia of a single strain and compared gene expression based on three different stages of fruiting body growth in *L*. *edodes*, showed significantly different results, despite the application of similar transcriptomic analyses. Thus, the current study is the first to demonstrate that *L*. *edodes* gene expression is regulated very differently depending on the developmental stage.

Second, to further analyze the annotations and transcriptome, the KOG database^36,37^ was used to assign the unigenes to 25 classifications for the first time in *L*. *edodes*. A total of 4,904 unigenes were assigned to at least one term in the 25 KOG functional categories (Supplementary Table S4). The top five KOG categories included ‘posttranslational modification, protein turnover, chaperones’ (O; 429, 8.7%), ‘signal transduction mechanisms’ (T; 358, 7.3%), ‘secondary metabolites biosynthesis, transport, and catabolism’ (Q; 299, 6.1%), ‘translation, ribosomal structure, and biogenesis’ (J; 292, 6.0%), ‘nuclear structure’ (X; 292, 6.0%) with the exception of the largest KOG group, and ‘general function prediction only’ (R; 788, 16.1%) (Supplementary Table S4). The top 10 KOG categories were matched to at least seven of the clusters of orthologous groups previously reported in *L*. *edodes* with respect to the Basidiomycota *A*. *aegerita*, *A*. *polytricha*, *H*. *marmoreus*, and *P*. *tuoliensis*^19,21,22,27,35,36^. However, the ranking of annotated categories differed. Interestingly, KOG classifications showed a higher similarity among other studies of *L*. *edodes* if several comparable unigenes were used for the clusters of orthologous group or KOG annotations.

Finally, to identify biological pathways in *L*. *edodes*, the 11,675 transcript sequences were searched in the KEGG database, and 1,717 unigenes were assigned to KEGG pathways, which included 4 main categories with 18 subcategories (98 pathways). The pathways consisted of metabolism- (875 unigenes, 51.0%), genetic information processing- (586, 34.1%), cellular processes- (166, 9.7%), and environmental information processing-related (85, 5.0%) functional categories, which are associated with the maintenance of fundamental biological pathways (Supplementary Table S5). Among the major categories, metabolic pathways, especially carbohydrate and amino acid metabolism, were active in *L*. *edodes*, similar to other mushrooms including *A*. *aegerita*, *A*. *polytricha*, and *G*. *lucidum*, in which comparative transcriptome analyses between mycelia and mature fruiting bodies have been performed^21,22,24^. Surprisingly, active subcategories of the KEGG annotated results based on transcriptome analysis data from the mycelium and mature fruiting body were more similar to those of the same stage in other mushrooms^21,22,24^ than to different stages of *L*. *edodes*^19,27^. The KEGG analysis results were similar to those of the GO functional analysis in this study, suggesting that KEGG annotation and analysis are valuable for exploring DEGs during different developmental stages. This observation has not been previously described in transcriptome analyses of the mycelial stage and the three stages of fruiting body growth in *L*. *edodes*. Therefore, the GO, KOG, and KEGG analyses in this study will be useful for further expression profiling and determination of DEGs, and these analyses are significant compared with previously reported results in *L*. *edodes*.

### Analysis of DEGs involved in the different developmental stages of *L*. *edodes*

**Figure 3:**
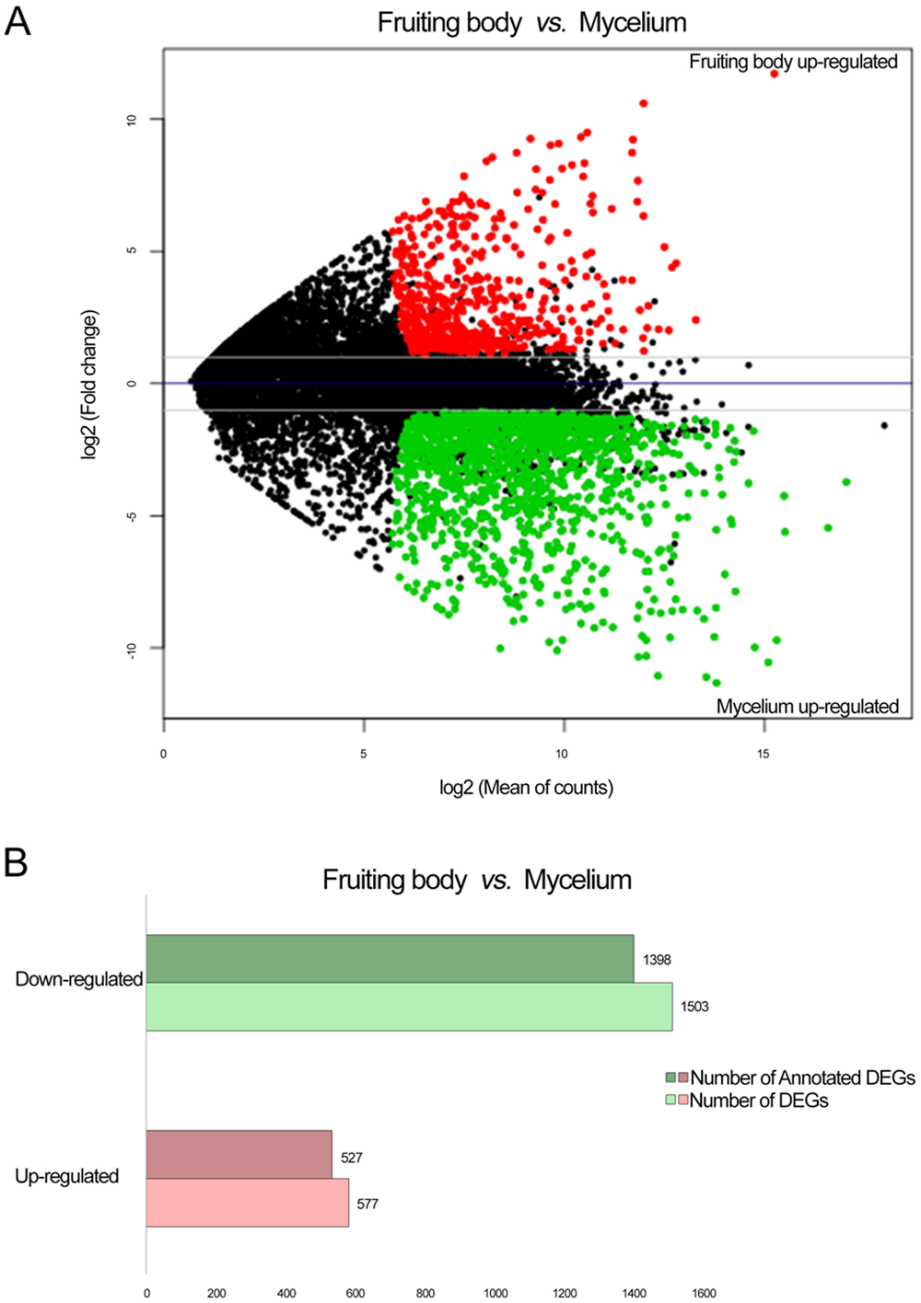
Summary of the differentially expressed genes (DEGs) between the mycelium and maturing fruiting body of *L*. *edodes*. (**A**) MA plot showing the means (*x*-axis) and log_2_ ratios (*y*-axis) of differential expression (adjusted p-value less than the significance level) during different developmental stages, based on normalized RNA-seq read counts. Each data point represents a transcript, and those indicating differential expression are colored in red and green. Gray dashed lines indicate thresholds specified for the fold change. (**B**) Estimates of significantly up-regulated and down-regulated genes in the mycelium versus fruiting body are indicated as a number distribution in each comparison.

**Figure 4:**
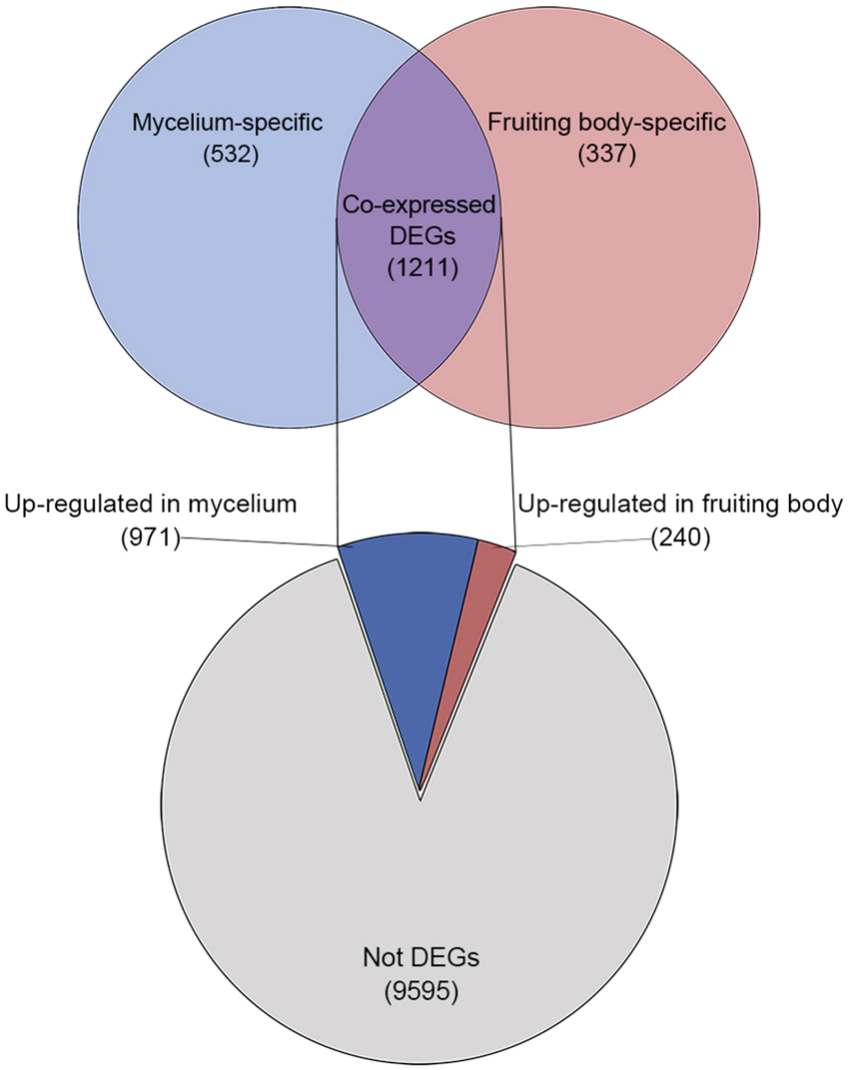
Venn diagrams showing the intersection of significant DEGs between the mycelium and maturing fruiting body in *L*. *edodes*. The numbers of up-regulated and down-regulated genes are summarized relative to the number of DEGs between the two groups in the RNA-seq dataset (false discovery rate ≤ 0.01, log_2_ ratio ≥ 2).

To identify DEGs between the dikaryotic mycelium and mature fruiting body of *L*. *edodes*, the expression level of each unigene from the two developmental stages was firstly calculated using the DESeq package^38^ and Another Multidimensional Analysis Package^39^, setting a threshold value of log_2_ ratio ≥ 1 and false discovery rate ≤ 0.01. The MA (scatter plot of the log_2_ fold change *vs*. the mean count) plot of pairwise comparisons is shown in Fig. 3A, in which red and green dots indicate the detected DEGs. Of 11,675 unigenes, 2,080 were significantly differentially expressed between the two stages in *L*. *edodes*, with 1,503 and 577 unigenes upregulated in the mycelium and mature fruiting body, respectively (Fig. 3B), and 1,211 unigenes expressed in both stages (Fig. 4). In total, 532 and 337 unigenes were specifically expressed in the mycelium and mature fruiting body, respectively. Overall, 9,595 unigenes showed significant changes in expression between the dikaryotic mycelium and mature fruiting body (Fig. 4). This expression profile is inconsistent with previous reports of DEGs detected in the dikaryotic mycelium and primordium by serial analysis of gene expression (SAGE)^28^, fruiting body formation phases by RNA sequencing (RNA-Seq)^19^, and mycelia and/or fruiting bodies cultured under different conditions^16,25^ due to large differences in gene regulation. The expression conditions were also inconsistent. Similar numbers of stage-specific DEGs of the mycelia and primordia were found in a gene profiling analysis using SAGE^28^. Different numbers of DEGs in three different stages during fruiting body growth were observed in a pairwise comparison of GO annotations using high-throughput RNA-Seq; in particular, genes were more highly upregulated in the mature fruiting body than in the primordium^19^. However, no genetic information related to DEGs of the mycelium and fruiting body has yet been reported. Therefore, our results necessitate further exploration of *L*. *edodes* DEGs in future studies.

**Figure 5:**
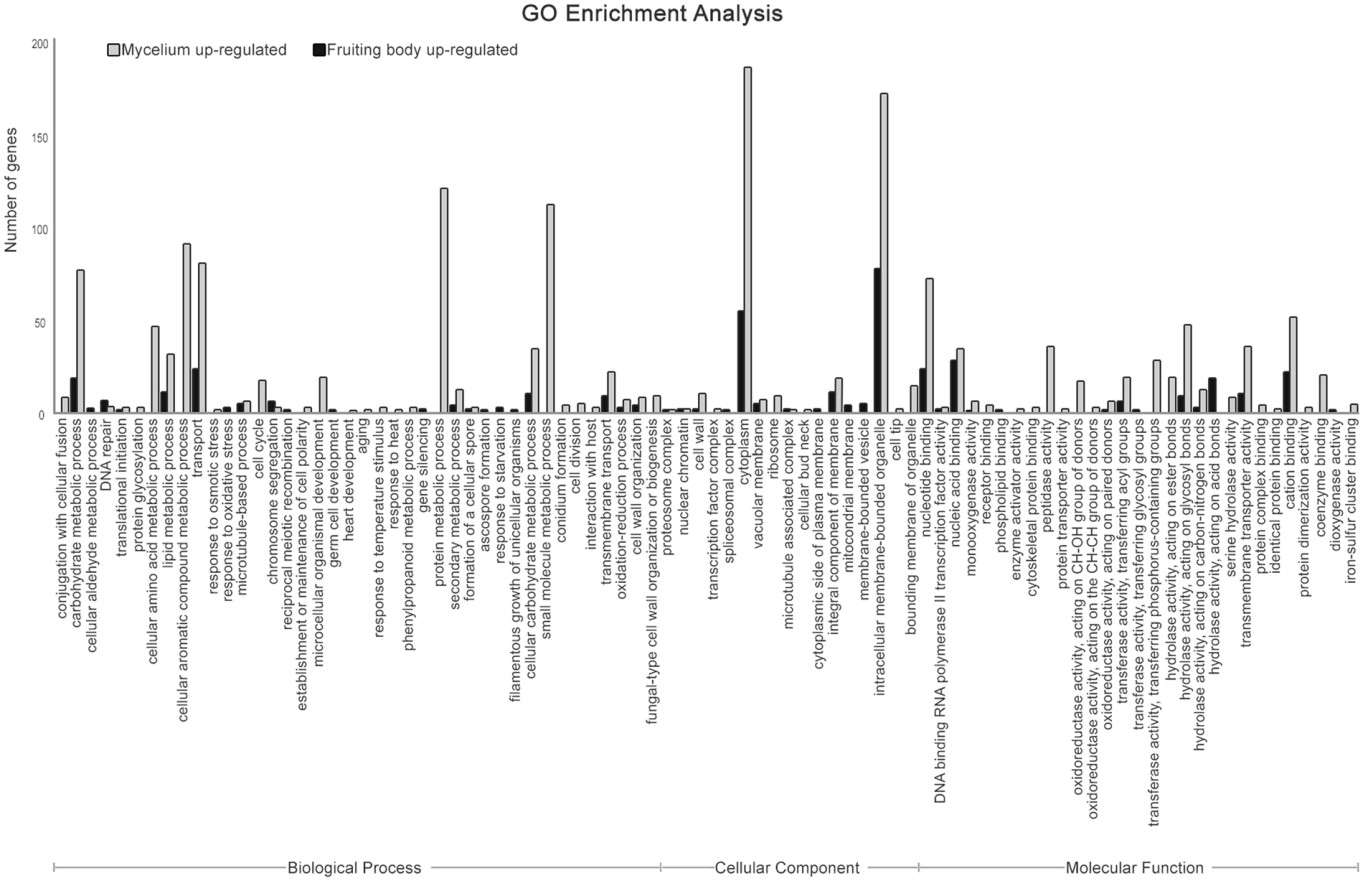
Gene ontology (GO) classification of the *L*. *edodes* transcriptome and DEGs between the mycelium and mature fruiting body. *L*. *edodes* unigenes were annotated to three functional categories: biological process (BP), cellular component (CC), and molecular function (MF). Bars indicate the number of genes in each GO category. The black and gray bars represent the up- and down-regulated DEGs of the mature fruiting body in *L*. *edodes*, respectively.

To investigate the function of the DEGs, the identified genes were annotated by GO, KOG, and KEGG. GO enrichment analysis provided functional GO terms that were substantially enriched in DEGs compared with the genome background. The results indicate that the DEGs were connected to interesting biological functions. To obtain detailed genetic information on the DEGs using the GO database, we classified the three primary GO categories into 86 functional subcategories, and 40, 17, and 29 unigenes were annotated in the biological process, cellular component, and molecular function categories, respectively, at an ontology depth score >3 (Fig. 5). Interestingly, the number of unigenes with GO annotations upregulated in the maturing fruiting body (407) was significantly less than the number of downregulated unigenes (1597) as transcriptomic studies in the same stage of *C*. *militaris*^23^. The number of DEGs annotated to the three primary GO terms was greater in the mycelium than in the mature fruiting body. In particular, the number of DEGs involved in biological processes was increased more than six-fold in mycelia (735) compared with the mature fruiting body (116) (Supplementary Table S6). These results suggest that the gene expression profile varies widely during mycelial growth compared with fruiting body maturation in *L*. *edodes*. Because no reports of GO enrichment analyses to compare the mycelium and fruiting body during any growth stage have been published, our results are novel and will be useful in the future exploration of DEGs in *L*. *edodes*.

GO enrichment analysis revealed that the cellular component ‘intracellular membrane-bounded organelle’ (248; 12.4% of 2,004 DEGs) and ‘cytoplasm subcategories’ (239; 11.9%) were most strongly enriched during both developmental stages. Although the terms ‘protein metabolic process’ (120; 7.5% of mycelial DEGs), ‘small molecule metabolic process’ (111; 7.0%), and ‘cellular aromatic compound metabolic process’ (90; 5.6%) in the biological process category were over-represented among the mycelial DEGs, no DEGs in mature fruiting bodies belonged to these three subcategories. ‘Peptidase activity’ (35 DEGs), ‘transferase activity, transferring phosphorus-containing groups’ (28), and ‘coenzyme binding’ (20) in the molecular process GO category were represented among the mycelial DEGs; however, no DEGs in the mature fruiting body were annotated to these subcategories. In contrast, DEGs involved in ‘hydrolase activity, acting on acid anhydrides’ (18), ‘membrane-bounded vesicle’ (5), ‘mitochondrial membrane’ (4), ‘response to oxidative stress’ (3), and ‘response to starvation’ (3) were expressed only in the mature fruiting body, according to the GO enrichment analysis (Fig. 5 and Supplementary Table S6). Hydrolase activity is important in cellular and subcellular movement, such as catalyzing transmembrane movement of substances during development of the fungal fruiting body^40,41^. Mitochondria also play a critical role in cell differentiation^42^, and mitochondrial processing enzymes are regulated during formation of the fruiting body^43^. Furthermore, a higher level of oxidative stress and/or nutritional starvation trigger mushroom development in various basidiomycota^35,44–46^. The results of this study suggest that mitochondrial processing, cell remodeling, oxidative stress, and starvation are more active in fruiting body development of the basidiomycete *L*. *edodes*.

**Figure 6:**
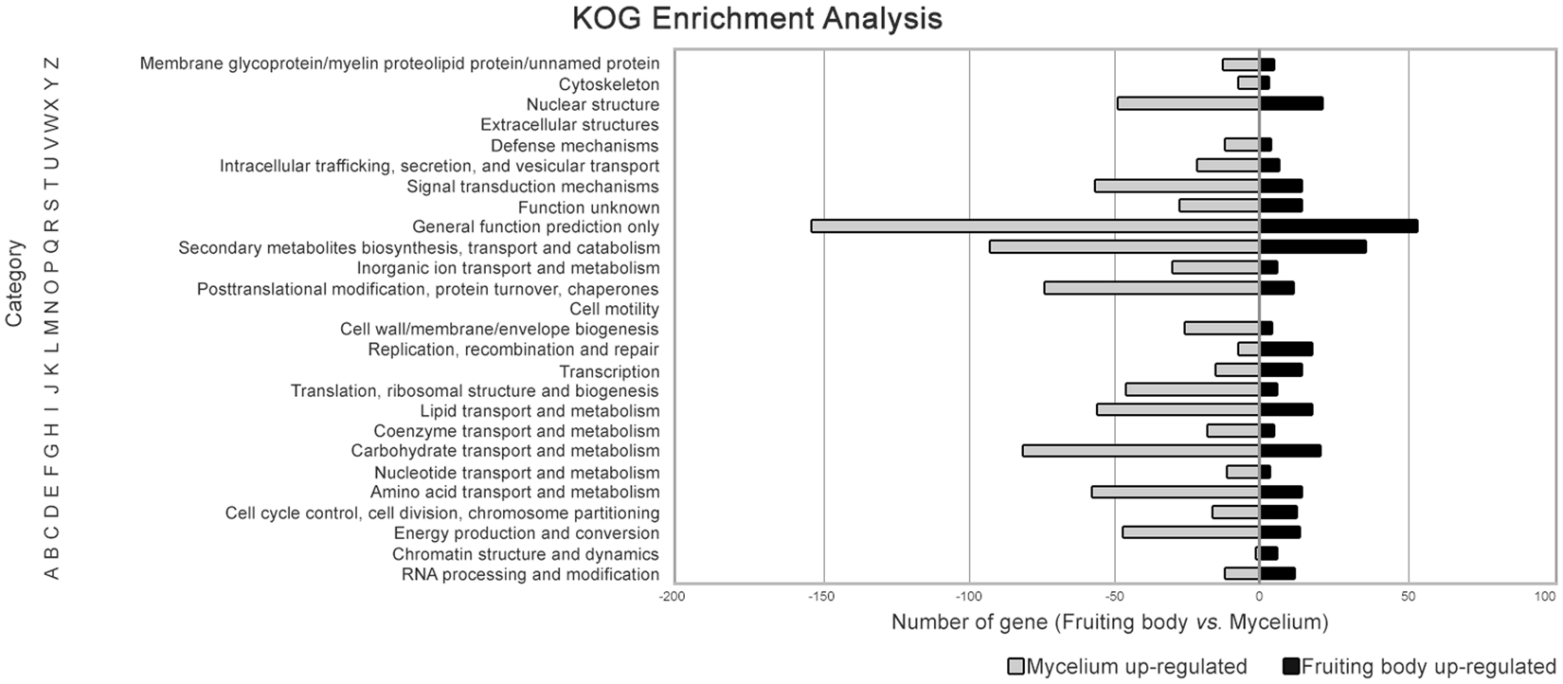
Functional annotation of DEGs in accordance with euKaryotic Orthologous Groups (KOG) categories. All development stage-associated DEGs were assigned to the 26 KOG functional categories using BLASTx. Bars indicate the number of genes in each KOG category that revealed significant changes in expression (log_2_ ratio ≥ 2) between the two developmental stages in *L*. *edodes*. The black and gray bars represent the up- and down-regulated DEGs of the mature fruiting body in *L*. *edodes*, respectively.

Enrichment analysis of KOG functional categories was performed to further investigate the difference in classification depending on the developmental stage (Fig. 6 and Supplementary Table S7). This result has not been described previously. The number of upregulated DEGs was smaller in the maturing fruiting body (305) than in the mycelium (925), similar to the results of the GO enrichment analysis. The top five KOG categories, ‘general function prediction only’ (10.3% of DEGs in the KOG database), ‘secondary metabolite biosynthesis, transport, and catabolism’ (9.8%), ‘carbohydrate transport and metabolism’ (8.2%), ‘amino acid transport and metabolism’ (5.8%), and ‘signal transduction mechanisms’ (5.7%) were over-represented in both stages. In contrast, none of the DEGs showed matches in the KOG database search of the ‘cell mortality’ and ‘extracellular structure’ categories. The numbers of DEGs in 22 KOG categories were greater in the mycelium than the mature fruiting body, except for the categories ‘replication, recombination, and repair’ and ‘chromatin structure’. Development of the fruiting body is preceded by premeiotic replication, karyogamy, and meiosis, and thus genes related to these processes are overexpressed during the maturation stages^18,47,48^. These results suggest that genes involved in DNA replication, recombination, repair, chromatin structure, and dynamics are overexpressed during the fruiting body stage.

**Figure 7:**
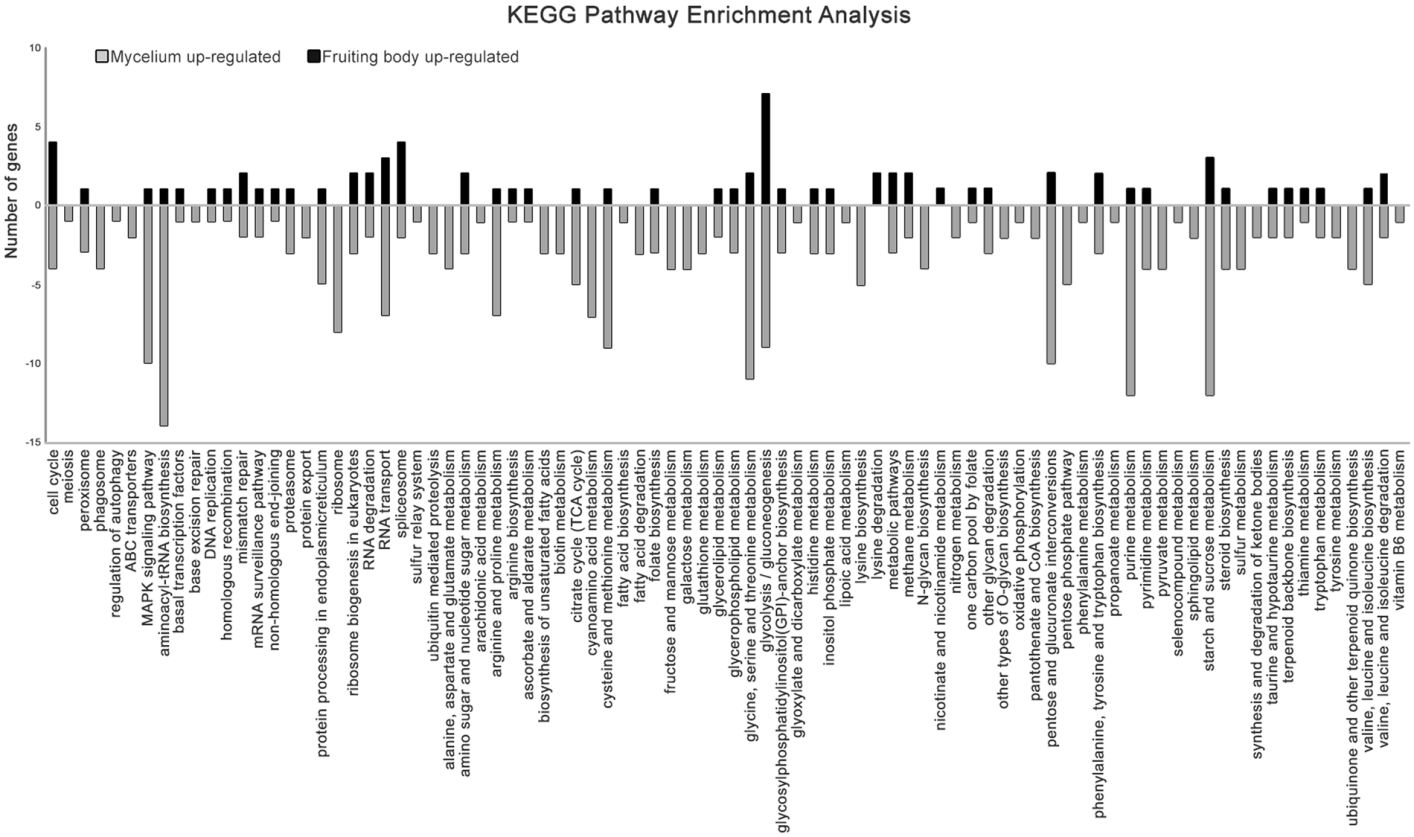
KEGG pathway analysis of the DEGs. Bars indicate the number of genes in each KEGG pathway that revealed significant changes in expression (log_2_ ratio ≥ 2) between the two developmental stages in *L*. *edodes*. The black and gray bars represent the up- and down-regulated DEGs of the mature fruiting body in *L*. *edodes*, respectively.

To further evaluate the functional pathways involved in the fruiting body, all DEGs were matched to the KEGG database (Fig. 7 and Supplementary Table S8). KEGG sub-classification analysis showed that a greater number of DEGs were involved in carbohydrate and amino acid metabolism and translation, which represent pathways involved in the maintenance of basic biological processes during both stages^22^. Among the KEGG sub-classifications, the number of upregulated DEGs was decreased in the maturing fruiting body compared with the mycelium, consistent with the GO and KOG enrichment analyses. The majority of the sub-classifications, including ‘nucleotide metabolism’, ‘metabolism of other amino acids’, ‘glycan biosynthesis and metabolism’, ‘metabolism of cofactors and vitamins’, ‘energy metabolism’, ‘lipid metabolism’, ‘folding, sorting, and degradation’, ‘transport and catabolism’, and ‘signal transduction’, among all 18 KEGG sub-classifications including the above 3 sub-classifications were over-represented in the mycelium stage (Fig. 7). On the other hand, there was little difference in the number of DEGs in the ‘metabolism of terpenoids and polyketides’, ‘biosynthesis of other secondary metabolites’, ‘membrane transport’, and ‘cell growth and death’ sub-classifications. Moreover, the ‘replication and repair’ and ‘transcription’ sub-classifications were uniquely enriched in the mature fruiting body stage (Fig. 7).

In addition, KEGG pathway analysis was used to compare the differences between the DEGs of both stages to identify a more definitive biological pathway represented among the sub-classifications. Nearly all pathways identified in the KEGG enrichment analysis were more associated with the DEGs in the mycelium than those in the mature fruiting body. Only 6 of the 86 pathways showed a higher number of DEGs in the mature fruiting body compared with the mycelium. In particular, genes involved in ‘replication and repair’, as well as the ‘mismatch repair’, ‘homologous recombination’, ‘non-homologous end-joining’, and ‘spliceosome of transcription’, were more active in the mature fruiting body compared with the mycelium, similar to the results of the KOG enrichment analysis (Supplementary Table S8). These results are consistent with those described in previous reports, in which genes participating in DNA replication, recombination, repair, chromatin structure, and the associated dynamics were found to be important in development of the fruiting body. As reported previously, spliceosome pathways were was also enriched in the fruiting body of *C*. *militaris*^23^. However, the findings of the present study are distinct from previous transcriptome analyses conducted in other mushrooms comparing the mycelium and fruiting body^21–24^. Gene expression patterns vary widely during mycelial growth compared with fruiting body maturation in *L*. *edodes*. Hence, it is possible that downregulation of gene expression is required for mature fruiting bodies in the *L*. *edodes* genome, and information regarding fruiting body-related genes is currently limited in the available databases.

### Special genes involved in mature fruiting body development in *L*. *edodes*

Analysis of the DEGs provides valuable information regarding maturation of the fruiting body, which is initiated by the presence of a stimulus that activates specific genes that enable development of the mycelium into a fruiting body^18,21–24^. Analysis of the DEGs allowed us to search for marked differences in the gene expression profiles of the mycelium and fruiting body in *L*. *edodes*. The log_2_ fold change values between the two stages ranged from −11.3 to 11.7, and DEGs showing a greater than twofold change between the two stages are presented in Supplementary Table S8. Overall, 577 unigenes were upregulated in the fruiting body, including 240 co-expressed and 337 fruiting body-specific unigenes (log_2_ ratio ≥ 1) (Fig. 4). Of the 337 stage-specific unigenes, 53 unigenes were exclusively overexpressed in the mature fruiting body of *L*. *edodes*. The overexpressed fruiting body-specific genes were selected based on read counts of <50 in the mycelium and ≥500 in the fruiting body. According to the cutoff levels, 51 unigenes were selected as mature fruiting body-specific candidate genes; these unigenes were identified and shown to be involved in fruiting body maturation (Table 3). Overexpressed genes were selected as reference data based on read counts <50 in the fruiting body and ≥500 in the mycelium. At these cut-off levels, 172 unigenes were selected as mycelium-specific candidate genes that will be further examined to understand stage-specific expression of functional properties of *L*. *edodes* (Supplementary Table S9).

**Table 3:** Functional annotation of specific DEGs in the mature fruiting body.

Unigene ID	Mycelium read count	Fruiting body read count	Log2 fold change^a^	Description	Species^b^	Evalue	Identity
LE_007412	5	8,057	10.60	Hydrophobin 1	*L*. *edodes*	2.00E-34	99.21
LE_009311	35	7,282	7.67	Hypothetical protein GYMLUDRAFT_39929	*G*. *luxurians* FD-317 M1	2.00E-47	43.02
LE_007730	11	6,695	9.23	Aspartic protease	*L*. *edodes*	0	98.78
LE_006927	15	6,593	8.73	MFS transporter	*G*. *trabeum* ATCC 11539	2.00E-164	41.88
LE_007440	47	4,597	6.61	Heat shock protein 9	*L*. *edodes*	1.00E-53	100.00
LE_003624	37	3,315	6.48	Hypothetical protein	*L*. *edodes*	0	89.00
LE_007464	24	3,310	7.10	Gamma-glutamyl transepetidase	*L*. *edodes*	0	91.85
LE_006999	28	3,192	6.80	DNA mismatch repair protein	*G*. *frondosa*	6.00E-90	61.46
LE_007861	4	3,037	9.49	GPI-anchor protein	*G*. *luxurians* FD-317 M1	1.00E-29	54.90
LE_007450	9	2,890	8.34	Cyclohexanone monooxygenase	*L*. *edodes*	0	97.00
LE_007442	12	2,826	7.83	O-acetylhomoserine (thiol)-lyase	*R*. *solani* AG-1 IB	6.00E-180	64.80
LE_011335	4	2,723	9.32	Hypothetical protein GYMLUDRAFT_486395	*G*. *luxurians* FD-317 M1	0	68.95
LE_006542	7	2,330	8.26	Protein kinase	*F*. *hepatica* ATCC 64428	9.00E-67	39.38
LE_006982	40	2,113	5.70	Amidotransferase	*S*. *hirsutum* FP-91666 SS1	1.00E-160	44.00
LE_006156	7	1,962	8.13	GPI-anchor protein	*L*. *bicolor* S238N-H82	1.00E-20	32.45
LE_007727	3	1,853	9.08	Hypothetical protein GYMLUDRAFT_73314	*G*. *luxurians* FD-317 M1	4.00E-125	52.70
LE_006715	15	1,726	6.79	Cysteine hydrolase	*L*. *edodes*	1.00E-175	99.00
LE_007912	3	1,612	9.01	Hypothetical protein	*C*. *hominis* TU502	1.00E-128	100.00
LE_007695	7	1,586	7.70	Hypothetical protein GYMLUDRAFT_944329	*G*. *luxurians* FD-317 M1	3.00E-22	63.77
LE_006892	34	1,580	5.52	Hypothetical protein	*L*. *sulphureus 93-53*	3.00E-06	56.10
LE_005107	36	1,530	5.40	Transcription factor	*L*. *edodes*	0	88.12
LE_007489	19	1,396	6.19	Hypothetical protein PLICRDRAFT_103741	*P*. *crispa* FD-325 SS-3	2.00E-93	55.29
LE_007467	9	1,385	7.22	Hypothetical protein GYMLUDRAFT_89343	*G*. *luxurians* FD-317 M1	4.00E-80	60.74
LE_007396	22	1,266	5.83	Cytochrome P450	*L*. *edodes*	0	86.68
LE_007448	4	1,253	8.11	Cytochrome P450	*L*. *edodes*	0	100.00
LE_005239	7	1,240	7.33	Hypothetical protein GYMLUDRAFT_39615	*G*. *luxurians* FD-317 M1	3.00E-120	69.20
LE_007459	2	1,138	9.26	Glycoside hydrolase family 61	*G*. *luxurians* FD-317 M1	2.00E-54	63.87
LE_011349	11	1,085	6.59	Splicing factor	*L*. *edodes*	0	100
LE_006616	31	927	4.89	Hypothetical protein GYMLUDRAFT_265499	*G*. *luxurians* FD-317 M1	0	66.11
LE_007328	5	901	7.23	Hypothetical protein GYMLUDRAFT_172776	*G*. *luxurians* FD-317 M1		
LE_007951	2	894	8.73	Hypothetical protein	*L*. *edodes*	5.00E-109	96.57
LE_010885	13	844	6.00	Cyclin	*M*. *roreri* MCA 2997	3.00E-111	50.20
LE_006898	23	844	5.17	Reverse transcriptase-RNase H-integrase	*L*. *edodes*	0	99.00
LE_001650	49	746	3.92	ARM repeat-containing protein	*L*. *sulphureus* 93-53	0	94.63
LE_004420	16	740	5.46	Alcohol oxidase	*L*. *edodes*	0	98.43
LE_007446	16	733	5.51	Hypothetical protein			
LE_006790	8	682	6.24	Carbohydrate esterase family	*G*. *luxurians* FD-317 M1	0	82.79
LE_007462	7	670	6.44	Meiosis protein	*L*. *edodes*	0	97.83
LE_005246	17	608	5.11	DDR48-Heat shock protein	*L*. *edodes*	2.00E-106	87.76
LE_007461	10	605	5.89	MFS transporter	*A*. *gallica*	0	67.12
LE_006895	8	604	6.22	Hypothetical protein	*G*. *luxurians* FD-317 M1	3.00E-122	52.14
LE_001942	47	602	3.65	Alpha/beta-hydrolase	*L*. *edodes*	8.00E-113	92.27
LE_010744	40	601	3.90	MFS transporter	*L*. *edodes*	0	97.54
LE_007512	48	599	3.63	CIPA	*L*. *edodes*	3.00E-88	87.62
LE_007498	1	587	8.55	Aquaporin	*L*. *edodes*	3.00E-161	77.99
LE_005252	11	572	5.95	Hypothetical protein GYMLUDRAFT_775031	*G*. *luxurians* FD-317 M1	1.00E-156	57.88
LE_007439	11	544	5.52	Hypothetical protein	*L*. *edodes*	0	85.58
LE_007456	1	531	8.41	Phospholipase C	*L*. *edodes*	3.00E-16	54.65
LE_009426	42	531	3.64	Isopentenyl diphosphate isomerase	*L*. *edodes*	0	99.23
LE_006701	46	531	3.53	Glycosyltransferase family 2 protein	*M*. *chlorophos*	6.00E-93	65.40
LE_011394	5	525	6.72	Protein kinase	*L*. *edodes*	0	99.58
LE_005524	31	513	4.01	Lysine decarboxylase	*Phellinus noxius*	2.00E-65	49.35

^a^Log2 fold change indicates a significant difference between fruiting body gene counts and mycelium gene counts.

^b^*L. edodes, Letinula edodes*; *G. luxurians, Gymnopus luxurians*; *G. trabeum, Gloeophyllum trabeum*; *G. frondosa, Grifola frondosa*; *R. solani Rhizoctonia solani*; *F. hepatica, Fistulina hepatica*; *S. hirsutum, Stereum hirsutum*; *L. bicolor, Laccaria bicolor*; *C. hominis, Cryptosporidium hominis*; *L. sulphureus, Laetiporus sulphureus*; *P. crispa, Plicaturopsis crispa*; *M. roreri, Moniliophthora roreri*; *A. gallica, Armillaria gallica*; *M. chlorophos, Mycena chlorophos*; *P. noxius, Phellinus noxi*.

These mature fruiting body-specific unigenes were matched to 34 homologous proteins by BLASTx against the NR database, including 17 unmatched unigenes. All 34 unigenes showed significantly upregulated expression in the mature fruiting body (Table 3); however, the expression level and stage specificity differed compared with fruiting body specific-genes. The special 12 DEGs, encoding the homologous proteins aspartic protease (LE_007730), heat shock protein 9 (LE_007440), gamma-glutamyl transpeptidase (LE_007464), DNA mismatch repair protein (LE_006999), cyclohexanone monooxygenase (LE_007450), O-acetylhomoserine lyase (LE_007442), splicing factor (LE_011349), reverse transcriptase RNase H (LE_006898), cyclin (LE_010885), meiosis protein (LE_007462), ARM repeat-containing protein (LE_001650), and aquaporin (LE_007498), were only detected in the fruiting body of *L*. *edodes*. Moreover, DEGs encoding homologous protein(s) were not detected in the dikaryotic mycelium, suggesting that these proteins play a crucial role in fruiting body maturation in *L*. *edodes* but not at the mycelium stage (Supplementary Fig. 2). The other 22 DEGs, encoding hydrophobin 1 (LE_007412), MFS transporter (LE_006927, LE_007461, LE_010744), protein kinase (LE_006542, LE_011394), amidotransferase (LE_006982), cysteine hydrolase (LE_006715), transcription factor (LE_005107), cytochrome P450 (LE_007448, LE_007396), glycoside hydrolase family 61 (LE_007459), alcohol oxidase (LE_004420), carbohydrate esterase family (LE_006790), alpha/beta-hydrolase (LE_001942), CIPA (LE_007512), phospholipase C (LE_007456), GPI-anchor protein (LE_007861, LE_006156), isopentenyl diphosphate isomerase (LE_009426), glycosyltransferase family 2 protein (LE_006701), and lysine decarboxylase (LE_005524), were also exclusively expressed in the fruiting body (Table 3). However, there were additional homologous proteins in both developmental stages according to the profiles of the 11,675 DEGs. Although each gene was distinctly overexpressed in the fruiting body stage exclusively, several proteins with the same function existed in both stages. It seems likely that conspecific proteins play various roles in both stages, and not only in fruiting body maturation.

Among the 34 fruiting body maturation-specific DEGs, the LE_007730 unigene encodes an aspartic protease, which was expressed 609-fold (log_2_ ratio = 9.23) higher in the fruiting body than in the mycelium (Table 3). Many proteases are used commercially, and fungal proteases have been reported in various mushrooms, including *Agaricus bisporus*, *Armillariella mellaea*, *P*. *ostreatus*, and *Xylaria hypoxylon*^49–51^. A novel aspartic protease from *X*. *hypoxylon*, a unique enzyme with relatively high thermostability, has been reported to inhibit HIV reverse transcriptase^51^. In addition, the gamma-glutamyl transpeptidase (LE_007464, log_2_ ratio = 7.1), an important enzyme involved in flavor formation, is involved in the degradation of glutathione and glutathione-S-conjugates of xenobiotics and pharmaceuticals produced in detoxification processes^52^. Thus, gamma-glutamyl transpeptidase has been purified and characterized for further application in *L*. *edodes*^53^. Cyclohexanone monooxygenase (LE_007450, 8.34) is a valuable flavoenzyme that was initially isolated from *Acinetobacter* spp. and has been heterologously expressed for biocatalyst development^54^. Reverse transcriptase RNase H (LE_006898, 5.17) exhibits antifungal, antiviral, antitumor, and antiproliferative properties^8^. Ribonucleases, purified from the fruiting body of *Russula delca*, have been shown to exhibit antifungal and HIV-1 reverse transcriptase inhibitory activities. Aspartic protease (LE_007730), gamma-glutamyl transpeptidase (LE_007464), cyclohexanone monooxygenase (LE_007450), and reverse transcriptase RNase H (LE_006898) were expressed in the mature fruiting body stage only and not in the mycelium (Table 3). These results suggest that beneficial substances such as aspartic protease, gamma-glutamyl transpeptidase, cyclohexanone monooxygenase, and reverse transcriptase RNase H are produced optimally only during the mature fruiting body stage.

On the contrary, lectins are also abundantly expressed in the dikaryotic mycelium but not in the primordia of *L*. *edodes*^28^. Mushroom lectins have been reported to improve the antitumor and immunomodulatory activities of pharmaceutical substances^55^ and to stimulate mushroom development^28,56^. Among the identified DEGs, the LE_008126 and LE_000173 unigenes, which encode lectin homologs, were upregulated 376-fold (log_2_ ratio = 8.6) and 11.0-fold (3.5), respectively, in the mycelium compared with the fruiting body. The LE_008126 unigene in particular showed limited expression in the mature fruiting body (Supplementary Table S8). These results suggest that the expression of lectins is suppressed during fruiting body development as well as the primordia stage, and that lectins can be purified from mushroom mycelia to increase yield.

Among the identified 34 DEGs, the most significant expression difference was observed for the LE_007412 unigene, annotated as hydrophobin 1 (*hyd1*), which had 1,600-fold higher (log_2_ ratio = 10.6) expression in the fruiting body than the mycelium (Table 3). Expression of the *hyd1* gene was limited in the dikaryotic mycelium, whereas the LE_000457 unigene, annotated as hydrophobin 2 (*hyd2*), was abundantly expressed in the dikaryotic mycelium in *L*. *edodes*, consistent with a previous report^28^. It has been reported that hydrophobins are essential for fruiting body formation in various mushrooms^28,57,58^, and *hyd1* plays a more important role in fruiting body initiation (primordia) than maturation in *L*. *edodes*^57^. According to our analysis, this result suggests that *hyd1* not only functions in fruiting body initiation but also in maturation in *L*. *edodes*. The results also indicate the validity of our transcriptome analysis.

According to the stage-specific expression patterns of representative DEGs observed in this study, specific stages were verified as optimal for the production of beneficial nutritional and medicinal properties and new generation of improved *L*. *edodes* cultivars. Mycelium-specific DEGs were also listed in Supplementary Table S9. These useful substances are generally purified from mature *L*. *edodes* fruiting bodies and/or mycelia. No reports of comparative transcriptomic analyses of dikaryotic mycelia and mature fruiting bodies in *L*. *edodes* have been published. Thus, the large and accurate resource provided by our comparative transcriptomic analysis will contribute to increased production of *L*. *edodes* mushrooms with beneficial properties.

Finally, among 12 special DEGs for fruiting body maturation, DNA mismatch repair protein (LE_006999, 6.80), meiosis protein (LE_007462, 6.44), splicing factor (LE_011349, 6.59), ARM repeat-containing protein: RNA-binding protein (LE_001650, 3.92), heat shock protein 9 (LE_007440, 6.61), and cyclin: cell cycle control protein (LE_010885, 6.0) were involved in DNA replication, recombination, repair, and chromatin structure (Supplementary Fig. S2 and Table 3). These proteins are involved in premeiotic replication, karyogamy, and meiosis, which occur during fruiting body maturation of *L*. *edodes*^18,47,48^. Interestingly, these results are consistent with the KOG and KEGG enrichment analyses of DEGs in this study. As shown in Supplementary Table S10, we explored 31 *L*. *edodes* genes related to functions including DNA replication, recombination, mismatch repair, spliceosomes, and chromatin structure and dynamics, which are important for premeiotic replication, karyogamy, and meiosis during fruiting body maturation. These results provide valuable genetic information for further studies of the molecular mechanism of fruiting body maturation in this species. The identification and analysis of DEGs of *L*. *edodes* provides valuable information regarding fruiting body maturation and the use of functional substances in future genetic studies.

### Validation of transcriptome data by qRT-PCR

**Figure 8:**
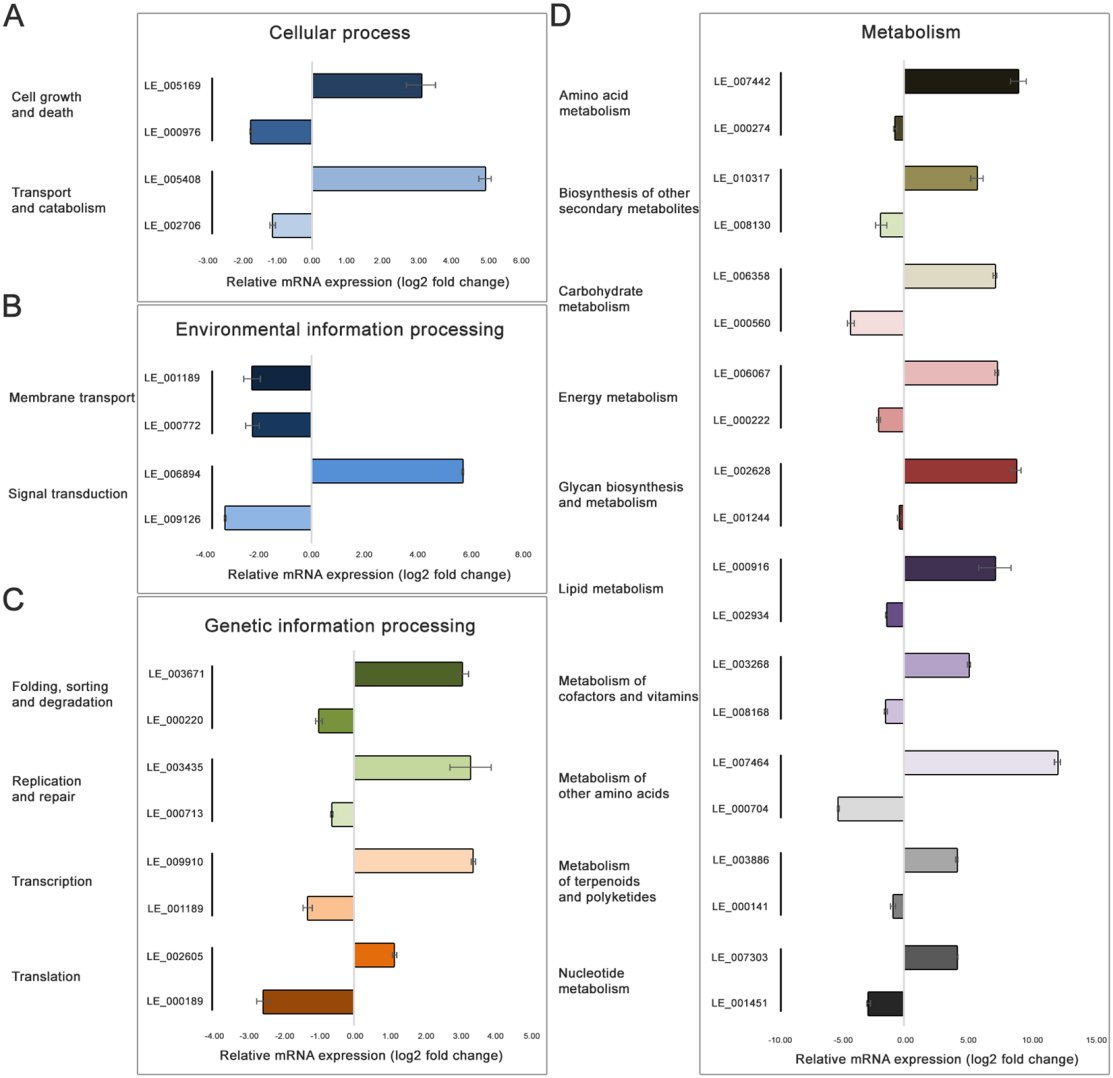
Validation of the RNA-seq results by quantitative real-time polymerase chain reaction. The log_2_ fold change is denoted as the ratio of expression in the fruiting body to that in the mycelium. The *y*-axis represents the expression levels of the genes relative to the housekeeping gene (18S rRNA) for three biological and technical replicates. Significant differences of *p* < 0.01 were analyzed using Student’s *t*-test.

Each DEG selected from *L*. *edodes* dikaryotic mycelia and mature fruiting bodies was analyzed by qRT-PCR. According to the KEGG annotation of genes differentially expressed between the dikaryotic mycelium and mature fruiting body, 36 DEGs from 18 KEGG subcategories in *L*. *edodes* were used to validate the results of the RNA-seq analysis (Fig. 8). One upregulated and one downregulated gene from each of the 18 KEGG subcategories were selected based on the significant fold changes in gene expression induced following formation of mycelia or mature fruiting body (Supplementary Table S11). The housekeeping gene 18S rRNA, used for qRT-PCR normalization^16^, was used as the reference gene, because the internal control gene was expressed at a constant level. These genes exhibited differential expression patterns similar to those in the RNA-seq experiments (Fig. 8). Therefore, DEGs identified by RNA-seq, which were validated by qRT-PCR, can be further investigated as candidate genes involved in the complex developmental stages of the mycelium and mature fruiting body.

## Conclusions

This study is the first high-throughput transcriptome sequencing analysis of DEGs comparing the dikaryotic mycelium with the mature fruiting body of the commercially important Korean mushroom species *L*. *edodes* to investigate specific genes and specific developmental stages for optimal yield of beneficial properties and new generation. Furthermore, it is the first transcriptome analysis of functional enrichment using GO, KOG, and KEGG classification of DEGs to generate extensive and accurate genetic information on *L*. *edodes* for further study. Interestingly, functional annotations and enrichment analyses of the DEGs indicated that the genes overexpressed in the mature fruiting body stage are significantly involved in DNA replication, recombination, repair, chromatin structure, and the associated dynamics and significantly enriched in ‘replication and repair’ and ‘transcription’ pathways for premeiotic replication, karyogamy, or meiosis in *L*. *edodes*. Furthermore, many putative genes were verified by sequence comparisons with known the sequences of fungal gene families. Future analyses of the expression profiles of homologs related to fruiting body maturation and commercially useful functional substances would be useful to further explore the molecular mechanisms of *L*. *edodes*. DEGs showing significant changes in expression between different developmental stages can be further investigated for exploitation in commercial maturation and assessing the properties of the fruiting body using functional analysis and genetic engineering. Our transcriptome data can also be applied to transcriptome-based evaluation of the *L*. *edodes* stress response to mycoviral infection for research in an unexplored field.

## Methods

### *L*. *edodes* culture conditions

The commercial dikaryotic *L*. *edodes* strain, Sanjo701, was obtained from the Forest Mushroom Research Center, Korea. Mycelia were cultured from a source of *L*. *edodes* (mycelia discs, r = 5 mm) on potato dextrose agar at 25 °C in the dark. After 10 days, fresh mycelia covered the cellophane surface on top of the medium (diameter = 9 mm), and 10 g of mycelia were collected^59^. The mycelia were ground, rehydrated in culture broth, and inoculated inside the top of a sterile plastic sawdust bag with oak tree sawdust (850 g) and rice bran (150 g) with 55% moisture content. The sawdust bags with cotton aeration filters were incubated for 30 days at 25 °C in the dark to permit vegetative growth. To allow for mycelial ripening and browning, the incubated bags were transferred to a 20 °C greenhouse under natural light conditions for an additional 30 days. Next, the top piece of the bag, in which mycelia extended through more than half of the sawdust as a brown film, was cut and removed, and the bag was sprinkled with water for 12 h. The wet medium was maintained at 20 °C and >70% humidity to produce mature fruiting bodies on different sawdust bags. Fresh fruiting bodies were harvested immediately and stipes were removed to the extent possible^19^. Pilei with gills were frozen at −80 °C for use as representative samples of the mature fruiting bodies. *L*. *edodes* samples from the two developmental stages, the mycelium and fruiting body, were prepared for RNA extraction for further RNA-Seq and qPCR analysis.

### RNA isolation and cDNA library construction

Biological samples of total RNA were purified in triplicate from the mycelia and fruiting bodies of *L*. *edodes* as described previously^59^. RNA quality and quantity were evaluated using the Agilent 2100 BioAnalyzer with an RNA integrity value greater than seven (Agilent Technologies, CA, USA). We constructed a total of six libraries for RNA sequencing (RNA-Seq) using the Illumina TruSeq RNA sample preparation kit (Illumina, CA, USA), in accordance with the manufacturer’s instructions. mRNA, which was enriched by poly A tail selection and chemically fragmented, was used for first-strand cDNA synthesis using a random hexamer primer, followed by second-strand cDNA synthesis. The cDNA fragments were blunted with adenine nucleotides and connected with sequencing adaptors. The libraries were then size-selected for the cDNA target fragments, and the selected cDNA was enhanced via polymerase chain reaction (PCR) using adaptor-specific primers. The cDNA library was quantified using the KAPA library quantification kit (Roche, Switzerland) according to the manufacturer’s instructions.

### Illumina sequencing and *de novo* assembly

High-throughput sequencing of each cDNA library from the mycelium and a mature fruiting body was performed in triplicate using the HiseqTM 2000 system (Illumina) to ensure the desired average sequencing depth. The 101-base pair (bp) raw paired-end reads were filtered according to the Phred quality score (Q ≥ 20) and read length (≥25 bp) using SolexaQA software (http://solexaqa.sourceforge.net/) to obtain high-quality clean reads by adaptor removal^30^. *De novo* assembly of the trimmed reads was performed using the Velvet (v1.2.10)^60^ and Oases (v0.2.09) programs^61^, which implement the de Bruijn graph algorithm. We considered several hash lengths to determine optimal *de novo* assembly.

Raw sequencing data of three independent biological replicates for RNA-Seq were deposited in the National Center for Biotechnology Information (NCBI) sequencing read archive (https://submit.ncbi.nlm.nih.gov/subs/sra/) under accession number SRP137701.

### Short read mapping and expression profiles

Trimmed reads for each sequence tag were mapped to the assembled transcripts using Bowtie2 (v2.1.0) software^62^. The number of mapped reads for each unique transcript was calculated using an in-house script. Gene expression datasets were generated from each of two differential stages and raw counts were normalized and analyzed using the DESeq package in the R software^38^. The fold change (FC) and number of reads mapped to each unigene were used to identify DEGs between the two stages. A false discovery rate (FDR) was applied to calculate the p-value threshold for statistical significance in multiple-comparison tests. We used ‘FDR ≤ 0.01 and | log_2_FC | ≥ 1’ as the threshold to assess the significance of gene expression differences. Hierarchical clustering for all correlation analyses was performed using the Another Multidimensional Analysis Package library in R^39^. Upregulated and downregulated transcripts were subjected to Venn diagram analysis.

### Functional annotation

All assembled transcripts from the total reads were validated by direct comparison using the fungi non-redundant (NR) (https://www.ncbi.nlm.nih.gov/refseq) and UniProtKB (http://www.ebi.ac.uk/uniprot) databases and the euKaryotic Orthologous Groups (KOG) tool (http://www.ncbi.nlm.nih.gov/KOG) using BLASTx (e-value ≤ 1e^−10^). Proteins with high sequence similarity were retrieved for analysis. Gene ontology (GO) and Kyoto Encyclopedia of Genes and Genomes (KEGG) analyses were carried out using the sequence similarities (e-value ≤ 1e^−10^) of proteins in the GO^33^ and KEGG^63^ databases. For each list of DEGs, down- and up-regulated genes were annotated using GO classification based on protein sequence similarity in the GO database. The number of genes assigned to GO terms was counted using in-house scripts from Seeders Co. (Korea).

### Real-time quantitative (qRT) PCR analysis

cDNA was synthesized from approximately 1 μg of total RNA from the mycelium and mature fruiting body excluding the stipe using the GoScript Reverse Transcription system (Promega, WI, USA). SYBR green-based qRT-PCR was performed using the FastStart Essential DNA Green Master mix (Roche, Switzerland) using the LightCycler 96 system (Roche) to examine the expression levels of target and internal reference genes. Each qRT-PCR reaction was performed with 2 μl of first-strand cDNA and 100 nM each of forward and reverse primers in a total reaction volume of 20 μl. Detailed primer information is listed in Table S1. At least three independent biological and technical repeats were performed for each sample. The 18Sribosomal RNA (rRNA) gene was used as an internal control for normalization^16^. Relative gene expression levels were calculated by the comparative threshold cycle method using the LightCycler 96 qualitative detection module (Roche)^64^.

## Electronic supplementary material

Supplementary information
