# Supplementary material for: Comparative transcriptome analysis of dikaryotic mycelia and mature fruiting bodies in the edible mushroom Lentinula edodes
Source: Sci Rep. 2018 Jun 12;8:8983. doi: 10.1038/s41598-018-27318-z (PMC5997629; doi:10.1038/s41598-018-27318-z)
Supplement: Supplementary file 1 — Supplementary information [file 41598_2018_27318_MOESM1_ESM.docx]

**Supplementary Information**

**Comparative transcriptome analysis of dikaryotic mycelia and mature fruiting bodies in the edible mushroom *Lentinula edodes***

Ha-Yeon Song, Dae-Hyuk Kim, Jung-Mi Kim


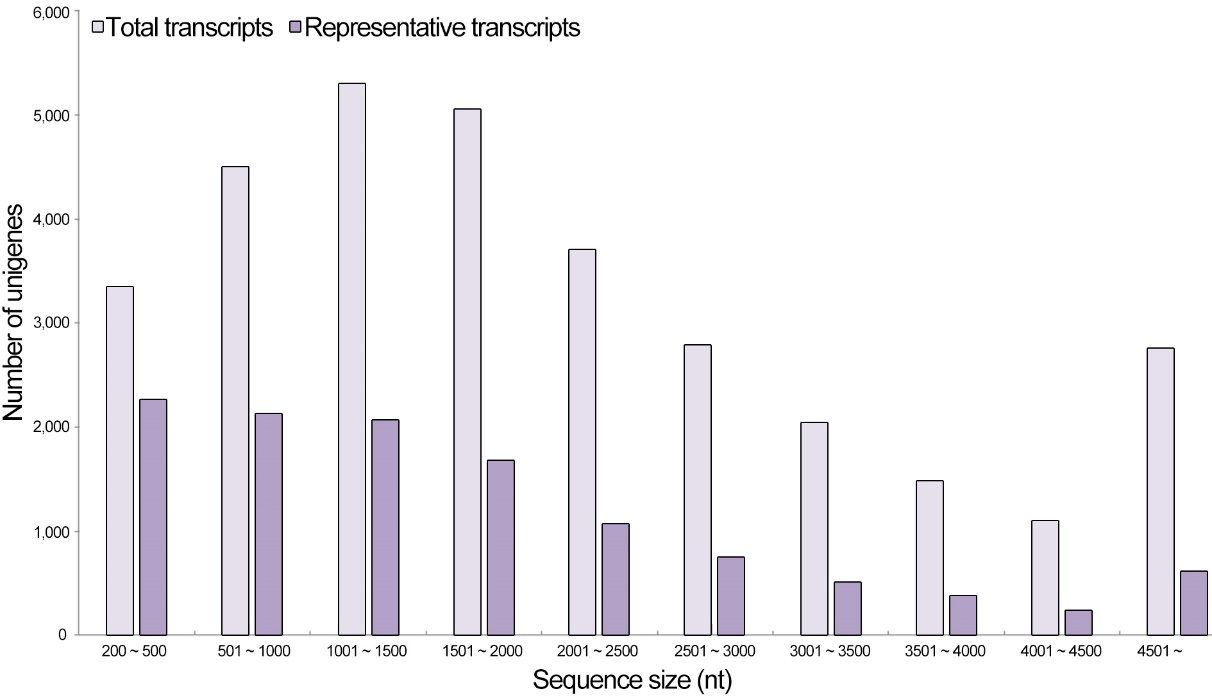


**Figure S1. Length distribution of assembled transcripts from the transcriptomes of the mycelium and fruiting body of *L*. *edodes*.** The horizontal axis shows the length (base pairs) of transcripts and representative transcripts, and the vertical axis shows the number of transcripts and representative transcripts.


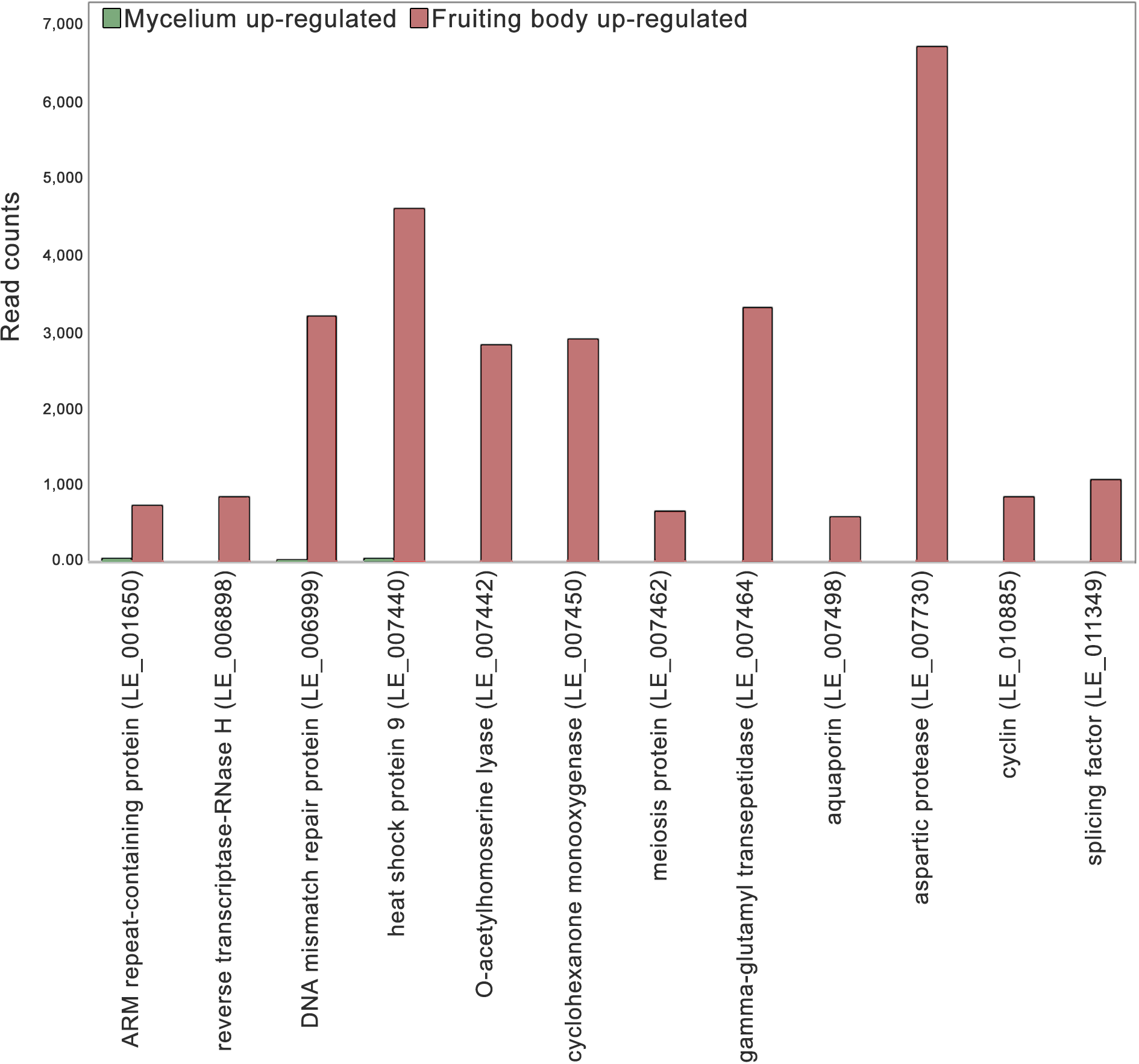


**Figure S2. Comparison of the expression levels of 12 fruiting body-specific genes between mycelium and fruiting body in *L. edodes*.** The red and green bars represent the up- and down-regulated DEGs of the mature fruiting body in *L. edodes*, respectively.

Table S1. Summary of annotations of assembled *L. edodes* sequences.

| Annotated unigenes | NR fungi^a^ | Uniprotkb fungi | KOG^b^ | GO^c^ | KEGG^d^ | InterProscan |
| --- | --- | --- | --- | --- | --- | --- |
| 9,092 | 8,980 | 8,189 | 4,904 | 5,834 | 1,717 | 1,231 |
| 100.0% | 98.8% | 90.1% | 53.9% | 64.2% | 18.9% | 13.5% |

^a^NCBI non-redundant.

^b^Eukaryotic orthologous groups.

^c^Gene ontology.

^d^Kyoto encyclopedia of genes and genome

Table S2. Species distribution of BLASTX data.

| Annotation Species | Matching genes | Percentage of similar bases in match |
| --- | --- | --- |
| *Gymnopus luxurians FD-317 M1* | 5,306 | 59.09% |
| *Termitomyces sp. J132* | 577 | 6.43% |
| *Cylindrobasidium torrendii* | 359 | 4.00% |
| *Lentinula edodes* | 233 | 2.59% |
| *Coprinopsis cenereal* | 223 | 2.48% |
| *Laccaria bicolor* | 217 | 2.42% |
| *Gloeophyllum trabeum* | 192 | 2.14% |
| *Stereum hirsutum* | 178 | 1.98% |
| *Punctularia strigosozonata* | 114 | 1.27% |
| *Fistulina hepatica* | 112 | 1.25% |
| ETC. (species: 191) | 1469 | 16.36% |
| Total | 8,980 | 100.00% |

Table S3. GO functional classification of the *L. edodes* transcriptome.

| Category | GO ID | GO Term | Num. of assembled transcripts |
| --- | --- | --- | --- |
| Biological process | GO:0000003 | Reproduction | 156 |
|  | GO:0008152 | Metabolic process | 2,415 |
|  | GO:0009987 | Cellular process | 2,698 |
|  | GO:0040011 | Locomotion | 21 |
|  | GO:0048511 | Rhythmic process | 4 |
|  | GO:0050896 | Response to stimulus | 602 |
| Biological process total |  |  | 5,896 |
| Cellular component | GO:0005576 | Extracellular region | 44 |
|  | GO:0005623 | Cell | 2,479 |
|  | GO:0009295 | Nucleoid | 7 |
|  | GO:0016020 | Membrane | 749 |
|  | GO:0030054 | Cell junction | 11 |
|  | GO:0045202 | Synapse | 1 |
| Cellular component total |  |  | 3,291 |
| Molecular function | GO:0003824 | Catalytic activity | 2,004 |
|  | GO:0005198 | Structural molecule activity | 108 |
|  | GO:0005215 | Transporter activity | 320 |
|  | GO:0009055 | Electron carrier activity | 10 |
|  | GO:0016209 | Antioxidant activity | 18 |
|  | GO:0045182 | Translation regulator activity | 2 |
| Molecular function total |  |  | 2,462 |
| GO total |  |  | 11,649 |

Table S4. KOG functional categories of *L. edodes* unigenes.

| Category | Description | Num. of assembled transcripts | |
| --- | --- | --- | --- |
| A | RNA processing and modification | 211 |  |
| B | Chromatin structure and dynamics | 88 |  |
| C | Energy production and conversion | 275 |  |
| D | Cell cycle control, cell division, chromosome partitioning | 170 |  |
| E | Amino acid transport and metabolism | 228 |  |
| F | Nucleotide transport and metabolism | 68 |  |
| G | Carbohydrate transport and metabolism | 281 |  |
| H | Coenzyme transport and metabolism | 88 |  |
| I | Lipid transport and metabolism | 251 |  |
| J | Translation, ribosomal structure and biogenesis | 292 |  |
| K | Transcription | 233 |  |
| L | Replication, recombination and repair | 194 |  |
| M | Cell wall/membrane/envelope biogenesis | 69 |  |
| N | Cell motility | 4 |  |
| O | Posttranslational modification, protein turnover, chaperones | 429 |  |
| P | Inorganic ion transport and metabolism | 97 |  |
| Q | Secondary metabolites biosynthesis, transport and catabolism | 299 |  |
| R | General function prediction only | 788 |  |
| S | Function unknown | 264 |  |
| T | Signal transduction mechanisms | 358 |  |
| U | Intracellular trafficking, secretion, and vesicular transport | 243 |  |
| V | Defense mechanisms | 37 |  |
| W | Extracellular structures | 5 |  |
| X | Neuronal membrane glycol-/myelin proteolipid-/unnamed protein | 292 |  |
| Y | Nuclear structure | 28 |  |
| Z | Cytoskeleton | 100 |  |
| - | Not classified | 147 |  |
| KOG total | | 5,539 |  |

Table S5. KEGG pathway functional classification of *L. edodes* unigenes.

| Major classification | Sub classification | Pathway ID | Pathway name | Num. of assembled transcripts |
| --- | --- | --- | --- | --- |
| Cellular processes | Cell growth and death | lbc04111 | Cell cycle | 56 |
|  |  | lbc04113 | Meiosis | 5 |
|  | Cell growth and death total |  |  | 61 |
|  | Transport and catabolism | lbc04144 | Endocytosis | 49 |
|  |  | lbc04146 | Peroxisome | 26 |
|  |  | lbc04145 | Phagosome | 11 |
|  |  | lbc04140 | Regulation of autophagy | 9 |
|  |  | lbc04139 | Regulation of mitophagy | 10 |
|  | Transport and catabolism total | |  | 105 |
| Cellular process total | | | | 166 |
| Environmental information processing | Membrane transport | lbc02010 | ABC transporters | 8 |
|  | Membrane transport total |  |  | 8 |
|  | Signal transduction | lbc04011 | MAPK signaling pathway | 75 |
|  |  | lbc04070 | Phosphatidylinositol signaling system | 2 |
|  | Signal transduction total |  |  | 77 |
| Environmental information processing total | | | | 85 |
| Genetic information processing | Folding, sorting and degradation | lbc03050 | Proteasome | 28 |
|  |  | lbc03060 | Protein export | 16 |
|  |  | lbc04141 | Protein processing in endoplasmic reticulum | 38 |
|  |  | lbc03018 | RNA degradation | 34 |
|  |  | lbc04130 | SNARE interactions in vesicular transport | 13 |
|  |  | lbc04122 | Sulfur relay system | 6 |
|  |  | lbc04120 | Ubiquitin mediated proteolysis | 27 |
|  | Folding, sorting and degradation total |  |  | 162 |
|  | Replication and repair | lbc03410 | Base excision repair | 15 |
|  |  | lbc03030 | DNA replication | 20 |
|  |  | lbc03440 | Homologous recombination | 10 |
|  |  | lbc03430 | Mismatch repair | 7 |
|  |  | lbc03450 | Non-homologous end-joining | 5 |
|  |  | lbc03420 | Nucleotide excision repair | 10 |
|  | Replication and repair total | | | 67 |
|  | Translation | lbc00970 | Aminoacyl-tRNA biosynthesis | 33 |
|  |  | lbc03015 | mRNA surveillance pathway | 30 |
|  |  | lbc03010 | Ribosome | 84 |
|  |  | lbc03008 | Ribosome biogenesis in eukaryotes | 55 |
|  |  | lbc03013 | RNA transport | 73 |
|  | Translation total |  |  | 275 |
|  | Transcription | lbc03022 | Basal transcription factors | 21 |
|  |  | lbc03040 | Spliceosome | 61 |
|  | Transcription total |  |  | 82 |
| Genetic information processing total | | | | 586 |
| Metabolism | Amino acid metabolism | lbc00250 | Alanine, aspartate and glutamate metabolism | 12 |
|  |  | lbc00330 | Arginine and proline metabolism | 14 |
|  |  | lbc00220 | Arginine biosynthesis | 15 |
|  |  | lbc00270 | Cysteine and methionine metabolism | 23 |
|  |  | lbc00260 | Glycine, serine and threonine metabolism | 31 |
|  |  | lbc00340 | Histidine metabolism | 13 |
|  |  | lbc00300 | Lysine biosynthesis | 11 |
|  |  | lbc00310 | Lysine degradation | 7 |
|  |  | lbc00360 | Phenylalanine metabolism | 4 |
|  |  | lbc00400 | Phenylalanine, tyrosine and tryptophan biosynthesis | 13 |
|  |  | lbc00380 | Tryptophan metabolism | 8 |
|  |  | lbc00350 | Tyrosine metabolism | 6 |
|  |  | lbc00290 | Valine, leucine and isoleucine biosynthesis | 9 |
|  |  | lbc00280 | Valine, leucine and isoleucine degradation | 14 |
|  | Amino acid metabolism total | |  | 180 |
|  | Biosynthesis of other secondary metabolites | lbc01100 | Metabolic pathways | 20 |
|  | Biosynthesis of other secondary metabolites total | | | 20 |
|  | Carbohydrate metabolism | lbc00520 | Amino sugar and nucleotide sugar metabolism | 19 |
|  |  | lbc00053 | Ascorbate and aldarate metabolism | 2 |
|  |  | lbc00020 | Citrate cycle (TCA cycle) | 24 |
|  |  | lbc00051 | Fructose and mannose metabolism | 9 |
|  |  | lbc00052 | Galactose metabolism | 7 |
|  |  | lbc00010 | Glycolysis / Gluconeogenesis | 46 |
|  |  | lbc00630 | Glyoxylate and dicarboxylate metabolism | 3 |
|  |  | lbc00562 | Inositol phosphate metabolism | 21 |
|  |  | lbc00040 | Pentose and glucuronate interconversions | 23 |
|  |  | lbc00030 | Pentose phosphate pathway | 13 |
|  |  | lbc00640 | Propanoate metabolism | 1 |
|  |  | lbc00620 | Pyruvate metabolism | 18 |
|  |  | lbc00500 | Starch and sucrose metabolism | 29 |
|  | Carbohydrate metabolism total | | | 215 |
|  | Energy metabolism | lbc00680 | Methane metabolism | 6 |
|  |  | lbc00910 | Nitrogen metabolism | 6 |
|  |  | lbc00190 | Oxidative phosphorylation | 50 |
|  |  | lbc00920 | Sulfur metabolism | 9 |
|  | Energy metabolism total | | | 71 |
|  | Glycan biosynthesis and metabolism | lbc00563 | Glycosylphosphatidylinositol(GPI)-anchor biosynthesis | 15 |
|  |  | lbc00510 | N-Glycan biosynthesis | 26 |
|  |  | lbc00511 | Other glycan degradation | 9 |
|  |  | lbc00514 | Other types of O-glycan biosynthesis | 14 |
|  | Glycan biosynthesis and metabolism total | | | 64 |
|  | Lipid metabolism | lbc00590 | Arachidonic acid metabolism | 4 |
|  |  | lbc01040 | Biosynthesis of unsaturated fatty acids | 9 |
|  |  | lbc00565 | Ether lipid metabolism | 1 |
|  |  | lbc00061 | Fatty acid biosynthesis | 12 |
|  |  | lbc00071 | Fatty acid degradation | 7 |
|  |  | lbc00062 | Fatty acid elongation | 4 |
|  |  | lbc00561 | Glycerolipid metabolism | 15 |
|  |  | lbc00564 | Glycerophospholipid metabolism | 20 |
|  |  | lbc00600 | Sphingolipid metabolism | 16 |
|  |  | lbc00100 | Steroid biosynthesis | 14 |
|  |  | lbc00072 | Synthesis and degradation of ketone bodies | 3 |
|  | Lipid metabolism total | | | 108 |
|  | Metabolism of cofactors and vitamins | lbc00780 | Biotin metabolism | 5 |
|  |  | lbc00790 | Folate biosynthesis | 7 |
|  |  | lbc00785 | Lipoic acid metabolism | 3 |
|  |  | lbc00760 | Nicotinate and nicotinamide metabolism | 13 |
|  |  | lbc00670 | One carbon pool by folate | 6 |
|  |  | lbc00770 | Pantothenate and CoA biosynthesis | 7 |
|  |  | lbc00860 | Porphyrin and chlorophyll metabolism | 12 |
|  |  | lbc00740 | Riboflavin metabolism | 6 |
|  |  | lbc00730 | Thiamine metabolism | 5 |
|  |  | lbc00130 | Ubiquinone and other terpenoid-quinone biosynthesis | 11 |
|  |  | lbc00750 | Vitamin B6 metabolism | 1 |
|  | Metabolism of cofactors and vitamins total | | | 76 |
|  | Metabolism of other amino acids | lbc00460 | Cyanoamino acid metabolism | 13 |
|  |  | lbc00480 | Glutathione metabolism | 15 |
|  |  | lbc00450 | Selenocompound metabolism | 2 |
|  |  | lbc00430 | Taurine and hypotaurine metabolism | 9 |
|  | Metabolism of other amino acids total | | | 39 |
|  | Metabolism of terpenoids and polyketides | lbc00900 | Terpenoid backbone biosynthesis | 13 |
|  | Metabolism of terpenoids and polyketides total | | | 13 |
|  | Nucleotide metabolism | lbc00230 | Purine metabolism | 73 |
|  |  | lbc00240 | Pyrimidine metabolism | 16 |
|  | Nucleotide metabolism total | | | 89 |
| Metabolism total | | | | 875 |
| KEGG total | | | | 1,712 |

Table S6. GO enrichment analysis of DEGs.

| Category | GO term | Fruiting body up: Gene Count | Mycelium up: Gene Count |
| --- | --- | --- | --- |
| Biological process | Aging | 0 | 1 |
|  | Ascospore formation | 1 | 0 |
|  | Carbohydrate metabolic process | 18 | 76 |
|  | Cellular aldehyde metabolic process | 2 | 0 |
|  | Cellular amino acid metabolic process | 0 | 46 |
|  | Cellular aromatic compound metabolic process | 0 | 90 |
|  | Cellular carbohydrate metabolic process | 10 | 34 |
|  | Cell cycle | 0 | 17 |
|  | Cell division | 0 | 5 |
|  | Cell wall organization | 4 | 8 |
|  | Chromosome segregation | 6 | 3 |
|  | Conidium formation | 0 | 4 |
|  | Conjugation with cellular fusion | 0 | 7 |
|  | DNA repair | 6 | 3 |
|  | Establishment or maintenance of cell polarity | 0 | 3 |
|  | Filamentous growth of a population of unicellular Organisms | 1 | 0 |
|  | Fungal-type cell wall organization or biogenesis | 0 | 9 |
|  | Gene silencing | 2 | 0 |
|  | Germ cell development | 1 | 0 |
|  | Heart development | 0 | 1 |
|  | Interaction with host | 0 | 3 |
|  | Lipid metabolic process | 11 | 31 |
|  | Microtubule-based process | 5 | 6 |
|  | Multicellular organismal development | 0 | 19 |
|  | Oxidation-reduction process | 3 | 7 |
|  | Phenylpropanoid metabolic process | 0 | 2 |
|  | Protein glycosylation | 0 | 3 |
|  | Protein metabolic process | 0 | 120 |
|  | Reciprocal meiotic recombination | 1 | 0 |
|  | Response to heat | 0 | 1 |
|  | Response to osmotic stress | 0 | 2 |
|  | Response to oxidative stress | 3 | 0 |
|  | Response to starvation | 3 | 0 |
|  | Response to temperature stimulus | 0 | 3 |
|  | Secondary metabolic process | 4 | 12 |
|  | Small molecule metabolic process | 0 | 111 |
|  | Sporulation resulting in formation of a cellular spore | 2 | 3 |
|  | Translational initiation | 1 | 3 |
|  | Transmembrane transport | 9 | 22 |
|  | Transport | 23 | 80 |
| Biological process total | | 116 | 735 |
| Cellular component | Bounding membrane of organelle | 0 | 14 |
|  | Cell tip | 0 | 2 |
|  | Cell wall | 2 | 10 |
|  | Cellular bud neck | 0 | 1 |
|  | Cytoplasm | 54 | 185 |
|  | Cytoplasmic side of plasma membrane | 1 | 0 |
|  | Integral component of membrane | 11 | 18 |
|  | Intracellular membrane-bounded organelle | 77 | 171 |
|  | Membrane-bounded vesicle | 5 | 0 |
|  | Microtubule associated complex | 2 | 1 |
|  | Mitochondrial membrane | 4 | 0 |
|  | Nuclear chromatin | 2 | 2 |
|  | Proteasome complex | 1 | 1 |
|  | Ribosome | 0 | 9 |
|  | Spliceosomal complex | 1 | 0 |
|  | Transcription factor complex | 0 | 2 |
|  | Vacuolar membrane | 5 | 7 |
| Cellular component total | | 165 | 423 |
| Molecular Function | Cation binding | 22 | 51 |
|  | Coenzyme binding | 0 | 20 |
|  | Cytoskeletal protein binding | 0 | 3 |
|  | Dioxygenase activity | 1 | 0 |
|  | Enzyme activator activity | 0 | 2 |
|  | Hydrolase activity, acting on acid anhydrides | 18 | 0 |
|  | Hydrolase activity, acting on carbon-nitrogen bonds | 3 | 12 |
|  | Hydrolase activity, acting on ester bonds | 0 | 19 |
|  | Hydrolase activity, acting on glycosyl bonds | 9 | 47 |
|  | Identical protein binding | 0 | 2 |
|  | Iron-sulfur cluster binding | 0 | 4 |
|  | Monooxygenase activity | 1 | 6 |
|  | Nucleic acid binding | 28 | 34 |
|  | Nucleotide binding | 23 | 72 |
|  | Oxidoreductase activity, acting on CH-OH group | 0 | 17 |
|  | Oxidoreductase activity, acting on paired donors, with incorporation or reduction of molecular oxygen | 1 | 6 |
|  | Oxidoreductase activity, acting on the CH-CH group | 0 | 3 |
|  | Peptidase activity | 0 | 35 |
|  | Phospholipid binding | 1 | 0 |
|  | Protein complex binding | 0 | 4 |
|  | Protein dimerization activity | 0 | 3 |
|  | Protein transporter activity | 0 | 2 |
|  | Receptor binding | 0 | 4 |
|  | Sequence-specific DNA binding RNA polymerase II transcription factor activity | 2 | 3 |
|  | Serine hydrolase activity | 0 | 8 |
|  | Substrate-specific transmembrane transporter activity | 10 | 35 |
|  | Transferase activity, transferring acyl groups | 6 | 19 |
|  | Transferase activity, transferring glycosyl groups | 1 | 0 |
|  | Transferase activity, transferring phosphorus-containing groups | 0 | 28 |
| Molecular function | | 126 | 439 |
| GO total | | 407 | 1,597 |

Table S7. KEGG pathway enrichment analysis of DEGs.

| Major Classification | Sub Classification | Pathway name | Fruiting body up: Gene Count | Mycelium up: Gene Count |
| --- | --- | --- | --- | --- |
| Cellular processes | Cell growth and death | Cell cycle | 4 | 4 |
|  |  | Meiosis | 0 | 1 |
|  | Cell growth and death total |  | 4 | 5 |
|  | Transport and catabolism | Regulation of autophagy | 0 | 1 |
|  |  | Phagosome | 0 | 4 |
|  |  | Peroxisome | 1 | 3 |
|  | Transport and catabolism total |  | 1 | 8 |
| Cellular processes total |  |  | 5 | 13 |
| Environmental information processing | Membrane transport | ABC transporters | 0 | 2 |
|  | Membrane transport total |  | 0 | 2 |
|  | Signal transduction | MAPK signaling pathway | 1 | 10 |
|  | Signal transduction total |  | 1 | 12 |
| Environmental information processing total | | | 1 | 14 |
| Genetic information processing | Folding, sorting and degradation | RNA degradation | 2 | 2 |
|  |  | Proteasome | 1 | 3 |
|  |  | Protein export | 0 | 2 |
|  |  | Ubiquitin mediated proteolysis | 0 | 3 |
|  |  | Sulfur relay system | 0 | 1 |
|  |  | Protein processing in endoplasmic reticulum | 1 | 5 |
|  | Folding, sorting and degradation total | | 4 | 16 |
|  | Replication and repair | DNA replication | 1 | 1 |
|  |  | Base excision repair | 0 | 1 |
|  |  | Mismatch repair | 2 | 1 |
|  |  | Homologous recombination | 1 | 0 |
|  |  | Non-homologous end-joining | 1 | 0 |
|  | Replication and repair total |  | 5 | 3 |
|  | Transcription | Basal transcription factors | 1 | 1 |
|  |  | Spliceosome | 4 | 2 |
|  | Transcription total |  | 5 | 3 |
|  | Translation | Aminoacyl-tRNA biosynthesis | 1 | 14 |
|  |  | Ribosome biogenesis in eukaryotes | 2 | 3 |
|  |  | Ribosome | 0 | 8 |
|  |  | RNA transport | 3 | 7 |
|  |  | mRNA surveillance pathway | 1 | 2 |
|  | Translation total |  | 7 | 34 |
| Genetic information processing total | | | 21 | 56 |
| Metabolism | Amino acid metabolism | Arginine biosynthesis | 1 | 1 |
|  |  | Alanine, aspartate and glutamate metabolism | 0 | 4 |
|  |  | Glycine, serine and threonine metabolism | 2 | 11 |
|  |  | Cysteine and methionine metabolism | 1 | 9 |
|  |  | Valine, leucine and isoleucine degradation | 2 | 2 |
|  |  | Valine, leucine and isoleucine biosynthesis | 1 | 5 |
|  |  | Lysine biosynthesis | 0 | 5 |
|  |  | Lysine degradation | 2 | 0 |
|  |  | Arginine and proline metabolism | 1 | 7 |
|  |  | Histidine metabolism | 1 | 3 |
|  |  | Tyrosine metabolism | 0 | 2 |
|  |  | Phenylalanine metabolism | 0 | 1 |
|  |  | Tryptophan metabolism | 1 | 2 |
|  |  | Phenylalanine, tyrosine and tryptophan biosynthesis | 2 | 3 |
|  | Amino acid metabolism total |  | 14 | 55 |
|  | Biosynthesis of other secondary metabolites | Metabolic pathways | 2 | 3 |
|  | Biosynthesis of other secondary metabolites total | | 2 | 3 |
|  | Carbohydrate metabolism | Glycolysis / Gluconeogenesis | 7 | 9 |
|  |  | Citrate cycle (TCA cycle) | 1 | 5 |
|  |  | Pentose phosphate pathway | 0 | 5 |
|  |  | Pentose and glucuronate interconversions | 2 | 10 |
|  |  | Fructose and mannose metabolism | 0 | 4 |
|  |  | Galactose metabolism | 0 | 4 |
|  |  | Ascorbate and aldarate metabolism | 1 | 1 |
|  |  | Starch and sucrose metabolism | 3 | 12 |
|  |  | Amino sugar and nucleotide sugar metabolism | 1 | 3 |
|  |  | Inositol phosphate metabolism | 1 | 3 |
|  |  | Pyruvate metabolism | 0 | 4 |
|  |  | Glyoxylate and dicarboxylate metabolism | 0 | 1 |
|  |  | Propanoate metabolism | 0 | 1 |
|  | Carbohydrate metabolism total | | 16 | 62 |
|  | Energy metabolism | Oxidative phosphorylation | 0 | 1 |
|  |  | Methane metabolism | 2 | 2 |
|  |  | Nitrogen metabolism | 0 | 2 |
|  |  | Sulfur metabolism | 0 | 4 |
|  | Energy metabolism total |  | 2 | 9 |
|  | Glycan biosynthesis and metabolism | N-Glycan biosynthesis | 0 | 4 |
|  |  | Other glycan degradation | 1 | 3 |
|  |  | Other types of O-glycan biosynthesis | 0 | 2 |
|  |  | Glycosylphosphatidylinositol(GPI)-anchor biosynthesis | 1 | 3 |
|  | Glycan biosynthesis and metabolism total | | 2 | 12 |
|  | Lipid metabolism | Fatty acid biosynthesis | 0 | 1 |
|  |  | Fatty acid degradation | 0 | 3 |
|  |  | Synthesis and degradation of ketone bodies | 0 | 2 |
|  |  | Steroid biosynthesis | 1 | 4 |
|  |  | Glycerolipid metabolism | 1 | 2 |
|  |  | Glycerophospholipid metabolism | 1 | 3 |
|  |  | Arachidonic acid metabolism | 0 | 1 |
|  |  | Sphingolipid metabolism | 0 | 2 |
|  |  | Biosynthesis of unsaturated fatty acids | 0 | 3 |
|  | Lipid metabolism total |  | 3 | 21 |
|  | Metabolism of cofactors and vitamins | Ubiquinone and other terpenoid-quinone biosynthesis | 0 | 4 |
|  |  | One carbon pool by folate | 1 | 1 |
|  |  | Thiamine metabolism | 1 | 1 |
|  |  | Vitamin B6 metabolism | 0 | 1 |
|  |  | Nicotinate and nicotinamide metabolism | 1 | 0 |
|  |  | Pantothenate and CoA biosynthesis | 0 | 2 |
|  |  | Biotin metabolism | 0 | 3 |
|  |  | Lipoic acid metabolism | 0 | 1 |
|  |  | Folate biosynthesis | 1 | 3 |
|  | Metabolism of cofactors and vitamins total | | 4 | 16 |
|  | Metabolism of other amino acids | Taurine and hypotaurine metabolism | 1 | 2 |
|  |  | Selenocompound metabolism | 0 | 1 |
|  |  | Cyanoamino acid metabolism | 0 | 7 |
|  |  | Glutathione metabolism | 0 | 3 |
|  | Metabolism of other amino acids total | | 1 | 13 |
|  | Metabolism of terpenoids and polyketides | Terpenoid backbone biosynthesis | 1 | 2 |
|  | Metabolism of terpenoids and polketides total | | 1 | 2 |
|  | Nucleotide metabolism | Purine metabolism | 1 | 12 |
|  |  | Pyrimidine metabolism | 1 | 4 |
|  | Nucleotide metabolism total |  | 2 | 16 |
| Metabolism total |  |  | 47 | 209 |
| KEGG total |  |  | 74 | 290 |

Table S8. DEGs between the mycelium and mature fruiting body of *L. edodes.*

| Unigene ID | Mycelium read count | Fruiting body read count | Log2foldchange | Fruiting body *vs* Mycelium | Description | Species^a^ | Evalue | Identity |
| --- | --- | --- | --- | --- | --- | --- | --- | --- |
| LE_007429 | 23 | 77,472 | 11.72 | up |  |  |  |  |
| LE_007412 | 5 | 8,057 | 10.60 | up |  | *L. edodes* | 2E-34 | 99.21 |
| LE_007861 | 4 | 3,037 | 9.49 | up | hypothetical protein GYMLUDRAFT_46377 | *G. luxurians* FD-317 M1 | 1E-40 | 60.16 |
| LE_011335 | 4 | 2,723 | 9.32 | up | hypothetical protein GYMLUDRAFT_486395 | *G. luxurians* FD-317 M1 | 0 | 68.95 |
| LE_007459 | 2 | 1,138 | 9.26 | up | glycoside hydrolase family 61 protein | *G. luxurians* FD-317 M1 | 2E-54 | 63.87 |
| LE_007730 | 11 | 6,695 | 9.23 | up | aspartic protease | *L. edodes* | 0 | 98.78 |
| LE_007727 | 3 | 1,853 | 9.08 | up | hypothetical protein GYMLUDRAFT_73314 | *G. luxurians* FD-317 M1 | 4E-125 | 52.70 |
| LE_007912 | 3 | 1,612 | 9.01 | up |  |  |  |  |
| LE_007951 | 2 | 894 | 8.73 | up |  |  |  |  |
| LE_006927 | 15 | 6,593 | 8.73 | up | zinc induced facilitator 1 | *Termitomyces sp.* J132 | 1E-158 | 46.08 |
| LE_007498 | 1 | 587 | 8.55 | up | hypothetical protein GYMLUDRAFT_243493 | *G. luxurians* FD-317 M1 | 3E-107 | 58.22 |
| LE_007456 | 1 | 531 | 8.41 | up | zf-MYND domain-containing protein | *L. edodes* | 9E-32 | 53.73 |
| LE_007450 | 9 | 2,890 | 8.34 | up | hypothetical protein GYMLUDRAFT_41664 | *G. luxurians* FD-317 M1 | 0 | 66.42 |
| LE_006542 | 7 | 2,330 | 8.26 | up | kinase | *F. hepatica* ATCC 64428 | 9E-67 | 39.38 |
| LE_006156 | 7 | 1,962 | 8.13 | up | GPI-anchored small secreted protein | *L. bicolor* S238N-H82 | 1E-20 | 32.45 |
| LE_007448 | 4 | 1,253 | 8.11 | up | cytochrome P450 | *L. edodes* | 0 | 100.00 |
| LE_011338 | 1 | 359 | 7.84 | up | hypothetical protein GYMLUDRAFT_214163 | *G. luxurians* FD-317 M1 | 3E-100 | 71.03 |
| LE_007442 | 12 | 2,826 | 7.83 | up | O-acetylhomoserine (thiol)-lyase | *R. solani* AG-1 IB | 6E-180 | 64.80 |
| LE_007695 | 7 | 1,586 | 7.70 | up | hypothetical protein GYMLUDRAFT_944329 | *G. luxurians* FD-317 M1 | 3E-22 | 63.77 |
| LE_009311 | 35 | 7,282 | 7.67 | up | hypothetical protein GYMLUDRAFT_39929 | *G. luxurians* FD-317 M1 | 2E-47 | 43.02 |
| LE_005239 | 7 | 1,240 | 7.33 | up | hypothetical protein GYMLUDRAFT_39615 | *G. luxurians* FD-317 M1 | 3E-120 | 69.20 |
| LE_007328 | 5 | 901 | 7.23 | up | hypothetical protein GYMLUDRAFT_172776 | *G. luxurians* FD-317 M1 | 1E-39 | 80.21 |
| LE_007467 | 9 | 1,385 | 7.22 | up | hypothetical protein GYMLUDRAFT_89343 | *G. luxurians* FD-317 M1 | 4E-80 | 60.74 |
| LE_007492 | 2 | 349 | 7.11 | up | hypothetical protein GYMLUDRAFT_262386 | *G. luxurians* FD-317 M1 | 2E-22 | 33.21 |
| LE_007464 | 24 | 3,310 | 7.10 | up | gamma-glutamyl transpeptidase | *L. edodes* | 0 | 91.85 |
| LE_007775 | 3 | 358 | 7.02 | up | copper radical oxidase | *T. cinnabarina* | 0 | 62.42 |
| LE_007415 | 1 | 185 | 6.89 | up | hypothetical protein GYMLUDRAFT_54114 | *G. luxurians* FD-317 M1 | 2E-77 | 48.43 |
| LE_007509 | 2 | 326 | 6.89 | up | hypothetical protein GYMLUDRAFT_156339 | *G. luxurians* FD-317 M1 | 9E-25 | 84.75 |
| LE_003780 | 60 | 7179 | 6.88 | up |  |  |  |  |
| LE_007447 | 3 | 393 | 6.87 | up | hypothetical protein GYMLUDRAFT_214141 | *G. luxurians* FD-317 M1 | 0 | 70.53 |
| LE_007331 | 2 | 295 | 6.87 | up | hypothetical protein GYMLUDRAFT_252319 | *G. luxurians* FD-317 M1 | 0 | 60.04 |
| LE_007754 | 4 | 472 | 6.83 | up | hypothetical protein GYMLUDRAFT_65144 | *G. luxurians* FD-317 M1 | 2E-83 | 46.49 |
| LE_010986 | 4 | 480 | 6.81 | up | similar to oxidase | *B. cinerea* T4 | 8E-141 | 59.21 |
| LE_006999 | 28 | 3,192 | 6.80 | up | hypothetical protein GYMLUDRAFT_67285 | *G. luxurians* FD-317 M1 | 0 | 63.92 |
| LE_006715 | 15 | 1,726 | 6.79 | up | hypothetical protein GYMLUDRAFT_237223 | *G. luxurians* FD-317 M1 | 1E-107 | 82.27 |
| LE_007460 | 3 | 406 | 6.75 | up | glycoside hydrolase family 17 protein | *G. luxurians* FD-317 M1 | 4E-180 | 82.06 |
| LE_011394 | 5 | 525 | 6.72 | up | hypothetical protein GYMLUDRAFT_216491 | *G. luxurians* FD-317 M1 | 0 | 77.05 |
| LE_011135 | 2 | 279 | 6.66 | up |  |  |  |  |
| LE_007440 | 47 | 4,597 | 6.61 | up | hypothetical protein CYLTODRAFT_344835 | *C. torrendii* FP15055 ss-10 | 5E-21 | 68.29 |
| LE_011349 | 11 | 1,085 | 6.59 | up | hypothetical protein GYMLUDRAFT_720987 | *G. luxurians* FD-317 M1 | 0 | 73.20 |
| LE_007493 | 2 | 232 | 6.53 | up |  |  |  |  |
| LE_007588 | 2 | 249 | 6.50 | up | glycoside hydrolase | *S. hirsutum* FP-91666 SS1 | 2E-164 | 47.18 |
| LE_007878 | 2 | 194 | 6.49 | up | hypothetical protein GYMLUDRAFT_69285 | *G. luxurians* FD-317 M1 | 0 | 63.22 |
| LE_003624 | 37 | 3,315 | 6.48 | up |  |  |  |  |
| LE_007462 | 7 | 670 | 6.44 | up | hypothetical protein GYMLUDRAFT_37419 | *G. luxurians* FD-317 M1 | 4E-75 | 78.12 |
| LE_007466 | 3 | 287 | 6.41 | up | hypothetical protein GYMLUDRAFT_165430 | *G. luxurians* FD-317 M1 | 0 | 59.48 |
| LE_011392 | 5 | 391 | 6.36 | up | hypothetical protein GYMLUDRAFT_36803 | *G. luxurians* FD-317 M1 | 8E-22 | 46.51 |
| LE_007558 | 1 | 127 | 6.35 | up |  |  |  |  |
| LE_004323 | 2 | 181 | 6.35 | up | hypothetical protein GYMLUDRAFT_85120 | *G. luxurians* FD-317 M1 | 4E-66 | 62.94 |
| LE_007441 | 98 | 7,951 | 6.34 | up |  |  |  |  |
| LE_007451 | 3 | 254 | 6.34 | up | laccase 3 | *L. edodes* | 0 | 99.45 |
| LE_007500 | 4 | 313 | 6.31 | up | hypothetical protein GYMLUDRAFT_150251 | *G. luxurians* FD-317 M1 | 0 | 89.18 |
| LE_006802 | 4 | 349 | 6.29 | up | hypothetical protein GYMLUDRAFT_233586 | *G. luxurians* FD-317 M1 | 1E-96 | 54.30 |
| LE_007444 | 5 | 427 | 6.28 | up | lentiavidin1 | *L. edodes* | 6E-102 | 99.34 |
| LE_005547 | 2 | 165 | 6.26 | up | hypothetical protein GYMLUDRAFT_234144 | *G. luxurians* FD-317 M1 | 3E-155 | 55.69 |
| LE_006790 | 8 | 682 | 6.24 | up | carbohydrate esterase family 4 protein | *G. luxurians* FD-317 M1 | 0 | 82.79 |
| LE_007491 | 2 | 144 | 6.23 | up | hypothetical protein GYMLUDRAFT_259312 | *G. luxurians* FD-317 M1 | 3E-122 | 52.14 |
| LE_006895 | 8 | 604 | 6.22 | up | hypothetical protein GYMLUDRAFT_968946 | *G. luxurians* FD-317 M1 | 2E-131 | 62.56 |
| LE_007719 | 1 | 114 | 6.20 | up | glycoside hydrolase family 16 protein | *G. luxurians* FD-317 M1 | 1E-82 | 49.64 |
| LE_007489 | 19 | 1,396 | 6.19 | up | hypothetical protein PLICRDRAFT_103741 | *Plicaturopsis crispa* FD-325 SS-3 | 2E-93 | 55.29 |
| LE_007499 | 3 | 220 | 6.04 | up | hypothetical protein GYMLUDRAFT_161211 | *G. luxurians* FD-317 M1 | 8E-138 | 66.78 |
| LE_007457 | 4 | 295 | 6.03 | up | nuclease Le3 | *L. edodes* | 0 | 90.55 |
| LE_007384 | 5 | 374 | 6.03 | up | hypothetical protein GYMLUDRAFT_1026591 | *G. luxurians* FD-317 M1 | 8E-99 | 61.84 |
| LE_007386 | 4 | 288 | 6.02 | up | hypothetical protein GYMLUDRAFT_179817 | *G. luxurians* FD-317 M1 | 0 | 71.55 |
| LE_010885 | 13 | 844 | 6.00 | up | hypothetical protein GYMLUDRAFT_177288 | *G. luxurians* FD-317 M1 | 2E-98 | 84.29 |
| LE_007485 | 6 | 366 | 5.94 | up | cytochrome P450 4F5 | *Termitomyces sp.* J132 | 0 | 66.67 |
| LE_007729 | 5 | 345 | 5.94 | up | hypothetical protein GYMLUDRAFT_254404 | *G. luxurians* FD-317 M1 | 0 | 51.18 |
| LE_007746 | 3 | 192 | 5.92 | up | hypothetical protein GYMLUDRAFT_252319 | *G. luxurians* FD-317 M1 | 0 | 62.50 |
| LE_007528 | 3 | 232 | 5.92 | up |  |  |  |  |
| LE_007461 | 10 | 605 | 5.89 | up | hypothetical protein GYMLUDRAFT_156068 | *G. luxurians* FD-317 M1 | 0 | 81.45 |
| LE_007613 | 2 | 135 | 5.89 | up | hypothetical protein GYMLUDRAFT_115182 | *G. luxurians* FD-317 M1 | 2E-43 | 74.49 |
| LE_007396 | 22 | 1,266 | 5.83 | up | hypothetical protein GYMLUDRAFT_234401 | *G. luxurians* FD-317 M1 | 0 | 88.22 |
| LE_006046 | 5 | 292 | 5.80 | up | hypothetical protein GYMLUDRAFT_241073 | *G. luxurians* FD-317 M1 | 4E-84 | 50.68 |
| LE_011325 | 2 | 116 | 5.78 | up | hypothetical protein GYMLUDRAFT_240582 | *G. luxurians* FD-317 M1 | 3E-171 | 77.34 |
| LE_007526 | 2 | 103 | 5.74 | up |  |  |  |  |
| LE_006982 | 40 | 2,113 | 5.70 | up | hypothetical protein GYMLUDRAFT_34484 | *G. luxurians* FD-317 M1 | 0 | 83.16 |
| LE_007326 | 3 | 179 | 5.65 | up | hypothetical protein M413DRAFT_373097 | *H. cylindrosporum* h7 | 2E-62 | 44.67 |
| LE_007527 | 3 | 142 | 5.62 | up | hypothetical protein GYMLUDRAFT_236107 | *G. luxurians* FD-317 M1 | 0 | 69.30 |
| LE_011387 | 5 | 280 | 5.61 | up | hypothetical protein GYMLUDRAFT_39421 | *G. luxurians* FD-317 M1 | 4E-42 | 59.40 |
| LE_011038 | 6 | 302 | 5.60 | up | hypothetical protein GYMLUDRAFT_245173 | *G. luxurians* FD-317 M1 | 1E-168 | 64.11 |
| LE_005252 | 11 | 572 | 5.59 | up | hypothetical protein GYMLUDRAFT_775031 | *G. luxurians* FD-317 M1 | 1E-156 | 57.88 |
| LE_007741 | 8 | 426 | 5.56 | up | hypothetical protein GYMLUDRAFT_787891 | *G. luxurians* FD-317 M1 | 0 | 66.10 |
| LE_006892 | 34 | 1,580 | 5.52 | up |  |  |  |  |
| LE_007439 | 11 | 544 | 5.52 | up |  |  |  |  |
| LE_007446 | 16 | 733 | 5.51 | up |  |  |  |  |
| LE_007254 | 3 | 144 | 5.50 | up | hypothetical protein GYMLUDRAFT_174345 | *G. luxurians* FD-317 M1 | 2E-101 | 90.56 |
| LE_004420 | 16 | 740 | 5.46 | up | hypothetical protein GYMLUDRAFT_232266 | *G. luxurians* FD-317 M1 | 0 | 64.70 |
| LE_007469 | 5 | 226 | 5.44 | up | hypothetical protein GYMLUDRAFT_241421 | *G. luxurians* FD-317 M1 | 6E-28 | 86.42 |
| LE_005107 | 36 | 1,530 | 5.40 | up | hypothetical protein GYMLUDRAFT_98786 | *G. luxurians* FD-317 M1 | 0 | 64.41 |
| LE_003797 | 3 | 120 | 5.31 | up | hypothetical protein GYMLUDRAFT_129670 | *G. luxurians* FD-317 M1 | 2E-43 | 67.52 |
| LE_010769 | 8 | 317 | 5.29 | up | glycoside hydrolase family 16 protein | *G. luxurians* FD-317 M1 | 0 | 76.94 |
| LE_011482 | 5 | 201 | 5.23 | up | hypothetical protein GYMLUDRAFT_954151 | *G. luxurians* FD-317 M1 | 6E-86 | 56.21 |
| LE_006605 | 4 | 149 | 5.19 | up | hypothetical protein GYMLUDRAFT_914343 | *G. luxurians* FD-317 M1 | 5E-24 | 41.34 |
| LE_006898 | 23 | 844 | 5.17 | up | reverse transcriptase-RNase H-integrase | *L. bicolor* S238N-H82 | 0 | 59.78 |
| LE_001138 | 313 | 11,240 | 5.16 | up | hypothetical protein GYMLUDRAFT_173271 | *G. luxurians* FD-317 M1 | 3E-116 | 80.29 |
| LE_007507 | 8 | 293 | 5.15 | up | Cytochrome P450 4F12 | *Termitomyces sp*. J132 | 0 | 63.47 |
| LE_007494 | 10 | 358 | 5.12 | up | hypothetical protein GYMLUDRAFT_973996 | *G. luxurians* FD-317 M1 | 0 | 46.96 |
| LE_005284 | 3 | 119 | 5.12 | up | hypothetical protein GYMLUDRAFT_88462 | *G. luxurians* FD-317 M1 | 4E-30 | 43.24 |
| LE_005246 | 17 | 608 | 5.11 | up | hypothetical protein GYMLUDRAFT_53040 | *G. luxurians* FD-317 M1 | 9E-53 | 49.31 |
| LE_007505 | 3 | 132 | 5.10 | up | hypothetical protein J132_06581 | *Termitomyces sp.* J132 | 1E-63 | 33.44 |
| LE_011524 | 3 | 108 | 5.01 | up | DUF1212-domain-containing protein | *C. torrendii* FP15055 ss-10 | 0 | 82.69 |
| LE_005436 | 15 | 479 | 4.98 | up | mismatched base pair and cruciform DNA recognition protein | *Coniophora puteana* RWD-64-598 SS2 | 2E-21 | 62.67 |
| LE_002264 | 101 | 3,166 | 4.96 | up | laccase 2 | *L. edodes* | 0 | 99.81 |
| LE_007568 | 14 | 421 | 4.91 | up | hypothetical protein GYMLUDRAFT_161095 | *G. luxurians* FD-317 M1 | 0 | 85.02 |
| LE_006616 | 31 | 927 | 4.89 | up | hypothetical protein GYMLUDRAFT_265499 | *G. luxurians* FD-317 M1 | 0 | 66.11 |
| LE_011581 | 3 | 107 | 4.88 | up | hypothetical protein GYMLUDRAFT_246002 | *G. luxurians* FD-317 M1 | 2E-15 | 37.19 |
| LE_007549 | 4 | 122 | 4.88 | up |  |  |  |  |
| LE_011539 | 5 | 167 | 4.88 | up | hypothetical protein GYMLUDRAFT_53621 | *G. luxurians* FD-317 M1 | 2E-118 | 52.42 |
| LE_003828 | 100 | 2,903 | 4.85 | up | hypothetical protein GYMLUDRAFT_45898 | *G. luxurians* FD-317 M1 | 2E-153 | 52.88 |
| LE_007340 | 8 | 231 | 4.82 | up | hypothetical protein GYMLUDRAFT_239634 | *G. luxurians* FD-317 M1 | 3E-134 | 60.34 |
| LE_011108 | 4 | 139 | 4.82 | up | hypothetical protein GYMLUDRAFT_45003 | *G. luxurians* FD-317 M1 | 0 | 69.89 |
| LE_007168 | 4 | 118 | 4.82 | up | hypothetical protein GYMLUDRAFT_45901 | *G. luxurians* FD-317 M1 | 1E-178 | 61.76 |
| LE_007085 | 17 | 469 | 4.78 | up | FAD/NAD(P)-binding domain-containing protein | *P. strigosozonata* HHB-11173 SS5 | 0 | 65.24 |
| LE_006658 | 5 | 145 | 4.78 | up |  |  |  |  |
| LE_006763 | 4 | 117 | 4.76 | up |  |  |  |  |
| LE_006607 | 5 | 145 | 4.69 | up | carbohydrate-binding module family 50 protein | *G. luxurians* FD-317 M1 | 1E-52 | 68.57 |
| LE_011160 | 89 | 2,225 | 4.64 | up |  |  |  |  |
| LE_010856 | 15 | 382 | 4.64 | up | hypothetical protein SERLADRAFT_414900 | *S. lacrymans* S7.9 | 5E-169 | 58.31 |
| LE_011186 | 12 | 305 | 4.59 | up | hypothetical protein GYMLUDRAFT_251172 | *G. luxurians* FD-317 M1 | 0 | 75.70 |
| LE_005483 | 8 | 199 | 4.57 | up | hypothetical protein GYMLUDRAFT_841110 | *G. luxurians* FD-317 M1 | 5E-57 | 45.07 |
| LE_003952 | 579 | 13,596 | 4.55 | up | hypothetical protein GYMLUDRAFT_247116 | *G. luxurians* FD-317 M1 | 0 | 76.87 |
| LE_007914 | 13 | 308 | 4.53 | up | hypothetical protein FOMMEDRAFT_30808 | *Fomitiporia mediterranea* MF3/22 | 2E-119 | 65.41 |
| LE_007536 | 6 | 148 | 4.53 | up |  |  |  |  |
| LE_007733 | 6 | 141 | 4.51 | up | hypothetical protein GYMLUDRAFT_246831 | *G. luxurians* FD-317 M1 | 3E-87 | 47.45 |
| LE_011391 | 5 | 119 | 4.49 | up | hypothetical protein GYMLUDRAFT_177527 | *G. luxurians* FD-317 M1 | 3E-58 | 55.96 |
| LE_006725 | 8 | 185 | 4.47 | up | hypothetical protein GYMLUDRAFT_262886 | *G. luxurians* FD-317 M1 | 0 | 53.51 |
| LE_004646 | 60 | 1,333 | 4.47 | up | hypothetical protein GYMLUDRAFT_45828 | *G. luxurians* FD-317 M1 | 3E-22 | 64.71 |
| LE_004255 | 5 | 128 | 4.42 | up | hypothetical protein GYMLUDRAFT_95107 | *G. luxurians* FD-317 M1 | 5E-109 | 66.67 |
| LE_006893 | 87 | 1,849 | 4.40 | up | hypothetical protein GYMLUDRAFT_236073 | *G. luxurians* FD-317 M1 | 0 | 70.97 |
| LE_006412 | 599 | 12,581 | 4.39 | up | hypothetical protein GYMLUDRAFT_90658 | *G. luxurians* FD-317 M1 | 0 | 86.59 |
| LE_004449 | 16 | 334 | 4.36 | up | hypothetical protein MPER_10842 | *Moniliophthora perniciosa* FA553 | 3E-11 | 63.16 |
| LE_006907 | 7 | 144 | 4.27 | up | hypothetical protein GYMLUDRAFT_33958 | *G. luxurians FD-317 M1* | 7E-155 | 72.73 |
| LE_011001 | 16 | 311 | 4.26 | up | CDF-like metal transporter | *L. bicolor* S238N-H82 | 3E-180 | 68.83 |
| LE_006593 | 120 | 2,288 | 4.25 | up | hypothetical protein GYMLUDRAFT_908855 | *G. luxurians* FD-317 M1 | 0 | 56.55 |
| LE_007465 | 7 | 132 | 4.21 | up | hypothetical protein GYMLUDRAFT_887210 | *G. luxurians* FD-317 M1 | 2E-68 | 43.7 |
| LE_006780 | 123 | 2,256 | 4.20 | up | hypothetical protein GYMLUDRAFT_247328 | *G. luxurians* FD-317 M1 | 3E-25 | 41.24 |
| LE_005864 | 6 | 111 | 4.20 | up | hypothetical protein GYMLUDRAFT_182569 | *G. luxurians* FD-317 M1 | 0 | 76.73 |
| LE_007137 | 6 | 108 | 4.19 | up | glycosyltransferase family 49 protein | *G. luxurians* FD-317 M1 | 0 | 70.47 |
| LE_007349 | 8 | 155 | 4.18 | up | hypothetical protein GYMLUDRAFT_971742 | *G. luxurians* FD-317 M1 | 2E-149 | 50.78 |
| LE_002574 | 7 | 137 | 4.16 | up | MFS transporter | *Marssonina brunnea* | 5E-116 | 44.14 |
| LE_011120 | 10 | 180 | 4.13 | up | hypothetical protein GYMLUDRAFT_49211 | *G. luxurians* FD-317 M1 | 0 | 60.43 |
| LE_006448 | 25 | 428 | 4.08 | up | hypothetical protein GYMLUDRAFT_48469 | *G. luxurians* FD-317 M1 | 3E-112 | 61.22 |
| LE_007152 | 10 | 180 | 4.06 | up | hypothetical protein GYMLUDRAFT_151027 | *G. luxurians* FD-317 M1 | 0 | 76.26 |
| LE_004960 | 170 | 2,821 | 4.05 | up | polyketide synthase | *G. luxurians* FD-317 M1 | 0 | 63.03 |
| LE_006805 | 211 | 3,442 | 4.03 | up | hypothetical protein GYMLUDRAFT_41664 | *G. luxurians* FD-317 M1 | 0 | 72.85 |
| LE_005524 | 31 | 513 | 4.01 | up | hypothetical protein CC1G_05084 | *C. cinerea* okayama7#130 | 6E-68 | 51.38 |
| LE_007540 | 7 | 115 | 4.00 | up | hypothetical protein GYMLUDRAFT_178498 | *G. luxurians* FD-317 M1 | 2E-141 | 46.65 |
| LE_001474 | 22 | 353 | 3.99 | up | glycoside hydrolase family 5 protein | *G. luxurians* FD-317 M1 | 0 | 71.66 |
| LE_006308 | 11 | 185 | 3.99 | up | Cloroperoxidase | *G. lozoyensis* ATCC 20868 | 5E-137 | 53.38 |
| LE_006623 | 16 | 257 | 3.99 | up |  |  |  |  |
| LE_007547 | 6 | 100 | 3.99 | up | hypothetical protein GYMLUDRAFT_43286 | *G. luxurians* FD-317 M1 | 0 | 71.41 |
| LE_011090 | 28 | 441 | 3.96 | up | hypothetical protein GYMLUDRAFT_42096 | *G. luxurians* FD-317 M1 | 8E-58 | 69.93 |
| LE_006722 | 28 | 447 | 3.96 | up | hypothetical protein GYMLUDRAFT_42096 | *G. luxurians* FD-317 M1 | 1E-56 | 69.93 |
| LE_006721 | 29 | 447 | 3.94 | up | hypothetical protein GYMLUDRAFT_42096 | *G. luxurians* FD-317 M1 | 2E-57 | 69.93 |
| LE_010532 | 347 | 5,281 | 3.93 | up | hypothetical protein GYMLUDRAFT_35357 | *G. luxurians* FD-317 M1 | 0 | 86.44 |
| LE_007482 | 16 | 254 | 3.93 | up | hypothetical protein GYMLUDRAFT_241285 | *G. luxurians* FD-317 M1 | 5E-80 | 66.14 |
| LE_001650 | 49 | 746 | 3.92 | up | hypothetical protein AGABI1DRAFT_110884 | *A. bisporus var. burnettii* JB137-S8 | 0 | 88.15 |
| LE_001447 | 131 | 1,950 | 3.90 | up | hypothetical protein GYMLUDRAFT_155698 | *G. luxurians* FD-317 M1 | 0 | 87.41 |
| LE_006781 | 27 | 400 | 3.90 | up | delta-endotoxin *CytB* | *G. trabeum* ATCC 11539 | 6E-88 | 69.95 |
| LE_000097 | 413 | 6,184 | 3.90 | up |  |  |  |  |
| LE_010744 | 40 | 601 | 3.90 | up | transporter MCH4 | *Termitomyces sp.* J132 | 6E-62 | 64.16 |
| LE_007476 | 27 | 413 | 3.90 | up | hypothetical protein GYMLUDRAFT_238767 | *G. luxurians* FD-317 M1 | 2E-172 | 70.57 |
| LE_005487 | 59 | 870 | 3.88 | up | hypothetical protein GYMLUDRAFT_158832 | *G. luxurians* FD-317 M1 | 7E-43 | 77.08 |
| LE_007468 | 26 | 384 | 3.87 | up | glycoside hydrolase family 13 protein | *G. luxurians* FD-317 M1 | 0 | 87.40 |
| LE_010906 | 91 | 1,324 | 3.86 | up | hypothetical protein GYMLUDRAFT_36633 | *G. luxurians* FD-317 M1 | 0 | 73.77 |
| LE_009764 | 14 | 202 | 3.79 | up | hypothetical protein M378DRAFT_170652 | *A. muscaria* Koide BX008 | 4E-66 | 42.81 |
| LE_003162 | 104 | 1,428 | 3.77 | up | hypothetical protein GYMLUDRAFT_33117 | *G. luxurians* FD-317 M1 | 5E-75 | 75.60 |
| LE_007010 | 278 | 3,778 | 3.76 | up | Copper resistance protein CRF1 | *Termitomyces sp.* J132 | 3E-59 | 39.13 |
| LE_006815 | 22 | 291 | 3.74 | up | AMP binding protein | *C. cinerea* okayama7#130 | 0 | 53.52 |
| LE_007155 | 20 | 278 | 3.74 | up | hypothetical protein GYMLUDRAFT_600536 | *G. luxurians* FD-317 M1 | 6E-73 | 42.59 |
| LE_011154 | 11 | 152 | 3.72 | up | ribonuclease-domain-containing protein | *F. hepatica* ATCC 64428 | 4E-57 | 69.92 |
| LE_006221 | 13 | 172 | 3.71 | up | putative glycosidase C21B10.07 | *Termitomyces sp.* J132 | 9E-148 | 68.33 |
| LE_006281 | 8 | 102 | 3.70 | up | glycoside hydrolase family 30 protein | *G. luxurians* FD-317 M1 | 0 | 76.46 |
| LE_007737 | 8 | 108 | 3.69 | up | hypothetical protein GYMLUDRAFT_160389 | *G. luxurians* FD-317 M1 | 0 | 75.32 |
| LE_007094 | 16 | 216 | 3.69 | up | polyprotein | *P. chrysosporium* RP-78 | 1E-62 | 38.59 |
| LE_007475 | 17 | 224 | 3.68 | up | hypothetical protein GYMLUDRAFT_271892 | *G. luxurians* FD-317 M1 | 6E-44 | 76.64 |
| LE_006789 | 9 | 121 | 3.67 | up | hypothetical protein GYMLUDRAFT_198300 | *G. luxurians* FD-317 M1 | 3E-106 | 38.75 |
| LE_006207 | 26 | 334 | 3.65 | up |  |  |  |  |
| LE_001942 | 47 | 602 | 3.65 | up | hypothetical protein GYMLUDRAFT_42556 | *G. luxurians* FD-317 M1 | 2E-104 | 76.33 |
| LE_009426 | 42 | 531 | 3.64 | up | hypothetical protein GYMLUDRAFT_254265 | *G. luxurians* FD-317 M1 | 0 | 66.29 |
| LE_007512 | 48 | 599 | 3.63 | up | CIPA | *L. edodes* | 3E-88 | 87.62 |
| LE_006817 | 9 | 115 | 3.63 | up | hypothetical protein GYMLUDRAFT_44191 | *G. luxurians* FD-317 M1 | 0 | 81.89 |
| LE_010893 | 16 | 195 | 3.62 | up | hypothetical protein GYMLUDRAFT_34109 | *G. luxurians* FD-317 M1 | 0 | 82.59 |
| LE_011000 | 9 | 110 | 3.58 | up |  |  |  |  |
| LE_011388 | 37 | 442 | 3.55 | up | Fasciclin-domain-containing protein | *S. paradoxa* | 4E-37 | 38.14 |
| LE_006701 | 46 | 531 | 3.53 | up | hypothetical protein GYMLUDRAFT_239702 | *G. luxurians* FD-317 M1 | 3E-85 | 65.04 |
| LE_011386 | 140 | 1,614 | 3.52 | up |  |  |  |  |
| LE_009894 | 16 | 187 | 3.51 | up | hypothetical protein K443DRAFT_95892 | *L. amethystina* LaAM-08-1 | 1E-106 | 38.05 |
| LE_007204 | 35 | 400 | 3.50 | up | hypothetical protein GYMLUDRAFT_75426 | *G. luxurians* FD-317 M1 | 1E-133 | 79.75 |
| LE_006453 | 37 | 418 | 3.49 | up | predicted protein | *L. bicolor* S238N-H82 | 4E-38 | 30.39 |
| LE_010340 | 9 | 103 | 3.45 | up | lentiavidin1 | *L. edodes* | 6E-57 | 62.50 |
| LE_005270 | 12 | 132 | 3.44 | up | hypothetical protein GYMLUDRAFT_160655 | *G. luxurians* FD-317 M1 | 2E-64 | 63.10 |
| LE_007445 | 29 | 314 | 3.41 | up |  |  |  |  |
| LE_005290 | 12 | 129 | 3.40 | up | hypothetical protein GYMLUDRAFT_250607 | *G. luxurians* FD-317 M1 | 0 | 73.10 |
| LE_005719 | 39 | 417 | 3.40 | up | hypothetical protein GYMLUDRAFT_158610 | *G. luxurians* FD-317 M1 | 0 | 72.82 |
| LE_007483 | 14 | 152 | 3.39 | up | hypothetical protein GYMLUDRAFT_37111 | *G. luxurians* FD-317 M1 | 4E-45 | 59.06 |
| LE_006340 | 18 | 191 | 3.39 | up | cytochrome P450 family protein | *R. solani* 123E | 8E-94 | 36.62 |
| LE_003671 | 51 | 536 | 3.38 | up | Poly(A) RNA polymerase cid14 | *Termitomyces sp.* J132 | 1E-165 | 46.83 |
| LE_007449 | 12 | 131 | 3.37 | up | hypothetical protein STEHIDRAFT_125295 | *S. hirsutum* FP-91666 SS1 | 5E-35 | 32.67 |
| LE_003530 | 99 | 1,018 | 3.35 | up | hypothetical protein GYMLUDRAFT_43936 | *G. luxurians* FD-317 M1 | 0 | 82.21 |
| LE_006283 | 10 | 106 | 3.33 | up | cytochrome P450 monooxygenase 79 | *H. irregulare* TC 32-1 | 6E-88 | 39.71 |
| LE_006990 | 11 | 110 | 3.32 | up | hypothetical protein GYMLUDRAFT_64252 | *G. luxurians* FD-317 M1 | 0 | 55.06 |
| LE_002376 | 13 | 131 | 3.31 | up | hypothetical protein GYMLUDRAFT_74145 | *G. luxurians* FD-317 M1 | 1E-177 | 70.71 |
| LE_006692 | 28 | 277 | 3.31 | up | hypothetical protein GYMLUDRAFT_343839 | *G. luxurians* FD-317 M1 | 0 | 50.65 |
| LE_007348 | 76 | 756 | 3.31 | up | hypothetical protein GYMLUDRAFT_48009 | *G. luxurians* FD-317 M1 | 2E-41 | 46.28 |
| LE_005085 | 390 | 3,844 | 3.30 | up | hypothetical protein GYMLUDRAFT_66875 | *G. luxurians* FD-317 M1 | 2E-87 | 85.09 |
| LE_003585 | 21 | 214 | 3.29 | up | serine/threonine-protein kinase haspin | *Termitomyces sp.* J132 | 0 | 44.99 |
| LE_005740 | 43 | 414 | 3.27 | up | hypothetical protein GYMLUDRAFT_83637 | *G. luxurians* FD-317 M1 | 0 | 74.17 |
| LE_000988 | 64 | 609 | 3.25 | up | hypothetical protein GYMLUDRAFT_257086 | *G. luxurians* FD-317 M1 | 0 | 59.19 |
| LE_006648 | 88 | 843 | 3.25 | up | MFS general substrate transporter | *S. paradoxa* | 0 | 72.77 |
| LE_005068 | 14 | 135 | 3.24 | up | hypothetical protein GYMLUDRAFT_36632 | *G. luxurians* FD-317 M1 | 0 | 80.81 |
| LE_005258 | 12 | 119 | 3.22 | up | hypothetical protein GYMLUDRAFT_66815 | *G. luxurians* FD-317 M1 | 0 | 73.31 |
| LE_007452 | 141 | 1,319 | 3.22 | up | hypothetical protein GYMLUDRAFT_217920 | *G. luxurians* FD-317 M1 | 4E-122 | 69.90 |
| LE_008423 | 24 | 227 | 3.21 | up |  |  |  |  |
| LE_004412 | 13 | 125 | 3.20 | up |  |  |  |  |
| LE_009523 | 23 | 215 | 3.20 | up | hypothetical protein GYMLUDRAFT_230645 | *G. luxurians* FD-317 M1 | 3E-117 | 69.65 |
| LE_004391 | 16 | 147 | 3.20 | up | hypothetical protein GYMLUDRAFT_252319 | *G. luxurians* FD-317 M1 | 0 | 63.56 |
| LE_004756 | 50 | 460 | 3.18 | up | hypothetical protein GYMLUDRAFT_40360 | *G. luxurians* FD-317 M1 | 5E-93 | 54.13 |
| LE_007455 | 62 | 565 | 3.17 | up | hypothetical protein GYMLUDRAFT_32760 | *G. luxurians* FD-317 M1 | 1E-65 | 70.51 |
| LE_006729 | 21 | 194 | 3.17 | up | hypothetical protein GYMLUDRAFT_52914 | *G. luxurians* FD-317 M1 | 5E-52 | 35.20 |
| LE_004303 | 27 | 237 | 3.15 | up | transcription factor tau subunit *sfc6* | *Termitomyces sp.* J132 | 1E-172 | 55.01 |
| LE_007870 | 63 | 563 | 3.15 | up | hypothetical protein MPER_16276 | *M. perniciosa* FA553 | 3E-12 | 94.29 |
| LE_011214 | 18 | 165 | 3.15 | up | hypothetical protein GYMLUDRAFT_342872 | *G. luxurians* FD-317 M1 | 0 | 78.43 |
| LE_001678 | 75 | 659 | 3.13 | up | hypothetical protein GYMLUDRAFT_36186 | *G. luxurians* FD-317 M1 | 3E-53 | 43.43 |
| LE_010148 | 47 | 413 | 3.12 | up | Pkinase-domain-containing protein | *C. torrendii* FP15055 ss-10 | 1E-176 | 61.03 |
| LE_006974 | 21 | 181 | 3.12 | up |  |  |  |  |
| LE_002531 | 30 | 259 | 3.10 | up | hypothetical protein GYMLUDRAFT_34552 | *G. luxurians* FD-317 M1 | 0 | 75.75 |
| LE_007252 | 40 | 343 | 3.10 | up | hypothetical protein GYMLUDRAFT_145527 | *G. luxurians* FD-317 M1 | 2E-46 | 87.50 |
| LE_008237 | 55 | 468 | 3.08 | up | Pkinase-domain-containing protein | *C. torrendii* FP15055 ss-10 | 9E-124 | 61.67 |
| LE_003932 | 64 | 542 | 3.07 | up | alcohol oxidase | *S. hirsutum* FP-91666 SS1 | 1E-129 | 60.91 |
| LE_011094 | 42 | 355 | 3.07 | up | hypothetical protein GYMLUDRAFT_206211 | *G. luxurians* FD-317 M1 | 4E-21 | 78.33 |
| LE_010460 | 36 | 304 | 3.06 | up | hypothetical protein GYMLUDRAFT_35178 | *G. luxurians* FD-317 M1 | 5E-77 | 49.62 |
| LE_001114 | 153 | 1,262 | 3.04 | up | hypothetical protein GYMLUDRAFT_48351 | *G. luxurians* FD-317 M1 | 0 | 71.37 |
| LE_010779 | 23 | 188 | 3.02 | up | hypothetical protein GYMLUDRAFT_59780 | *G. luxurians* FD-317 M1 | 4E-29 | 54.79 |
| LE_007020 | 37 | 302 | 3.02 | up |  |  |  |  |
| LE_004229 | 28 | 228 | 3.02 | up | Zn finger family DNA binding protein | *L. bicolor* S238N-H82 | 6E-74 | 39.79 |
| LE_006067 | 358 | 2,889 | 3.01 | up | GMC oxidoreductase | *C. torrendii* FP15055 ss-10 | 0 | 87.48 |
| LE_006537 | 14 | 114 | 3.00 | up | DNA repair and recombination protein RAD26 | *Termitomyces sp.* J132 | 0 | 49.64 |
| LE_010347 | 14 | 117 | 2.99 | up | hypothetical protein GYMLUDRAFT_956060 | *G. luxurians* FD-317 M1 | 6E-122 | 46.86 |
| LE_006271 | 28 | 223 | 2.99 | up | endo-beta-1,3-glucanase | *Lentinula edodes* | 4E-165 | 99.63 |
| LE_005427 | 14 | 109 | 2.98 | up | Crm1-F1 | *C. cinerea* okayama7#130 | 0 | 62.61 |
| LE_006464 | 17 | 132 | 2.96 | up | dehydrogenase | *F. mediterranea* MF3/22 | 2E-70 | 40.06 |
| LE_010297 | 38 | 299 | 2.95 | up | P-loop containing nucleoside triphosphate hydrolase protein | *C. torrendii* FP15055 ss-10 | 4E-88 | 47.56 |
| LE_007443 | 1,010 | 7,749 | 2.94 | up | hypothetical protein GYMLUDRAFT_911550 | *G. luxurians* FD-317 M1 | 5E-47 | 62.26 |
| LE_003975 | 104 | 794 | 2.93 | up | MFS multidrug transporter | *S. hirsutum* FP-91666 SS1 | 0 | 67.91 |
| LE_006547 | 51 | 394 | 2.92 | up | hypothetical protein GYMLUDRAFT_215616 | *G. luxurians* FD-317 M1 | 2E-117 | 77.27 |
| LE_003957 | 116 | 877 | 2.91 | up | hypothetical protein GYMLUDRAFT_85311 | *G. luxurians* FD-317 M1 | 0E+00 | 84.87 |
| LE_011538 | 16 | 125 | 2.91 | up |  |  |  |  |
| LE_006397 | 19 | 148 | 2.90 | up | hypothetical protein GYMLUDRAFT_107944, partial | *G. luxurians* FD-317 M1 | 5E-28 | 75.34 |
| LE_004524 | 24 | 178 | 2.88 | up | P-loop containing nucleoside triphosphate hydrolase protein | *F. hepatica* ATCC 64428 | 0 | 60.75 |
| LE_003442 | 39 | 289 | 2.88 | up | hypothetical protein GYMLUDRAFT_37113 | *G. luxurians* FD-317 M1 | 5E-66 | 58.77 |
| LE_001051 | 352 | 2,560 | 2.86 | up | hypothetical protein GYMLUDRAFT_58517 | *G. luxurians* FD-317 M1 | 0 | 54.95 |
| LE_010281 | 18 | 134 | 2.85 | up | P-loop containing nucleoside triphosphate hydrolase protein | *F. hepatica* ATCC 64428 | 0 | 61.16 |
| LE_000930 | 276 | 1,972 | 2.83 | up | Ankyrin repeat domain-containing protein 50 | *Termitomyces sp.* J132 | 2E-148 | 39.21 |
| LE_002014 | 42 | 305 | 2.83 | up | glycoside hydrolase family 71 protein | *G. luxurians* FD-317 M1 | 0 | 70.11 |
| LE_006004 | 74 | 530 | 2.83 | up | hypothetical protein GYMLUDRAFT_33762 | *G. luxurians* FD-317 M1 | 0 | 64.33 |
| LE_004060 | 217 | 1,545 | 2.83 | up | glycoside hydrolase family 79 protein | *G. luxurians* FD-317 M1 | 0 | 81.18 |
| LE_006975 | 33 | 235 | 2.81 | up | hypothetical protein GYMLUDRAFT_36896 | *G. luxurians* FD-317 M1 | 2E-68 | 50.00 |
| LE_006588 | 213 | 1,495 | 2.81 | up | hypothetical protein K443DRAFT_672586 | *L. amethystina* LaAM-08-1 | 3E-98 | 68.42 |
| LE_008606 | 73 | 505 | 2.79 | up | hypothetical protein GYMLUDRAFT_734761 | *G. luxurians* FD-317 M1 | 9E-53 | 40.28 |
| LE_003047 | 964 | 6,606 | 2.78 | up | putative high affinity glucose transporter | *C. torrendii* FP15055 ss-10 | 0 | 78.17 |
| LE_000653 | 177 | 1,209 | 2.77 | up | ornithine-oxo-acid aminotransferase | *L. bicolor* S238N-H82 | 0 | 83.61 |
| LE_007303 | 35 | 238 | 2.77 | up | glycoside hydrolase family 18 protein | *G. luxurians* FD-317 M1 | 4E-133 | 60.97 |
| LE_002940 | 569 | 3,817 | 2.75 | up | general substrate transporter | *F. hepatica* ATCC 64428 | 0 | 71.75 |
| LE_011141 | 23 | 152 | 2.73 | up | white collar photoreceptors-like protein | *L. edodes* | 0 | 94.17 |
| LE_002019 | 46 | 306 | 2.72 | up | Alcohol dehydrogenase 3 | *Termitomyces sp.* J132 | 3E-118 | 53.74 |
| LE_003809 | 30 | 200 | 2.71 | up | inorganic phosphate transporter | *H. irregulare* TC 32-1 | 0 | 76.37 |
| LE_007487 | 20 | 135 | 2.71 | up | hypothetical protein CERSUDRAFT_145806 | *G. subvermispora* B | 3E-62 | 40.94 |
| LE_010877 | 41 | 267 | 2.70 | up | hypothetical protein GYMLUDRAFT_241246 | *G. luxurians* FD-317 M1 | 0 | 60.99 |
| LE_005408 | 31 | 202 | 2.69 | up | carnitine acetyl transferase | *C. cinerea* okayama7#130 | 0 | 75.35 |
| LE_001162 | 43 | 276 | 2.69 | up | polysaccharide lyase family 14 protein | *P. croceum* F 1598 | 2E-97 | 63.87 |
| LE_011237 | 22 | 142 | 2.67 | up | hypothetical protein GYMLUDRAFT_34761 | *G. luxurians* FD-317 M1 | 9E-136 | 84.17 |
| LE_010800 | 16 | 104 | 2.66 | up | hypothetical protein GYMLUDRAFT_888548 | *G. luxurians* FD-317 M1 | 0 | 59.66 |
| LE_010774 | 33 | 207 | 2.65 | up | hypothetical protein BN946_scf184298.g2 | *T. cinnabarina* | 3E-45 | 36.89 |
| LE_004273 | 18 | 115 | 2.64 | up | hypothetical protein GYMLUDRAFT_45835 | *G. luxurians* FD-317 M1 | 4E-137 | 60.54 |
| LE_010547 | 18 | 113 | 2.63 | up | hypothetical protein HYPSUDRAFT_90981 | *H. sublateritium* FD-334 SS-4 | 7E-34 | 57.66 |
| LE_010028 | 17 | 106 | 2.58 | up | hypothetical protein GYMLUDRAFT_181158 | *G. luxurians* FD-317 M1 | 6E-37 | 87.67 |
| LE_006626 | 103 | 617 | 2.58 | up | hypothetical protein GYMLUDRAFT_82836 | *G. luxurians* FD-317 M1 | 1E-114 | 51.59 |
| LE_009007 | 30 | 180 | 2.56 | up | RNA-directed DNA polymerase from mobile element jockey, partial | *Termitomyces sp.* J132 | 9E-175 | 46.86 |
| LE_007574 | 22 | 130 | 2.54 | up | RNA-binding domain-containing protein | *S. hirsutum* FP-91666 SS1 | 1E-38 | 74.71 |
| LE_004885 | 35 | 205 | 2.54 | up | hypothetical protein PILCRDRAFT_828042 | *P. croceum* F 1598 | 2E-88 | 37.97 |
| LE_005488 | 120 | 697 | 2.54 | up | TonB box-containing protein | *L. bicolor* S238N-H82 | 9E-61 | 55.1 |
| LE_006852 | 21 | 127 | 2.54 | up | Origin recognition complex subunit 2 | *Termitomyces sp.* J132 | 1E-143 | 54.63 |
| LE_010775 | 40 | 234 | 2.53 | up | hypothetical protein GYMLUDRAFT_49165 | *G. luxurians* FD-317 M1 | 0 | 70.02 |
| LE_007880 | 23 | 134 | 2.52 | up | IQ calmodulin-binding domain protein, putative | *R. solani* AG-3 Rhs1AP | 3E-110 | 50.00 |
| LE_010248 | 28 | 163 | 2.52 | up | hypothetical protein GYMLUDRAFT_153104 | *G. luxurians* FD-317 M1 | 0 | 63.96 |
| LE_010317 | 27 | 155 | 2.51 | up | hypothetical protein GYMLUDRAFT_50564 | *G. luxurians* FD-317 M1 | 0 | 82.14 |
| LE_009437 | 38 | 221 | 2.51 | up | hypothetical protein GYMLUDRAFT_247141 | *G. luxurians* FD-317 M1 | 0 | 58.57 |
| LE_006773 | 19 | 110 | 2.51 | up | putative mfs transporter protein | *N. parvum* UCRNP2 | 0 | 72.15 |
| LE_007593 | 20 | 115 | 2.49 | up | hypothetical protein GYMLUDRAFT_148385 | *G. luxurians* FD-317 M1 | 1E-143 | 68.09 |
| LE_006209 | 34 | 191 | 2.49 | up | hypothetical protein GYMLUDRAFT_62515 | *G. luxurians* FD-317 M1 | 0 | 63.06 |
| LE_000937 | 296 | 1,644 | 2.47 | up | putative fungal specific transcription factor | *L. edodes* | 0 | 99.27 |
| LE_001690 | 33 | 183 | 2.46 | up | hypothetical protein GYMLUDRAFT_68403 | *G. luxurians* FD-317 M1 | 0 | 66.89 |
| LE_001562 | 206 | 1,131 | 2.46 | up | putative ATP-dependent RNA helicase prh1 | *Termitomyces sp.* J132 | 0 | 67.17 |
| LE_006092 | 38 | 207 | 2.46 | up | glycoside hydrolase family 18 protein | *G. luxurians* FD-317 M1 | 0 | 73.27 |
| LE_006419 | 602 | 3,274 | 2.44 | up | hypothetical protein GYMLUDRAFT_249051 | *G. luxurians* FD-317 M1 | 0 | 78.18 |
| LE_009856 | 20 | 112 | 2.44 | up |  |  |  |  |
| LE_010427 | 20 | 109 | 2.43 | up | hypothetical protein GYMLUDRAFT_278627 | *G. luxurians* FD-317 M1 | 8E-17 | 46.07 |
| LE_009936 | 49 | 267 | 2.43 | up | hypothetical protein GYMLUDRAFT_62302 | *G. luxurians* FD-317 M1 | 5E-48 | 45.30 |
| LE_004557 | 98 | 529 | 2.43 | up | hypothetical protein GYMLUDRAFT_67778 | *G. luxurians* FD-317 M1 | 5E-141 | 41.37 |
| LE_005309 | 38 | 205 | 2.43 | up | palmitoyl-protein thioesterase | *C. cinerea* okayama7#130 | 2E-144 | 67.52 |
| LE_009324 | 25 | 134 | 2.41 | up |  |  |  |  |
| LE_005400 | 3,161 | 16,775 | 2.41 | up | pyruvate decarboxylase THI3 | *G. trabeum* ATCC 11539 | 0 | 69.41 |
| LE_000121 | 148 | 781 | 2.40 | up | lysyl-tRNA synthetase | *T. rubrum* CBS 118892 | 2E-169 | 43.59 |
| LE_005440 | 179 | 941 | 2.40 | up | hypothetical protein GYMLUDRAFT_51003 | *G. luxurians* FD-317 M1 | 1E-88 | 58.79 |
| LE_003733 | 163 | 851 | 2.38 | up | hypothetical protein GYMLUDRAFT_170208 | *G. luxurians* FD-317 M1 | 0 | 84.53 |
| LE_003603 | 170 | 882 | 2.37 | up | hypothetical protein GYMLUDRAFT_156931 | *G. luxurians* FD-317 M1 | 0 | 80.26 |
| LE_006256 | 24 | 124 | 2.37 | up | hypothetical protein GYMLUDRAFT_177527 | *G. luxurians* FD-317 M1 | 5E-62 | 53.88 |
| LE_003969 | 383 | 1,968 | 2.36 | up | Indoleamine 23-dioxygenase | *M. phaseolina* MS6 | 0 | 68.94 |
| LE_004478 | 26 | 135 | 2.35 | up | hypothetical protein GYMLUDRAFT_207526 | *G. luxurians* FD-317 M1 | 0 | 67.39 |
| LE_006233 | 124 | 634 | 2.35 | up | hypothetical protein GYMLUDRAFT_76191 | *G. luxurians* FD-317 M1 | 0 | 77.60 |
| LE_005582 | 32 | 164 | 2.34 | up | hypothetical protein GYMLUDRAFT_47284 | *G. luxurians* FD-317 M1 | 4E-149 | 46.30 |
| LE_006608 | 32 | 165 | 2.34 | up | hypothetical protein GYMLUDRAFT_255303 | *G. luxurians* FD-317 M1 | 0 | 59.42 |
| LE_005129 | 24 | 123 | 2.33 | up |  |  |  |  |
| LE_005272 | 108 | 543 | 2.33 | up | hypothetical protein GYMLUDRAFT_576703 | *G. luxurians* FD-317 M1 | 0 | 77.51 |
| LE_003979 | 164 | 814 | 2.31 | up | hypothetical protein GYMLUDRAFT_82433 | *G. luxurians* FD-317 M1 | 0 | 63.62 |
| LE_007930 | 22 | 109 | 2.31 | up | hypothetical protein GYMLUDRAFT_259993 | *G. luxurians* FD-317 M1 | 4E-42 | 43.39 |
| LE_010878 | 36 | 181 | 2.31 | up | hypothetical protein GYMLUDRAFT_188580, partial | *G. luxurians* FD-317 M1 | 3E-169 | 71.55 |
| LE_009910 | 120 | 588 | 2.29 | up | hypothetical protein GYMLUDRAFT_163684 | *G. luxurians* FD-317 M1 | 0 | 79.21 |
| LE_010277 | 84 | 408 | 2.28 | up | tridomain enzyme adenylation-thiolation-dehydrogenase | *H. irregulare* TC 32-1 | 1E-175 | 35.04 |
| LE_002605 | 615 | 2,963 | 2.27 | up | hypothetical protein GALMADRAFT_138398 | *G. marginata* CBS 339.88 | 0 | 93.91 |
| LE_006699 | 265 | 1,252 | 2.24 | up | hypothetical protein GYMLUDRAFT_247260 | *G. luxurians* FD-317 M1 | 0E+00 | 83.66 |
| LE_004279 | 21 | 100 | 2.23 | up | SET domain-containing protein | *D. squalens* LYAD-421 SS1 | 9E-117 | 48.24 |
| LE_006523 | 57 | 270 | 2.23 | up | hypothetical protein GYMLUDRAFT_662385 | *G. luxurians* FD-317 M1 | 2E-171 | 58.29 |
| LE_007720 | 48 | 229 | 2.23 | up | hypothetical protein GYMLUDRAFT_263155 | *G. luxurians* FD-317 M1 | 4E-15 | 70.00 |
| LE_008597 | 27 | 127 | 2.22 | up | hypothetical protein GYMLUDRAFT_44908 | *G. luxurians* FD-317 M1 | 4E-72 | 59.14 |
| LE_006788 | 30 | 137 | 2.19 | up | MFS polyamine transporter | *L. bicolor* S238N-H82 | 0 | 63.34 |
| LE_001210 | 145 | 663 | 2.19 | up | hypothetical protein GYMLUDRAFT_237695 | *G. luxurians* FD-317 M1 | 5E-91 | 52.04 |
| LE_006689 | 24 | 110 | 2.19 | up |  |  |  |  |
| LE_010778 | 39 | 181 | 2.18 | up | hypothetical protein GYMLUDRAFT_261825 | *G. luxurians* FD-317 M1 | 0 | 70.91 |
| LE_005791 | 37 | 167 | 2.18 | up | hypothetical protein GYMLUDRAFT_60283 | *G. luxurians* FD-317 M1 | 4E-125 | 48.09 |
| LE_005143 | 43 | 195 | 2.18 | up |  |  |  |  |
| LE_006190 | 202 | 915 | 2.18 | up | hypothetical protein GYMLUDRAFT_38132 | *G. luxurians* FD-317 M1 | 0E+00 | 63.21 |
| LE_000498 | 54 | 243 | 2.18 | up | hypothetical protein GYMLUDRAFT_74874 | *G. luxurians* FD-317 M1 | 3E-132 | 72.82 |
| LE_006313 | 168 | 755 | 2.17 | up | aldehyde dehydrogenase 6 | *T. vaccinum* | 0 | 77.04 |
| LE_007565 | 48 | 218 | 2.16 | up | S-adenosyl-L-methionine-dependent methyltransferase | *C. torrendii* FP15055 ss-10 | 0 | 70.63 |
| LE_005918 | 47 | 211 | 2.15 | up |  |  |  |  |
| LE_004362 | 54 | 240 | 2.14 | up | hypothetical protein GYMLUDRAFT_60133 | *G. luxurians* FD-317 M1 | 1E-48 | 41.88 |
| LE_003370 | 97 | 428 | 2.14 | up | glycoside hydrolase family 79 protein | *H. sublateritium* FD-334 SS-4 | 0 | 54.89 |
| LE_009388 | 40 | 176 | 2.13 | up | hypothetical protein GYMLUDRAFT_244250 | *G. luxurians* FD-317 M1 | 2E-18 | 34.41 |
| LE_002628 | 64 | 279 | 2.13 | up | glycoside hydrolase family 2 protein | *G. luxurians* FD-317 M1 | 0 | 75.57 |
| LE_001839 | 37 | 163 | 2.12 | up | hypothetical protein GYMLUDRAFT_767922 | *G. luxurians* FD-317 M1 | 4E-149 | 53.82 |
| LE_008875 | 301 | 1,306 | 2.11 | up | copia-type pol polyprotein | *Z. mays* | 2E-101 | 30.21 |
| LE_009587 | 64 | 278 | 2.11 | up |  |  |  |  |
| LE_004625 | 850 | 3,663 | 2.11 | up | L-iditol 2-dehydrogenase | *P. involutus* ATCC 200175 | 0 | 90.14 |
| LE_004626 | 237 | 1,021 | 2.11 | up | L-iditol 2-dehydrogenase | *P. involutus* ATCC 200175 | 3E-48 | 86.17 |
| LE_010866 | 23 | 100 | 2.10 | up | laccase | *L. edodes* | 0 | 100.00 |
| LE_006118 | 46 | 198 | 2.10 | up | hypothetical protein GYMLUDRAFT_38475 | *G. luxurians* FD-317 M1 | 4E-101 | 56.74 |
| LE_002908 | 1,629 | 6,964 | 2.10 | up | hypothetical protein GYMLUDRAFT_41898 | *G. luxurians* FD-317 M1 | 0 | 77.66 |
| LE_003874 | 101 | 424 | 2.07 | up | hypothetical protein GYMLUDRAFT_149990 | *G. luxurians* FD-317 M1 | 3E-48 | 75.41 |
| LE_006125 | 31 | 129 | 2.06 | up |  |  |  |  |
| LE_006968 | 30 | 126 | 2.06 | up | hypothetical protein GYMLUDRAFT_39456 | *G. luxurians* FD-317 M1 | 7E-21 | 61.42 |
| LE_000916 | 2,028 | 8,433 | 2.06 | up | phosphatidylserine decarboxylase 1 | *G. frondosa* | 5E-157 | 58.26 |
| LE_003448 | 68 | 285 | 2.05 | up | hypothetical protein GYMLUDRAFT_36115 | *G. luxurians* FD-317 M1 | 7E-95 | 52.23 |
| LE_003821 | 41 | 171 | 2.05 | up | hypothetical protein GYMLUDRAFT_46454 | *G. luxurians* FD-317 M1 | 9E-111 | 82.05 |
| LE_003268 | 32 | 134 | 2.05 | up | hypothetical protein GYMLUDRAFT_154015 | *G. luxurians* FD-317 M1 | 3E-156 | 68.77 |
| LE_006217 | 33 | 139 | 2.05 | up | cytochrome P450 | *P. strigosozonata* HHB-11173 SS5 | 0 | 59.42 |
| LE_006012 | 30 | 124 | 2.03 | up | DNA ligase/mRNA capping enzyme, partial | *P. strigosozonata* HHB-11173 SS5 | 4E-134 | 42.20 |
| LE_001026 | 514 | 2,102 | 2.03 | up | hypothetical protein GYMLUDRAFT_47635 | *G. luxurians* FD-317 M1 | 3E-163 | 70.74 |
| LE_000698 | 1,115 | 4,562 | 2.03 | up | hypothetical protein GYMLUDRAFT_76488 | *G. luxurians* FD-317 M1 | 2E-140 | 74.19 |
| LE_006391 | 36 | 149 | 2.02 | up | glycosyltransferase family 90 protein | *G. luxurians* FD-317 M1 | 0 | 61.44 |
| LE_000219 | 2,478 | 10,019 | 2.02 | up | Quinate permease | *Termitomyces sp.* J132 | 0 | 72.50 |
| LE_006189 | 178 | 716 | 2.01 | up | hypothetical protein GYMLUDRAFT_450222 | *G. luxurians* FD-317 M1 | 2E-113 | 49.32 |
| LE_005507 | 59 | 236 | 2.00 | up | JmjC domain-containing protein E | *Termitomyces sp.* J132 | 1E-96 | 56.64 |
| LE_003489 | 169 | 676 | 2.00 | up |  |  |  |  |
| LE_003275 | 16,165 | 4,052 | -2.00 | down | hypothetical protein GYMLUDRAFT_33084 | *G. luxurians* FD-317 M1 | 4E-78 | 71.86 |
| LE_010272 | 1,352 | 339 | -2.00 | down | DUF221-domain-containing protein | *F. hepatica* ATCC 64428 | 0 | 65.96 |
| LE_002959 | 844 | 210 | -2.00 | down | hypothetical protein GYMLUDRAFT_198083 | *G. luxurians* FD-317 M1 | 4E-177 | 70.71 |
| LE_010078 | 286 | 71 | -2.01 | down | hypothetical protein GYMLUDRAFT_56781 | *G. luxurians* FD-317 M1 | 0 | 71.68 |
| LE_001462 | 429 | 106 | -2.01 | down | Pentulose kinase | *C. torrendii* FP15055 ss-10 | 0 | 74.04 |
| LE_009117 | 2,309 | 572 | -2.01 | down | hypothetical protein GYMLUDRAFT_49171 | *G. luxurians* FD-317 M1 | 3E-55 | 50.68 |
| LE_008197 | 800 | 198 | -2.01 | down | copper-amine-oxidase superfamily | *Pleurotus ostreatus* PC15 | 0 | 68.62 |
| LE_004073 | 1,538 | 380 | -2.01 | down | hypothetical protein GYMLUDRAFT_33607 | *G. luxurians* FD-317 M1 | 4E-169 | 53.77 |
| LE_002490 | 192 | 47 | -2.02 | down | major facilitator superfamily | *H. irregulare* TC 32-1 | 0 | 77.10 |
| LE_001418 | 1,247 | 307 | -2.02 | down | PAP2-domain-containing protein | *D. squalens* LYAD-421 SS1 | 8E-177 | 56.34 |
| LE_001997 | 367 | 90 | -2.02 | down | Deoxyhypusine hydroxylase |  | 0 | 96.10 |
| LE_001807 | 496 | 122 | -2.02 | down | sugar transporter | *S. hirsutum* FP-91666 SS1 | 0 | 68.33 |
| LE_003913 | 360 | 88 | -2.02 | down | hypothetical protein GYMLUDRAFT_77217 | *G. luxurians* FD-317 M1 | 2E-165 | 61.44 |
| LE_001279 | 881 | 216 | -2.02 | down | ornithine decarboxylase | *C. cinerea* okayama7#130 | 0 | 68.51 |
| LE_002950 | 7,658 | 1,879 | -2.03 | down | hypothetical protein PLEOSDRAFT_1088686 | *P. ostreatus* PC15 | 0 | 93.35 |
| LE_000148 | 17,441 | 4,272 | -2.03 | down | hypothetical protein GYMLUDRAFT_264499 | *G. luxurians* FD-317 M1 | 3E-39 | 60.29 |
| LE_000925 | 365 | 89 | -2.03 | down | hypothetical protein GYMLUDRAFT_49647 | *G. luxurians* FD-317 M1 | 5E-174 | 64.34 |
| LE_002691 | 442 | 108 | -2.04 | down |  |  |  |  |
| LE_000894 | 512 | 125 | -2.04 | down |  |  |  |  |
| LE_001262 | 411 | 100 | -2.04 | down | hypothetical protein GYMLUDRAFT_73036 | *G. luxurians* FD-317 M1 | 0 | 75.75 |
| LE_001275 | 272 | 66 | -2.04 | down | hypothetical protein GYMLUDRAFT_37584 | *G. luxurians* FD-317 M1 | 9E-96 | 45.96 |
| LE_002933 | 912 | 221 | -2.04 | down | DNase I-like protein | *C. puteana* RWD-64-598 SS2 | 5E-142 | 41.14 |
| LE_002924 | 2,916 | 706 | -2.04 | down | hypothetical protein GYMLUDRAFT_44109 | *G. luxurians* FD-317 M1 | 0 | 88.47 |
| LE_008168 | 1,347 | 326 | -2.05 | down | C-1-tetrahydrofolate synthase, cytoplasmic | *Termitomyces sp.* J132 | 0 | 84.77 |
| LE_002947 | 542 | 130 | -2.05 | down | hypothetical protein GYMLUDRAFT_101115 | *G. luxurians* FD-317 M1 | 1E-73 | 38.17 |
| LE_001931 | 285 | 68 | -2.05 | down | hypothetical protein CONPUDRAFT_168850 | *C. puteana* RWD-64-598 SS2 | 2E-21 | 29.19 |
| LE_010064 | 109 | 26 | -2.06 | down | hypothetical protein GYMLUDRAFT_949192 | *G. luxurians* FD-317 M1 | 0 | 65.64 |
| LE_003315 | 420 | 100 | -2.06 | down | carbohydrate-binding module family 35 protein | *G. luxurians* FD-317 M1 | 0 | 79.68 |
| LE_001826 | 109 | 26 | -2.06 | down | hypothetical protein GYMLUDRAFT_264802 | *G. luxurians* FD-317 M1 | 5E-155 | 74.75 |
| LE_001655 | 320 | 76 | -2.06 | down | hypothetical protein GYMLUDRAFT_34639 | *G. luxurians* FD-317 M1 | 0 | 74.15 |
| LE_008640 | 362 | 86 | -2.06 | down | Formate/glycerate dehydrogenase catalytic domain-like protein | *G. trabeum* ATCC 11539 | 0 | 80.56 |
| LE_008135 | 2,893 | 692 | -2.06 | down | NAD-malate dehydrogenase | *G. trabeum* ATCC 11539 | 6E-167 | 82.01 |
| LE_002079 | 992 | 237 | -2.06 | down |  |  |  |  |
| LE_000267 | 1,601 | 382 | -2.07 | down | glycoside hydrolase family 27 protein | *G. luxurians* FD-317 M1 | 0 | 81.10 |
| LE_002396 | 132 | 31 | -2.07 | down | hypothetical protein GYMLUDRAFT_157146 | *G. luxurians* FD-317 M1 | 3E-151 | 86.31 |
| LE_009397 | 507 | 120 | -2.07 | down | hypothetical protein GYMLUDRAFT_41375 | *G. luxurians* FD-317 M1 | 6E-133 | 49.59 |
| LE_004502 | 805 | 190 | -2.08 | down |  |  |  |  |
| LE_009525 | 136 | 32 | -2.08 | down | hypothetical protein GYMLUDRAFT_47377 | *G. luxurians* FD-317 M1 | 0 | 71.76 |
| LE_003866 | 116 | 27 | -2.08 | down | glycosyltransferase family 1 protein | *G. luxurians* FD-317 M1 | 0 | 68.09 |
| LE_002925 | 1,567 | 369 | -2.08 | down | hypothetical protein GYMLUDRAFT_41927 | *G. luxurians* FD-317 M1 | 1E-144 | 87.17 |
| LE_003254 | 176 | 41 | -2.09 | down | Metallo-dependent phosphatase | *G. trabeum* ATCC 11539 | 1E-99 | 56.27 |
| LE_003580 | 351 | 82 | -2.09 | down | UDP-N-acetylglucosamine transporter YEA4 | *Termitomyces sp.* J132 | 1E-131 | 56.79 |
| LE_000378 | 7,523 | 1,754 | -2.10 | down | tetraspanin | *C. torrendii* FP15055 ss-10 | 2E-89 | 62.17 |
| LE_001075 | 345 | 80 | -2.10 | down | hypothetical protein GYMLUDRAFT_49829 | *G. luxurians* FD-317 M1 | 1E-72 | 84.71 |
| LE_004211 | 109 | 25 | -2.11 | down | hypothetical protein GYMLUDRAFT_147994 | *G. luxurians* FD-317 M1 | 0 | 79.10 |
| LE_000343 | 1,622 | 376 | -2.11 | down | glycoside hydrolase family 31 protein | *G. luxurians* FD-317 M1 | 0 | 79.57 |
| LE_002861 | 457 | 106 | -2.11 | down | hypothetical protein GYMLUDRAFT_917793 | *G. luxurians* FD-317 M1 | 0 | 82.51 |
| LE_000282 | 5,430 | 1,255 | -2.11 | down | hypothetical protein GYMLUDRAFT_38686 | *G. luxurians* FD-317 M1 | 4E-152 | 77.69 |
| LE_001835 | 167 | 38 | -2.11 | down |  |  |  |  |
| LE_009099 | 1,421 | 328 | -2.12 | down | hypothetical protein GYMLUDRAFT_66930 | *G. luxurians* FD-317 M1 | 0 | 69.90 |
| LE_008515 | 431 | 99 | -2.12 | down | hypothetical protein GYMLUDRAFT_40242 | *G. luxurians* FD-317 M1 | 0 | 61.74 |
| LE_003209 | 447 | 103 | -2.12 | down | hypothetical protein GYMLUDRAFT_251471 | *G. luxurians* FD-317 M1 | 3E-32 | 48.40 |
| LE_010268 | 3,162 | 729 | -2.12 | down |  |  |  |  |
| LE_000141 | 780 | 179 | -2.12 | down | Diphosphomevalonate decarboxylase | *C.* *torrendii* FP15055 ss-10 | 0 | 70.17 |
| LE_001248 | 491 | 113 | -2.12 | down | hypothetical protein CONPUDRAFT_149487 | *C.* *puteana* RWD-64-598 SS2 | 3E-66 | 48.72 |
| LE_004945 | 197 | 45 | -2.12 | down | enolase C-terminal domain-like protein | *P. strigosozonata* HHB-11173 SS5 | 0 | 75.82 |
| LE_008431 | 419 | 96 | -2.12 | down | hypothetical protein GYMLUDRAFT_46398 | *G. luxurians* FD-317 M1 | 2E-66 | 44.24 |
| LE_006081 | 103 | 23 | -2.12 | down | hypothetical protein GYMLUDRAFT_245213 | *G. luxurians* FD-317 M1 | 0 | 54.00 |
| LE_009123 | 1,176 | 270 | -2.12 | down | hypothetical protein GYMLUDRAFT_169798 | *G. luxurians* FD-317 M1 | 0 | 79.20 |
| LE_005244 | 102 | 23 | -2.13 | down | OPT superfamily | *P. ostreatus* PC15 | 0 | 72.61 |
| LE_003037 | 1,532 | 350 | -2.13 | down | hypothetical protein GYMLUDRAFT_40354 | *G. luxurians* FD-317 M1 | 0 | 87.52 |
| LE_008686 | 123 | 28 | -2.13 | down | hypothetical protein GYMLUDRAFT_238000 | *G. luxurians* FD-317 M1 | 3E-37 | 27.17 |
| LE_001792 | 303 | 69 | -2.14 | down | Aldo/keto reductase | *C. torrendii* FP15055 ss-10 | 1E-138 | 61.61 |
| LE_004170 | 138 | 31 | -2.14 | down | kinase-like protein | *G. trabeum* ATCC 11539 | 2E-65 | 37.29 |
| LE_001857 | 281 | 63 | -2.15 | down | hypothetical protein GYMLUDRAFT_34599 | *G. luxurians* FD-317 M1 | 0 | 69.77 |
| LE_000608 | 1,277 | 288 | -2.15 | down | hypothetical protein GYMLUDRAFT_63085 | *G. luxurians* FD-317 M1 | 6E-46 | 72.38 |
| LE_002081 | 395 | 89 | -2.15 | down | hypothetical protein GYMLUDRAFT_34387 | *G. luxurians* FD-317 M1 | 0 | 73.31 |
| LE_000864 | 4,326 | 967 | -2.16 | down | hypothetical protein GYMLUDRAFT_251345 | *G. luxurians* FD-317 M1 | 0 | 67.97 |
| LE_004046 | 1,055 | 236 | -2.16 | down | hypothetical protein GYMLUDRAFT_45275 | *G. luxurians* FD-317 M1 | 0 | 72.88 |
| LE_000720 | 493 | 110 | -2.16 | down | cyclopropane-fatty-acyl-phospholipid synthase | *A. bisporus var. bisporus* H97 | 0 | 77.44 |
| LE_003981 | 819 | 183 | -2.16 | down | CMGC/MAPK/ERK protein kinase | *C. torrendii* FP15055 ss-10 | 0 | 90.40 |
| LE_001554 | 179 | 40 | -2.16 | down | glycoside hydrolase family 53 protein | *G. luxurians* FD-317 M1 | 0 | 76.32 |
| LE_003059 | 141 | 31 | -2.17 | down | hypothetical protein GYMLUDRAFT_164440 | *G. luxurians* FD-317 M1 | 1E-150 | 74.92 |
| LE_000794 | 480 | 106 | -2.17 | down | hypothetical protein GYMLUDRAFT_39720 | *G. luxurians* FD-317 M1 | 1E-171 | 82.94 |
| LE_002265 | 135 | 30 | -2.17 | down | hypothetical protein GYMLUDRAFT_32709 | *G. luxurians* FD-317 M1 | 5E-35 | 39.19 |
| LE_002921 | 1,582 | 351 | -2.17 | down | hypothetical protein GYMLUDRAFT_981785 | *G. luxurians* FD-317 M1 | 0 | 86.77 |
| LE_000834 | 31,922 | 7,083 | -2.17 | down | Polyubiquitin 10 OS=Arabidopsis thaliana GN=UBQ10 PE=1 SV=2 | *R. solani* AG-1 IB | 0 | 88.08 |
| LE_008964 | 1,115 | 247 | -2.17 | down | hypothetical protein GYMLUDRAFT_892997 | *G. luxurians* FD-317 M1 | 0 | 63.92 |
| LE_004184 | 616 | 136 | -2.17 | down | hypothetical protein GYMLUDRAFT_263761 | *G. luxurians* FD-317 M1 | 6E-14 | 56.25 |
| LE_008228 | 2,922 | 646 | -2.18 | down | hypothetical protein GYMLUDRAFT_83464 | *G. luxurians* FD-317 M1 | 4E-96 | 72.95 |
| LE_000381 | 406 | 89 | -2.18 | down | MFS general substrate transporter | *C. torrendii* FP15055 ss-10 | 0E+00 | 54.28 |
| LE_004054 | 235 | 51 | -2.18 | down | biotin synthase, BioB | *L. bicolor* S238N-H82 | 0 | 81.92 |
| LE_003091 | 114 | 25 | -2.19 | down | hypothetical protein GYMLUDRAFT_44468 | *G. luxurians* FD-317 M1 | 1E-52 | 32.11 |
| LE_009586 | 1,615 | 354 | -2.19 | down | hypothetical protein GYMLUDRAFT_72036 | *G. luxurians* FD-317 M1 | 0 | 72.64 |
| LE_000248 | 2,179 | 476 | -2.19 | down | glycosyltransferase family 2 protein | *G. luxurians* FD-317 M1 | 0 | 87.89 |
| LE_008091 | 5,810 | 1,269 | -2.19 | down | UPF0187-domain-containing protein | *T. versicolor* FP-101664 SS1 | 2E-167 | 54.19 |
| LE_000188 | 5,313 | 1,154 | -2.20 | down | expressed protein | *S. commune* H4-8 | 6E-50 | 58.86 |
| LE_001181 | 3,834 | 831 | -2.21 | down | hypothetical protein GYMLUDRAFT_605748 | *G. luxurians* FD-317 M1 | 0 | 79.65 |
| LE_000245 | 1,099 | 238 | -2.21 | down | zf-U1-domain-containing protein | *P. strigosozonata* HHB-11173 SS5 | 2E-32 | 96.00 |
| LE_000385 | 11,302 | 2,446 | -2.21 | down | hypothetical protein GYMLUDRAFT_79342 | *G. luxurians* FD-317 M1 | 0 | 68.57 |
| LE_008403 | 1,939 | 418 | -2.21 | down | hypothetical protein GYMLUDRAFT_157963 | *G. luxurians* FD-317 M1 | 5E-59 | 68.79 |
| LE_002979 | 738 | 159 | -2.22 | down | hypothetical protein GYMLUDRAFT_154798 | *G. luxurians* FD-317 M1 | 0 | 71.58 |
| LE_003206 | 1,329 | 285 | -2.22 | down | hypothetical protein GYMLUDRAFT_42191 | *G. luxurians* FD-317 M1 | 0 | 84.81 |
| LE_009080 | 273 | 58 | -2.22 | down | short chain oxidoreductase | *S. hirsutum* FP-91666 SS1 | 2E-80 | 55.73 |
| LE_008786 | 270 | 58 | -2.22 | down | hypothetical protein GYMLUDRAFT_48202 | *G. luxurians* FD-317 M1 | 0 | 83.84 |
| LE_008904 | 503 | 107 | -2.22 | down | oxidase, peroxisomal | *L. bicolor* S238N-H82 | 0E+00 | 61.11 |
| LE_008161 | 1,201 | 256 | -2.22 | down |  |  |  |  |
| LE_000541 | 1,316 | 281 | -2.23 | down | ribonuclease T2 | *L. edodes* | 0 | 99.63 |
| LE_001585 | 143 | 30 | -2.23 | down |  |  |  |  |
| LE_001050 | 801 | 171 | -2.23 | down | glycoside hydrolase family 15 protein | *G. luxurians* FD-317 M1 | 0 | 86.01 |
| LE_008436 | 339 | 72 | -2.23 | down | hypothetical protein GYMLUDRAFT_150312 | *G. luxurians* FD-317 M1 | 0 | 70.38 |
| LE_003193 | 1,045 | 222 | -2.23 | down | Gpi1-domain-containing protein | *C. torrendii* FP15055 ss-10 | 3E-127 | 44.97 |
| LE_003157 | 2,365 | 502 | -2.23 | down | hypothetical protein GYMLUDRAFT_44247 | *G. luxurians* FD-317 M1 | 7E-96 | 50.00 |
| LE_002640 | 915 | 194 | -2.24 | down | hypothetical protein GYMLUDRAFT_66351 | *G. luxurians* FD-317 M1 | 1E-75 | 67.61 |
| LE_001526 | 205 | 43 | -2.24 | down | hypothetical protein GYMLUDRAFT_1021654 | *G. luxurians* FD-317 M1 | 1E-178 | 86.39 |
| LE_009401 | 1,277 | 269 | -2.25 | down | glutathione reductase | *F. hepatica* ATCC 64428 | 0 | 77.17 |
| LE_001110 | 795 | 167 | -2.25 | down | hypothetical protein GYMLUDRAFT_949192 | *G. luxurians* FD-317 M1 | 0 | 73.13 |
| LE_000383 | 1,358 | 284 | -2.25 | down | hypothetical protein GYMLUDRAFT_46518 | *G. luxurians* FD-317 M1 | 1E-147 | 72.30 |
| LE_005055 | 383 | 80 | -2.25 | down |  |  |  |  |
| LE_003695 | 119 | 24 | -2.26 | down | general substrate transporter | *F. mediterranea* MF3/22 | 6E-126 | 46.58 |
| LE_002105 | 112 | 23 | -2.26 | down | MFS general substrate transporter | *F. hepatica* ATCC 64428 | 7E-156 | 66.17 |
| LE_008460 | 101 | 21 | -2.26 | down | hypothetical protein GYMLUDRAFT_45514 | *G. luxurians* FD-317 M1 | 2E-41 | 70.43 |
| LE_000325 | 4,010 | 830 | -2.27 | down | hypothetical protein GYMLUDRAFT_55071 | *G. luxurians* FD-317 M1 | 0 | 69.16 |
| LE_008121 | 1,835 | 380 | -2.27 | down | hypothetical protein GYMLUDRAFT_236189 | *G. luxurians* FD-317 M1 | 2E-145 | 55.70 |
| LE_000470 | 2,558 | 529 | -2.27 | down | hypothetical protein M413DRAFT_61440 | *H. cylindrosporum* h7 | 0 | 57.92 |
| LE_002852 | 2,000 | 413 | -2.27 | down | hypothetical protein GYMLUDRAFT_60493 | *G. luxurians* FD-317 M1 | 2E-119 | 87.80 |
| LE_008092 | 9,022 | 1,862 | -2.28 | down | Negative regulator of sexual conjugation and meiosis | *Termitomyces sp.* J132 | 0 | 57.88 |
| LE_003028 | 3,160 | 652 | -2.28 | down | glycoside hydrolase family 16 protein | *G. luxurians* FD-317 M1 | 0 | 73.38 |
| LE_001159 | 101 | 21 | -2.28 | down | hypothetical protein GYMLUDRAFT_34017 | *G. luxurians* FD-317 M1 | 9E-141 | 71.47 |
| LE_001266 | 1,769 | 363 | -2.28 | down | hypothetical protein GYMLUDRAFT_40223 | *G. luxurians* FD-317 M1 | 0 | 82.81 |
| LE_000225 | 4,932 | 1,010 | -2.29 | down | 6-phosphogluconate dehydrogenase | *A. bisporus var. bisporus* H97 | 0 | 88.55 |
| LE_008227 | 1,288 | 263 | -2.29 | down | Protein disulfide-isomerase tigA | *Termitomyces sp.* J132 | 1E-114 | 63.80 |
| LE_000615 | 151 | 31 | -2.29 | down | Transcriptional regulatory protein pro-1 | *Termitomyces sp*. J132 | 3E-118 | 44.34 |
| LE_008807 | 393 | 80 | -2.29 | down | hypothetical protein GYMLUDRAFT_37608 | *G. luxurians* FD-317 M1 | 0 | 85.25 |
| LE_009205 | 787 | 160 | -2.29 | down | hypothetical protein GYMLUDRAFT_232565 | *G. luxurians* FD-317 M1 | 3E-116 | 73.90 |
| LE_001497 | 409 | 83 | -2.30 | down | carbohydrate esterase family 4 protein | *G. luxurians* FD-317 M1 | 0 | 68.66 |
| LE_008353 | 428 | 86 | -2.30 | down | L-lactate dehydrogenase | *G. trabeum* ATCC 11539 | 0 | 84.84 |
| LE_003323 | 291 | 59 | -2.31 | down | hypothetical protein CERSUDRAFT_119866 | *G. subvermispora* B | 1E-103 | 45.52 |
| LE_001244 | 811 | 164 | -2.31 | down | beta-N-acetylhexosaminidase | *Lentinula edodes* | 0 | 99.64 |
| LE_009106 | 538 | 108 | -2.32 | down |  |  |  |  |
| LE_000289 | 4,782 | 959 | -2.32 | down | acid protease | *C. torrendii* FP15055 ss-10 | 0 | 60.04 |
| LE_003340 | 235 | 47 | -2.32 | down | hypothetical protein GYMLUDRAFT_57310 | *G. luxurians* FD-317 M1 | 4E-38 | 78.76 |
| LE_004434 | 134 | 27 | -2.32 | down | hypothetical protein STEHIDRAFT_165519 | *S. hirsutum* FP-91666 SS1 | 1E-21 | 43.05 |
| LE_002838 | 6,774 | 1,353 | -2.32 | down | hypothetical protein GYMLUDRAFT_238981 | *G. luxurians* FD-317 M1 | 2E-101 | 80.21 |
| LE_002897 | 3,401 | 679 | -2.32 | down | hypothetical protein GYMLUDRAFT_80703 | *G. luxurians* FD-317 M1 | 7E-46 | 38.60 |
| LE_002609 | 808 | 161 | -2.32 | down | hypothetical protein GYMLUDRAFT_33040 | *G. luxurians* FD-317 M1 | 0 | 80.70 |
| LE_009857 | 659 | 131 | -2.33 | down | hypothetical protein GALMADRAFT_252173 | *G. marginata* CBS 339.88 | 6E-32 | 34.20 |
| LE_009471 | 304 | 61 | -2.33 | down | hypothetical protein GYMLUDRAFT_37080 | *G. luxurians* FD-317 M1 | 0 | 89.36 |
| LE_001654 | 590 | 117 | -2.33 | down | Aldose 1-epimerase | *Termitomyces sp.* J132 | 2E-163 | 57.24 |
| LE_008535 | 386 | 76 | -2.33 | down |  |  |  |  |
| LE_002679 | 577 | 114 | -2.34 | down | hypothetical protein GYMLUDRAFT_33711 | *G. luxurians* FD-317 M1 | 5E-116 | 78.14 |
| LE_001481 | 689 | 136 | -2.34 | down | Fructose-bisphosphate aldolase A | *Termitomyces sp.* J132 | 7E-126 | 53.98 |
| LE_008573 | 2,838 | 559 | -2.34 | down | elongation factor 1-gamma | *D. squalens* LYAD-421 SS1 | 3E-174 | 64.56 |
| LE_000276 | 20,464 | 4,021 | -2.35 | down | putative NADPH oxidase | *H. irregulare* TC 32-1 | 0 | 75.73 |
| LE_000728 | 251 | 49 | -2.35 | down | hypothetical protein GYMLUDRAFT_929197 | *G. luxurians* FD-317 M1 | 3E-46 | 42.74 |
| LE_001073 | 3,228 | 633 | -2.35 | down | glycosyltransferase family 32 protein | *G. luxurians* FD-317 M1 | 0 | 82.65 |
| LE_000976 | 2,213 | 433 | -2.35 | down | Cohesin subunit psc3 | *Termitomyces sp.* J132 | 0 | 43.49 |
| LE_002245 | 161 | 31 | -2.35 | down | aspergillopepsin | *D. squalens* LYAD-421 SS1 | 9E-70 | 66.41 |
| LE_008525 | 264 | 51 | -2.35 | down | hypothetical protein GYMLUDRAFT_38158 | *G. luxurians* FD-317 M1 | 0 | 59.03 |
| LE_010266 | 300 | 58 | -2.35 | down | hypothetical protein GYMLUDRAFT_722020 | *G. luxurians* FD-317 M1 | 1E-162 | 71.55 |
| LE_003062 | 365 | 71 | -2.36 | down | glycoside hydrolase family 23 protein | *G. luxurians* FD-317 M1 | 1E-140 | 67.28 |
| LE_001015 | 1,556 | 303 | -2.36 | down | metallopeptidase MepB | *C. cinerea* okayama7#130 | 0 | 78.72 |
| LE_003201 | 164 | 31 | -2.36 | down | hypothetical protein GYMLUDRAFT_241440 | *G. luxurians* FD-317 M1 | 6E-103 | 49.08 |
| LE_009206 | 3,274 | 635 | -2.37 | down | hypothetical protein GYMLUDRAFT_772062 | *G. luxurians* FD-317 M1 | 6E-53 | 66.89 |
| LE_000297 | 3,005 | 582 | -2.37 | down | hypothetical protein GYMLUDRAFT_78128 | *G. luxurians* FD-317 M1 | 7E-93 | 67.61 |
| LE_001157 | 2,263 | 438 | -2.37 | down | hypothetical protein GYMLUDRAFT_34828 | *G. luxurians* FD-317 M1 | 0 | 76.68 |
| LE_000657 | 1,649 | 318 | -2.38 | down | hypothetical protein GYMLUDRAFT_206131 | *G. luxurians* FD-317 M1 | 0 | 69.59 |
| LE_002819 | 2,252 | 433 | -2.38 | down | hypothetical protein GYMLUDRAFT_56777 | *G. luxurians* FD-317 M1 | 0 | 64.03 |
| LE_008298 | 203 | 39 | -2.38 | down | similar to competence/damage-inducible protein cinA | *L. maculans* JN3 | 1E-37 | 49.66 |
| LE_009180 | 212 | 40 | -2.38 | down | hypothetical protein GYMLUDRAFT_35069 | *G. luxurians* FD-317 M1 | 0 | 76.84 |
| LE_000269 | 812 | 155 | -2.39 | down | hypothetical protein GYMLUDRAFT_51955 | *G. luxurians* FD-317 M1 | 3E-146 | 61.96 |
| LE_008462 | 235 | 44 | -2.39 | down | S-adenosylmethionine decarboxylase proenzyme | *Termitomyces sp.* J132 | 2E-137 | 60.65 |
| LE_009314 | 106 | 20 | -2.39 | down | NmrA-like family domain-containing protein 1 | *Termitomyces sp.* J132 | 2E-138 | 58.02 |
| LE_009264 | 708 | 135 | -2.39 | down |  |  |  |  |
| LE_009218 | 4,163 | 792 | -2.39 | down | MFS general substrate transporter | *S. hirsutum* FP-91666 SS1 | 0 | 65.44 |
| LE_000618 | 2,482 | 471 | -2.40 | down | hypothetical protein GYMLUDRAFT_33464, partial | *G. luxurians* FD-317 M1 | 8E-86 | 87.07 |
| LE_008288 | 1,027 | 195 | -2.40 | down | hypothetical protein GYMLUDRAFT_173135 | *G. luxurians* FD-317 M1 | 0 | 89.23 |
| LE_000284 | 1,924 | 365 | -2.40 | down | hypothetical protein GYMLUDRAFT_46373 | *G. luxurians* FD-317 M1 | 0 | 75.03 |
| LE_008113 | 6,526 | 1,236 | -2.40 | down | DUF1929-domain-containing protein | *G. trabeum* ATCC 11539 | 0 | 73.56 |
| LE_001263 | 1,015 | 192 | -2.40 | down | Nucleolar essential protein 1 | *Termitomyces sp.* J132 | 2E-129 | 72.51 |
| LE_000613 | 395 | 74 | -2.40 | down | hypothetical protein GYMLUDRAFT_252190 | *G. luxurians* FD-317 M1 | 2E-165 | 59.78 |
| LE_001918 | 2,896 | 547 | -2.40 | down | glycoside hydrolase family 13 protein | *G. luxurians* FD-317 M1 | 0 | 77.84 |
| LE_001357 | 293 | 55 | -2.41 | down | hypothetical protein GYMLUDRAFT_33520 | *G. luxurians* FD-317 M1 | 3E-142 | 66.03 |
| LE_008109 | 3,557 | 669 | -2.41 | down | hypothetical protein GYMLUDRAFT_76493 | *G. luxurians* FD-317 M1 | 2E-32 | 61.17 |
| LE_008828 | 164 | 31 | -2.41 | down | glycoside hydrolase family 78 protein | *G. luxurians* FD-317 M1 | 0 | 80.58 |
| LE_009251 | 1,453 | 273 | -2.41 | down | beta-flanking protein | *L. edodes* | 8E-84 | 76.86 |
| LE_009112 | 1,493 | 280 | -2.41 | down | voltage-gated potassium channel beta-2 subunit | *C. cinerea* okayama7#130 | 0 | 77.3 |
| LE_004072 | 397 | 74 | -2.41 | down | NAD-dependent formate dehydrogenase | *C. torrendii* FP15055 ss-10 | 0 | 84.08 |
| LE_002888 | 9,686 | 1,817 | -2.41 | down | hypothetical protein CYLTODRAFT_404673 | *C. torrendii* FP15055 ss-10 | 2E-63 | 50.54 |
| LE_002433 | 1,753 | 328 | -2.41 | down | hypothetical protein GYMLUDRAFT_36321 | *G. luxurians* FD-317 M1 | 3E-140 | 75.23 |
| LE_008405 | 224 | 42 | -2.42 | down | NADH:flavin oxidoreductase / NADH oxidase | *L. edodes* | 0 | 98.76 |
| LE_003314 | 158 | 29 | -2.42 | down | hypothetical protein M413DRAFT_373097 | *H cylindrosporum* h7 | 3E-63 | 39.08 |
| LE_000669 | 3,702 | 692 | -2.42 | down | endoplasmic reticulum-derived transport vesicle ERV46 | *L bicolor* S238N-H82 | 0 | 76.50 |
| LE_002316 | 796 | 148 | -2.42 | down | hypothetical protein GYMLUDRAFT_72080 | *G. luxurians* FD-317 M1 | 2E-116 | 46.47 |
| LE_008292 | 790 | 147 | -2.42 | down | xyloglucan-specific endoglucanase | *L. edodes* | 3E-139 | 99.61 |
| LE_000858 | 1,389 | 258 | -2.43 | down | hypothetical protein SERLA73DRAFT_116909 | *S lacrymans var. lacrymans* S7.3 | 0 | 53.66 |
| LE_000419 | 3,624 | 672 | -2.43 | down | glycoside hydrolase | *F. mediterranea* MF3/22 | 3E-125 | 68.28 |
| LE_003512 | 200 | 37 | -2.43 | down | hypothetical protein GYMLUDRAFT_737151 | *G. luxurians* FD-317 M1 | 0 | 68.57 |
| LE_009329 | 281 | 52 | -2.44 | down | hypothetical protein GYMLUDRAFT_256950 | *G. luxurians* FD-317 M1 | 9E-81 | 70.65 |
| LE_000589 | 2,510 | 463 | -2.44 | down | glycosyltransferase family 20 protein | *G. luxurians* FD-317 M1 | 0 | 73.62 |
| LE_008127 | 1,018 | 187 | -2.44 | down | glycoside hydrolase family 5 protein | *G. luxurians* FD-317 M1 | 9E-167 | 85.53 |
| LE_001520 | 738 | 136 | -2.44 | down | Putative aminodeoxychorismate synthase | *Termitomyces sp.* J132 | 0 | 58.78 |
| LE_008892 | 127 | 23 | -2.45 | down | hypothetical protein GYMLUDRAFT_76827 | *G. luxurians* FD-317 M1 | 7E-137 | 43.72 |
| LE_003400 | 394 | 72 | -2.45 | down |  |  |  |  |
| LE_000193 | 4,379 | 801 | -2.45 | down | hypothetical protein GYMLUDRAFT_37455 | *G. luxurians* FD-317 M1 | 2E-118 | 71.90 |
| LE_003163 | 388 | 71 | -2.45 | down | hypothetical protein GYMLUDRAFT_241307 | *G. luxurians* FD-317 M1 | 0 | 68.40 |
| LE_003160 | 303 | 55 | -2.45 | down |  |  |  |  |
| LE_001299 | 302 | 55 | -2.46 | down | molybdenum cofactor biosynthesis prote | *C. torrendii* FP15055 ss-10 | 1E-78 | 55.51 |
| LE_001338 | 1,176 | 214 | -2.46 | down | iron reductase | *T. versicolor* FP-101664 SS1 | 0 | 56.13 |
| LE_001381 | 1,482 | 268 | -2.46 | down | hypothetical protein GYMLUDRAFT_172229 | *G. luxurians* FD-317 M1 | 0 | 81.76 |
| LE_004581 | 1,189 | 215 | -2.46 | down | hypothetical protein GYMLUDRAFT_168967 | *G. luxurians* FD-317 M1 | 0 | 80.33 |
| LE_001069 | 1,601 | 289 | -2.47 | down | hypothetical protein GYMLUDRAFT_53443 | *G. luxurians* FD-317 M1 | 0 | 78.77 |
| LE_003088 | 275 | 49 | -2.47 | down | hypothetical protein GYMLUDRAFT_175576 | *G. luxurians* FD-317 M1 | 1E-171 | 77.23 |
| LE_002228 | 178 | 32 | -2.47 | down | hypothetical protein GYMLUDRAFT_976369 | *G. luxurians* FD-317 M1 | 2E-48 | 61.87 |
| LE_000529 | 2,043 | 369 | -2.47 | down | cytochrome P450 | *P. placenta* | 2E-145 | 41.97 |
| LE_000895 | 767 | 138 | -2.47 | down | peptidase C1B bleomycin hydrolase | *C. torrendii* FP15055 ss-10 | 0 | 67.50 |
| LE_003191 | 668 | 120 | -2.47 | down | MFS general substrate transporter | *D.* *squalens* LYAD-421 SS1 | 0 | 74.22 |
| LE_009109 | 941 | 169 | -2.47 | down | hypothetical protein GYMLUDRAFT_147950 | *G. luxurians* FD-317 M1 | 2E-117 | 50.55 |
| LE_000558 | 142 | 25 | -2.47 | down | hypothetical protein GYMLUDRAFT_249195 | *G. luxurians* FD-317 M1 | 8E-43 | 35.72 |
| LE_009465 | 376 | 67 | -2.47 | down | hypothetical protein M422DRAFT_52454 | *S. stellatus* SS14 | 5E-24 | 32.11 |
| LE_005982 | 385 | 69 | -2.47 | down | aldo/keto reductase | *C. cinerea* okayama7#130 | 3E-175 | 71.68 |
| LE_009325 | 112 | 20 | -2.48 | down | high affinity methionine permease | *C. cinerea* okayama7#130 | 0 | 56.03 |
| LE_000820 | 1,675 | 299 | -2.48 | down |  |  |  |  |
| LE_008308 | 342 | 61 | -2.48 | down | hypothetical protein GYMLUDRAFT_33420 | *G. luxurians* FD-317 M1 | 0 | 83.75 |
| LE_000152 | 266 | 47 | -2.49 | down | maltose permease | *P. strigosozonata* HHB-11173 SS5 | 0 | 78.26 |
| LE_002963 | 191 | 34 | -2.49 | down | Arginase/deacetylase | *C. torrendii* FP15055 ss-10 | 0 | 77.78 |
| LE_000142 | 30,225 | 5,368 | -2.49 | down | hypothetical protein GYMLUDRAFT_41410 | *G. luxurians* FD-317 M1 | 2E-92 | 65.94 |
| LE_003534 | 368 | 65 | -2.50 | down | transcriptional regulator | *B. bassiana* ARSEF 2860 | 3E-152 | 56.99 |
| LE_003573 | 276 | 49 | -2.50 | down | hypothetical protein GYMLUDRAFT_67179 | *G. luxurians* FD-317 M1 | 7E-68 | 46.74 |
| LE_000515 | 1,857 | 328 | -2.50 | down | Lipase 1 | *Termitomyces sp.* J132 | 0 | 58.40 |
| LE_000620 | 1,243 | 219 | -2.50 | down | glycoside hydrolase family 5 protein | *G. luxurians* FD-317 M1 | 0 | 90.32 |
| LE_000429 | 1,630 | 287 | -2.50 | down |  |  |  |  |
| LE_008315 | 3,651 | 639 | -2.51 | down | hypothetical protein GYMLUDRAFT_192853 | *G. luxurians* FD-317 M1 | 2E-119 | 74.09 |
| LE_000489 | 4,637 | 811 | -2.51 | down | carbohydrate-binding module family 67 protein | *C.* *torrendii* FP15055 ss-10 | 1E-95 | 54.42 |
| LE_000171 | 3,026 | 529 | -2.52 | down | hypothetical protein SERLA73DRAFT_144458 | *S. lacrymans var. lacrymans* S7.3 | 0 | 89.81 |
| LE_002557 | 1,717 | 300 | -2.52 | down |  |  |  |  |
| LE_008549 | 703 | 122 | -2.52 | down | hypothetical protein GYMLUDRAFT_237225 | *G. luxurians* FD-317 M1 | 0 | 79.45 |
| LE_001151 | 1,215 | 211 | -2.53 | down | arylsulfatase | *F. hepatica* ATCC 64428 | 0 | 66.67 |
| LE_003135 | 146 | 25 | -2.53 | down | hypothetical protein GYMLUDRAFT_230308 | *G. luxurians* FD-317 M1 | 5E-165 | 75.97 |
| LE_002948 | 5,284 | 914 | -2.53 | down | hypothetical protein GYMLUDRAFT_81752 | *G. luxurians* FD-317 M1 | 2E-125 | 68.07 |
| LE_002061 | 200 | 34 | -2.53 | down | hypothetical protein GYMLUDRAFT_1021879 | *G. luxurians* FD-317 M1 | 9E-146 | 61.11 |
| LE_008488 | 436 | 75 | -2.54 | down | hypothetical protein GYMLUDRAFT_180686 | *G. luxurians* FD-317 M1 | 0 | 81.69 |
| LE_002548 | 370 | 63 | -2.54 | down | hypothetical protein GYMLUDRAFT_33103 | *G. luxurians* FD-317 M1 | 3E-143 | 71.28 |
| LE_009282 | 138 | 24 | -2.54 | down |  |  |  |  |
| LE_002499 | 212 | 36 | -2.54 | down | hypothetical protein GYMLUDRAFT_47764 | *G. luxurians* FD-317 M1 | 0 | 74.92 |
| LE_010413 | 422 | 72 | -2.54 | down | glycoside hydrolase family 5 protein | *G. luxurians* FD-317 M1 | 0 | 79.82 |
| LE_008125 | 547 | 93 | -2.55 | down | hypothetical protein GYMLUDRAFT_35336 | *G. luxurians* FD-317 M1 | 2E-160 | 70.57 |
| LE_000382 | 10,280 | 1,755 | -2.55 | down | Catalase | *Termitomyces sp.* J132 | 0 | 83.14 |
| LE_002953 | 159 | 27 | -2.55 | down |  |  |  |  |
| LE_003104 | 555 | 93 | -2.56 | down | NAD-P-binding protein | *S. hirsutum* FP-91666 SS1 | 7E-76 | 41.92 |
| LE_000420 | 1,250 | 211 | -2.56 | down | hypothetical protein GYMLUDRAFT_260511 | *G. luxurians* FD-317 M1 | 4E-58 | 85.71 |
| LE_000407 | 1,843 | 311 | -2.57 | down | hypothetical protein GYMLUDRAFT_237737 | *G. luxurians* FD-317 M1 | 3E-80 | 39.54 |
| LE_004015 | 247 | 41 | -2.57 | down | hypothetical protein GYMLUDRAFT_78050 | *G. luxurians* FD-317 M1 | 7E-166 | 83.72 |
| LE_000593 | 4,508 | 760 | -2.57 | down | GMC oxidoreductase | *C. torrendii* FP15055 ss-10 | 4E-179 | 48.76 |
| LE_002087 | 145 | 24 | -2.57 | down | hypothetical protein GYMLUDRAFT_53107 | *G. luxurians* FD-317 M1 | 0 | 72.33 |
| LE_000661 | 647 | 108 | -2.58 | down | glycoside hydrolase family 13 protein | *G. luxurians* FD-317 M1 | 0 | 81.62 |
| LE_001830 | 236 | 39 | -2.58 | down | hypothetical protein GYMLUDRAFT_158851 | *G. luxurians* FD-317 M1 | 4E-115 | 75.43 |
| LE_009533 | 467 | 78 | -2.58 | down | hypothetical protein GYMLUDRAFT_203254 | *G. luxurians* FD-317 M1 | 0 | 74.30 |
| LE_000172 | 34,407 | 5,731 | -2.59 | down | putative aspartic-type endopeptidase CTSD | *Termitomyces sp.* J132 | 1E-169 | 75.36 |
| LE_001318 | 487 | 81 | -2.59 | down | carbohydrate esterase family 1 protein | *G. luxurians* FD-317 M1 | 0 | 88.11 |
| LE_003418 | 343 | 57 | -2.59 | down | hypothetical protein GYMLUDRAFT_40149 | *G. luxurians* FD-317 M1 | 0 | 71.61 |
| LE_002278 | 439 | 73 | -2.59 | down | hypothetical protein GYMLUDRAFT_67275 | *G. luxurians* FD-317 M1 | 2E-23 | 37.79 |
| LE_005080 | 2,645 | 438 | -2.59 | down |  |  |  |  |
| LE_000742 | 937 | 155 | -2.59 | down | MFS general substrate transporter, partial | *C. torrendii* FP15055 ss-10 | 6E-161 | 51.47 |
| LE_009716 | 673 | 111 | -2.59 | down | general substrate transporter | *D. squalens* LYAD-421 SS1 | 3E-82 | 58.06 |
| LE_008381 | 156 | 26 | -2.60 | down | MFS general substrate transporter | *A. subglabra* TFB-10046 SS5 | 0 | 71.40 |
| LE_001368 | 1,648 | 272 | -2.60 | down | hypothetical protein GYMLUDRAFT_194708 | *G. luxurians* FD-317 M1 | 3E-153 | 81.19 |
| LE_000509 | 262 | 43 | -2.60 | down | aldolase | *S. hirsutum* FP-91666 SS1 | 2E-120 | 55.22 |
| LE_000401 | 9,502 | 1,562 | -2.60 | down | hypothetical protein GYMLUDRAFT_33531 | *G. luxurians* FD-317 M1 | 0E+00 | 85.19 |
| LE_008571 | 150 | 24 | -2.61 | down | MFS general substrate transporter | *C. torrendii* FP15055 ss-10 | 0 | 65.05 |
| LE_003565 | 237 | 38 | -2.62 | down | NicO-domain-containing protein | *C. torrendii* FP15055 ss-10 | 7E-140 | 55.86 |
| LE_001392 | 704 | 114 | -2.62 | down | hypothetical protein GYMLUDRAFT_33768 | *G. luxurians* FD-317 M1 | 0 | 91.46 |
| LE_000753 | 348 | 56 | -2.62 | down |  |  |  |  |
| LE_001677 | 1,492 | 242 | -2.62 | down | Putative acyl-CoA dehydrogenase AidB | *Termitomyces sp.* J132 | 0 | 63.15 |
| LE_009104 | 466 | 76 | -2.62 | down | hypothetical protein GYMLUDRAFT_45535 | *G. luxurians* FD-317 M1 | 0 | 52.62 |
| LE_000938 | 1,030 | 167 | -2.62 | down | hypothetical protein GYMLUDRAFT_207500 | *G. luxurians* FD-317 M1 | 0 | 84.07 |
| LE_000363 | 3,585 | 580 | -2.63 | down | glucose-6-phosphate 1-dehydrogenase | *V. volvacea* | 0 | 88.74 |
| LE_002075 | 2,515 | 406 | -2.63 | down | exo-beta-1,3-glucanase | *L. edodes* | 0 | 98.57 |
| LE_001247 | 129 | 20 | -2.64 | down | hypothetical protein GYMLUDRAFT_241128 | *G. luxurians* FD-317 M1 | 2E-132 | 69.47 |
| LE_000252 | 8,433 | 1,355 | -2.64 | down | hypothetical protein GYMLUDRAFT_34934 | *G. luxurians* FD-317 M1 | 0 | 71.37 |
| LE_008356 | 439 | 70 | -2.64 | down | hypothetical protein GYMLUDRAFT_215812 | *G. luxurians* FD-317 M1 | 0 | 67.45 |
| LE_000365 | 11,588 | 1,859 | -2.64 | down | Nuclear fusion protein FUS1 | *Termitomyces sp.* J132 | 2E-16 | 50.00 |
| LE_001867 | 1,580 | 251 | -2.65 | down | hypothetical protein GYMLUDRAFT_39797 | *G. luxurians* FD-317 M1 | 0 | 70.41 |
| LE_002721 | 117 | 18 | -2.66 | down | hypothetical protein GYMLUDRAFT_249957 | *G. luxurians* FD-317 M1 | 0 | 68.20 |
| LE_005313 | 192 | 30 | -2.66 | down | hypothetical protein GYMLUDRAFT_158649 | *G. luxurians* FD-317 M1 | 0 | 77.80 |
| LE_000481 | 2,757 | 435 | -2.66 | down | transmembrane protein, putative | *R. solani* AG-3 Rhs1AP | 6E-61 | 43.56 |
| LE_001429 | 145 | 23 | -2.67 | down | hypothetical protein GYMLUDRAFT_208428 | *G. luxurians* FD-317 M1 | 0 | 74.16 |
| LE_008164 | 366 | 56 | -2.68 | down | hypothetical protein GYMLUDRAFT_245962 | *G. luxurians* FD-317 M1 | 4E-40 | 51.69 |
| LE_008464 | 1,302 | 203 | -2.68 | down | hypothetical protein GYMLUDRAFT_243417 | *G. luxurians* FD-317 M1 | 0 | 64.02 |
| LE_000350 | 12,876 | 1,996 | -2.69 | down | hypothetical protein GYMLUDRAFT_904371 | *G. luxurians* FD-317 M1 | 2E-144 | 82.91 |
| LE_002846 | 141 | 22 | -2.69 | down |  |  |  |  |
| LE_001369 | 1,523 | 234 | -2.70 | down | hypothetical protein GYMLUDRAFT_96488 | *G. luxurians* FD-317 M1 | 2E-126 | 76.92 |
| LE_006096 | 564 | 86 | -2.70 | down | K(+)/H(+) antiporter 1 | *Termitomyces sp.* J132 | 0 | 55.08 |
| LE_002996 | 366 | 56 | -2.70 | down | putative chitosanase | *L. edodes* | 3E-167 | 99.29 |
| LE_006258 | 174 | 26 | -2.70 | down | hypothetical protein GYMLUDRAFT_93827 | *G. luxurians* FD-317 M1 | 3E-88 | 68.15 |
| LE_002217 | 466 | 71 | -2.71 | down | Pc12g08300 | *P. rubens Wisconsin* 54-1255 | 0 | 56.06 |
| LE_000823 | 1,037 | 158 | -2.71 | down | hypothetical protein GYMLUDRAFT_246220 | *G. luxurians* FD-317 M1 | 4E-135 | 78.47 |
| LE_008208 | 1,109 | 168 | -2.72 | down | hypothetical protein GYMLUDRAFT_49929 | *G. luxurians* FD-317 M1 | 5E-48 | 63.98 |
| LE_000923 | 887 | 134 | -2.72 | down | NAD-dependent epimerase/dehydratase | *R. toruloides* NP11 | 4E-69 | 42.11 |
| LE_009299 | 201 | 30 | -2.73 | down | hypothetical protein GYMLUDRAFT_39206 | *G. luxurians* FD-317 M1 | 0 | 80.56 |
| LE_002248 | 827 | 124 | -2.74 | down | hypothetical protein GYMLUDRAFT_219036 | *G. luxurians* FD-317 M1 | 0 | 51.47 |
| LE_009819 | 2,489 | 371 | -2.74 | down | hypothetical protein GYMLUDRAFT_36920 | *G. luxurians* FD-317 M1 | 3E-133 | 78.35 |
| LE_006025 | 106 | 15 | -2.74 | down | cytochrome P450 | *G. trabeum* ATCC 11539 | 8E-106 | 41.12 |
| LE_008583 | 702 | 104 | -2.75 | down | TPA_exp: reverse transcriptase/ribonuclease H | *Coprinopsis cinerea* | 9E-168 | 44.43 |
| LE_008368 | 448 | 66 | -2.75 | down | hypothetical protein GYMLUDRAFT_409790 | *G. luxurians* FD-317 M1 | 0 | 61.44 |
| LE_009348 | 840 | 124 | -2.76 | down | hypothetical protein GYMLUDRAFT_81478 | *G. luxurians* FD-317 M1 | 1E-22 | 43.04 |
| LE_003516 | 174 | 25 | -2.76 | down | hypothetical protein GYMLUDRAFT_220361 | *G. luxurians* FD-317 M1 | 0E+00 | 77.22 |
| LE_000413 | 2,594 | 382 | -2.76 | down | Serine/threonine-protein kinase gad8 | *Termitomyces sp.* J132 | 0 | 81.74 |
| LE_000884 | 823 | 120 | -2.77 | down | anthranilate phosphoribosyltransferase, TrpD | *L. bicolor* S238N-H82 | 2E-164 | 76.10 |
| LE_008116 | 399 | 58 | -2.77 | down | DUF706-domain-containing protein | *G. trabeum* ATCC 11539 | 0 | 82.61 |
| LE_008693 | 606 | 88 | -2.77 | down | hypothetical protein GYMLUDRAFT_34056 | *G. luxurians* FD-317 M1 | 0E+00 | 88.18 |
| LE_003116 | 178 | 26 | -2.78 | down | Golgi apparatus membrane protein TVP38 | *Termitomyces sp.* J132 | 2E-87 | 49.65 |
| LE_001226 | 341 | 49 | -2.78 | down | xylitol dehydrogenase | *C. cinerea okayama*7#130 | 2E-172 | 73.00 |
| LE_008186 | 802 | 116 | -2.78 | down | hypothetical protein GYMLUDRAFT_36907 | *G. luxurians* FD-317 M1 | 1E-155 | 79.26 |
| LE_000777 | 5,551 | 807 | -2.78 | down | hypothetical protein GYMLUDRAFT_84731 | *G. luxurians* FD-317 M1 | 0 | 80.07 |
| LE_000739 | 4,911 | 713 | -2.78 | down | hypothetical protein PHLGIDRAFT_19596 | *P. gigantea* 11061_1 CR5-6 | 4E-32 | 84.52 |
| LE_008122 | 2,862 | 416 | -2.78 | down | thioredoxin | *L. bicolor* S238N-H82 | 3E-44 | 69.52 |
| LE_000812 | 599 | 87 | -2.78 | down | hypothetical protein GYMLUDRAFT_90173 | *G. luxurians* FD-317 M1 | 0 | 92.36 |
| LE_003187 | 136 | 19 | -2.79 | down | PAP2-domain-containing protein | *C. torrendii* FP15055 ss-10 | 2E-94 | 58.63 |
| LE_008508 | 810 | 116 | -2.80 | down | carbohydrate-binding module family 35 protein | *G. luxurians* FD-317 M1 | 0 | 84.51 |
| LE_008742 | 199 | 28 | -2.81 | down |  |  |  |  |
| LE_001410 | 337 | 48 | -2.81 | down | glycosyltransferase family 90 protein | *G. luxurians* FD-317 M1 | 0 | 71.63 |
| LE_003382 | 122 | 17 | -2.81 | down | 2-amino-3-carboxymuconate-6-semialdehyde decarboxylase | *B. bassiana* ARSEF 2860 | 3E-133 | 59.87 |
| LE_000781 | 3,321 | 470 | -2.82 | down | peptidyl-prolyl cis-trans isomerase | *S. hirsutum* FP-91666 SS1 | 8E-56 | 82.24 |
| LE_000783 | 563 | 79 | -2.82 | down | hypothetical protein GYMLUDRAFT_74583 | *G. luxurians* FD-317 M1 | 4E-89 | 60.48 |
| LE_009868 | 106 | 15 | -2.83 | down | hypothetical protein AGABI2DRAFT_122905 | *A. bisporus var. bisporus* H97 | 9E-103 | 29.41 |
| LE_008761 | 124 | 17 | -2.83 | down | delta-12 fatty acid desaturase | *P. nameko* | 0 | 58.23 |
| LE_010080 | 226 | 31 | -2.83 | down | hypothetical protein GYMLUDRAFT_36596 | *G. luxurians* FD-317 M1 | 9E-151 | 73.78 |
| LE_008236 | 1,761 | 247 | -2.83 | down | saccharopine dehydrogenase | *L. bicolor* S238N-H82 | 0 | 73.04 |
| LE_000804 | 711 | 99 | -2.84 | down | septin family protein, P-loop GTPase | *L. bicolor* S238N-H82 | 0 | 62.70 |
| LE_002808 | 1,200 | 166 | -2.85 | down | hypothetical protein GYMLUDRAFT_158649 | *G. luxurians* FD-317 M1 | 0 | 74.23 |
| LE_008787 | 405 | 56 | -2.85 | down | hypothetical protein GYMLUDRAFT_46358 | *G. luxurians* FD-317 M1 | 0 | 50.20 |
| LE_009041 | 340 | 47 | -2.85 | down | argonaute-like protein | *L. bicolor* S238N-H82 | 0 | 49.83 |
| LE_005113 | 256 | 35 | -2.86 | down | hypothetical protein GYMLUDRAFT_39491 | *G. luxurians* FD-317 M1 | 0 | 91.20 |
| LE_001141 | 384 | 53 | -2.86 | down | oxidoreductase | *F. hepatica* ATCC 64428 | 1E-178 | 60.71 |
| LE_000909 | 457 | 63 | -2.86 | down | hypothetical protein GYMLUDRAFT_35301 | *G. luxurians* FD-317 M1 | 0 | 75.51 |
| LE_003402 | 119 | 16 | -2.86 | down | hypothetical protein GYMLUDRAFT_71407 | *G. luxurians* FD-317 M1 | 1E-124 | 57.84 |
| LE_008211 | 349 | 48 | -2.87 | down | hypothetical protein GALMADRAFT_1138844 | *G. marginata* CBS 339.88 | 5E-11 | 23.67 |
| LE_000828 | 960 | 131 | -2.87 | down | glycoside hydrolase family 92 protein | *G. luxurians* FD-317 M1 | 0E+00 | 87.72 |
| LE_003701 | 185 | 25 | -2.87 | down | hypothetical protein GYMLUDRAFT_238041 | *G. luxurians* FD-317 M1 | 0E+00 | 74.44 |
| LE_002437 | 110 | 15 | -2.87 | down | RTA-like protein | *R. toruloides* NP11 | 1E-75 | 50.52 |
| LE_010342 | 327 | 44 | -2.88 | down |  |  |  |  |
| LE_002847 | 2,819 | 380 | -2.89 | down | hypothetical protein GYMLUDRAFT_917680 | *G. luxurians* FD-317 M1 | 1E-116 | 57.14 |
| LE_000476 | 5,163 | 695 | -2.89 | down | hypothetical protein GYMLUDRAFT_92458 | *G. luxurians* FD-317 M1 | 0 | 80.00 |
| LE_001880 | 2,006 | 267 | -2.91 | down | hypothetical protein GYMLUDRAFT_259693 | *G. luxurians* FD-317 M1 | 0 | 65.60 |
| LE_003155 | 416 | 55 | -2.91 | down | voltage-gated potassium channel beta-2 subunit | *C. cinerea okayama*7#130 | 0 | 75.14 |
| LE_001213 | 668 | 89 | -2.91 | down | hypothetical protein GYMLUDRAFT_58439 | *G. luxurians* FD-317 M1 | 2E-15 | 36.47 |
| LE_003228 | 498 | 66 | -2.91 | down | hypothetical protein GYMLUDRAFT_65871 | *G. luxurians* FD-317 M1 | 0 | 73.73 |
| LE_003052 | 164 | 21 | -2.91 | down | PLC-like phosphodiesterase | *T. versicolor* FP-101664 SS1 | 3E-148 | 67.96 |
| LE_008363 | 458 | 60 | -2.92 | down | hypothetical protein GYMLUDRAFT_37285 | *G. luxurians* FD-317 M1 | 0 | 79.38 |
| LE_008398 | 192 | 25 | -2.93 | down | hypothetical protein GYMLUDRAFT_174366 | *G. luxurians* FD-317 M1 | 6E-113 | 79.72 |
| LE_003670 | 262 | 34 | -2.93 | down | hypothetical protein GYMLUDRAFT_48291 | *G. luxurians* FD-317 M1 | 0 | 77.45 |
| LE_008111 | 3,997 | 519 | -2.94 | down |  |  |  |  |
| LE_001759 | 1,262 | 163 | -2.95 | down |  |  |  |  |
| LE_002314 | 397 | 51 | -2.95 | down | alcohol oxidase | *S. hirsutum* FP-91666 SS1 | 0 | 62.84 |
| LE_008526 | 699 | 90 | -2.95 | down | hypothetical protein GYMLUDRAFT_32497 | *G. luxurians* FD-317 M1 | 0 | 77.43 |
| LE_000906 | 230 | 29 | -2.96 | down | carbohydrate esterase family 8 protein | *G. luxurians* FD-317 M1 | 4E-157 | 81.60 |
| LE_008120 | 2,251 | 290 | -2.96 | down | glycosyltransferase family 5 protein | *G. luxurians* FD-317 M1 | 0 | 87.33 |
| LE_000677 | 688 | 88 | -2.96 | down | hypothetical protein GYMLUDRAFT_48883 | *G. luxurians* FD-317 M1 | 4E-73 | 68.86 |
| LE_001475 | 2,808 | 361 | -2.96 | down | Aldo/keto reductase | *G. trabeum* ATCC 11539 | 5E-179 | 74.85 |
| LE_000187 | 34,203 | 4,372 | -2.97 | down | hypothetical protein GYMLUDRAFT_37213 | *G. luxurians* FD-317 M1 | 4E-59 | 76.92 |
| LE_005075 | 415 | 53 | -2.97 | down | hypothetical protein GYMLUDRAFT_34628 | *G. luxurians* FD-317 M1 | 0 | 59.45 |
| LE_001466 | 3,538 | 451 | -2.97 | down |  |  |  |  |
| LE_002339 | 374 | 47 | -2.97 | down | hypothetical protein GYMLUDRAFT_234117 | *G. luxurians* FD-317 M1 | 2E-80 | 74.01 |
| LE_001595 | 159 | 20 | -2.97 | down | cyclase family protein | *Dacryopinax sp.* DJM-731 SS1 | 3E-58 | 47.71 |
| LE_008195 | 761 | 97 | -2.97 | down | hypothetical protein GYMLUDRAFT_241840 | *G. luxurians* FD-317 M1 | 6E-98 | 52.14 |
| LE_000377 | 2,053 | 261 | -2.97 | down | hypothetical protein GYMLUDRAFT_41727 | *G. luxurians* FD-317 M1 | 1E-46 | 83.89 |
| LE_001648 | 970 | 123 | -2.98 | down | hypothetical protein GYMLUDRAFT_39025 | *G. luxurians* FD-317 M1 | 9E-172 | 85.67 |
| LE_002177 | 964 | 122 | -2.98 | down | FMN-linked oxidoreductase | *P. strigosozonata* HHB-11173 SS5 | 6E-107 | 56.29 |
| LE_003439 | 154 | 19 | -2.99 | down | hypothetical protein GALMADRAFT_239970 | *G. marginata* CBS 339.88 | 8E-115 | 55.35 |
| LE_000129 | 3,495 | 439 | -2.99 | down | PLC-like phosphodiesterase | *T. versicolor* FP-101664 SS1 | 1E-133 | 60.60 |
| LE_008513 | 780 | 97 | -3.00 | down | amine oxidase catalytic domain-containing protein | *C. torrendii* FP15055 ss-10 | 0 | 64.64 |
| LE_001088 | 333 | 41 | -3.00 | down | phosphoadenosine phosphosulfate reductase | *Termitomyces sp.* J132 | 4E-138 | 79.17 |
| LE_003244 | 485 | 60 | -3.01 | down | enoyl-CoA hydratase 2 | *Termitomyces sp.* J132 | 9E-154 | 70.55 |
| LE_004269 | 124 | 15 | -3.01 | down | hypothetical protein GYMLUDRAFT_233276 | *G. luxurians* FD-317 M1 | 0 | 79.95 |
| LE_000126 | 1,341 | 166 | -3.01 | down | aldo/keto reductase | *C. torrendii* FP15055 ss-10 | 2E-111 | 59.31 |
| LE_003529 | 124 | 15 | -3.02 | down | MFS transporter | *S. hirsutum* FP-91666 SS1 | 0 | 68.76 |
| LE_008877 | 216 | 26 | -3.02 | down |  |  |  |  |
| LE_004064 | 1,326 | 163 | -3.02 | down | hypothetical protein GYMLUDRAFT_175753 | *G. luxurians* FD-317 M1 | 0 | 75.57 |
| LE_000159 | 6,579 | 814 | -3.02 | down | kinesin protein | *L. bicolor* S238N-H82 | 0 | 61.52 |
| LE_000440 | 155 | 19 | -3.03 | down |  |  |  |  |
| LE_009340 | 219 | 26 | -3.04 | down | glycosyltransferase family 15 protein | *G. luxurians* FD-317 M1 | 4E-152 | 64.13 |
| LE_000280 | 3,619 | 441 | -3.04 | down | hypothetical protein GYMLUDRAFT_33341 | *G. luxurians* FD-317 M1 | 5E-95 | 70.45 |
| LE_008306 | 834 | 101 | -3.05 | down | glycoside hydrolase family 43 protein | *G. luxurians* FD-317 M1 | 6E-148 | 76.70 |
| LE_000288 | 7,760 | 939 | -3.05 | down | hypothetical protein GYMLUDRAFT_47410 | *G. luxurians* FD-317 M1 | 0 | 67.72 |
| LE_008095 | 1,238 | 148 | -3.06 | down | cytochrome P450 | *S. hirsutum* FP-91666 SS1 | 1E-130 | 44.82 |
| LE_002865 | 2,035 | 240 | -3.08 | down | hypothetical protein GYMLUDRAFT_52125 | *G. luxurians* FD-317 M1 | 6E-85 | 49.68 |
| LE_002117 | 595 | 70 | -3.08 | down | polysaccharide lyase family 1 protein | *Trichoderma atroviride* IMI 206040 | 4E-166 | 69.58 |
| LE_000718 | 2,185 | 258 | -3.08 | down | hypothetical protein GYMLUDRAFT_43248 | *G. luxurians* FD-317 M1 | 6E-59 | 65.65 |
| LE_003716 | 132 | 15 | -3.09 | down | hypothetical protein GYMLUDRAFT_253489 | *G. luxurians* FD-317 M1 | 4E-126 | 56.18 |
| LE_000174 | 8,137 | 957 | -3.09 | down | hypothetical protein GYMLUDRAFT_52525 | *G. luxurians* FD-317 M1 | 6E-132 | 65.59 |
| LE_000403 | 2,158 | 251 | -3.10 | down | nucleoside diphosphate kinase | *Auricularia subglabra* TFB-10046 SS5 | 8E-83 | 89.21 |
| LE_002381 | 124 | 14 | -3.10 | down | glycoside hydrolase family 16 protein | *G. luxurians* FD-317 M1 | 2E-139 | 88.70 |
| LE_000265 | 15,977 | 1,848 | -3.11 | down | hypothetical protein GYMLUDRAFT_195709 | *G. luxurians* FD-317 M1 | 3E-131 | 42.56 |
| LE_001011 | 743 | 86 | -3.11 | down | choline dehydrogenase 6 | *H. irregulare* TC 32-1 | 0 | 54.44 |
| LE_001408 | 328 | 37 | -3.12 | down | P-loop containing nucleoside triphosphate hydrolase protein | *C. torrendii* FP15055 ss-10 | 0 | 62.16 |
| LE_008489 | 534 | 61 | -3.12 | down | enolase C-terminal domain-like protein | *C. torrendii* FP15055 ss-10 | 0 | 86.91 |
| LE_008919 | 338 | 38 | -3.12 | down | hypothetical protein GYMLUDRAFT_195081 | *G. luxurians* FD-317 M1 | 0 | 85.13 |
| LE_000713 | 3,915 | 449 | -3.12 | down | hypothetical protein GYMLUDRAFT_49011 | *G. luxurians* FD-317 M1 | 0 | 78.90 |
| LE_009326 | 141 | 16 | -3.13 | down | hypothetical protein GYMLUDRAFT_72317 | *G. luxurians* FD-317 M1 | 1E-146 | 61.73 |
| LE_008929 | 554 | 63 | -3.13 | down | glycoside hydrolase family 17 protein | *G. luxurians* FD-317 M1 | 0 | 68.96 |
| LE_005141 | 207 | 23 | -3.13 | down | RTA1-like protein | *S. hirsutum* FP-91666 SS1 | 1E-98 | 55.17 |
| LE_002291 | 140 | 15 | -3.14 | down | hypothetical protein GYMLUDRAFT_68583 | *G. luxurians* FD-317 M1 | 6E-74 | 53.74 |
| LE_003836 | 241 | 27 | -3.14 | down | short-chain dehydrogenase/reductase | *L. edodes* | 4E-145 | 66.99 |
| LE_010282 | 182 | 20 | -3.14 | down | hypothetical protein CONPUDRAFT_169644 | *C. puteana* RWD-64-598 SS2 | 1E-16 | 29.08 |
| LE_001010 | 883 | 100 | -3.14 | down | hypothetical protein GYMLUDRAFT_371469 | *G. luxurians* FD-317 M1 | 0 | 77.56 |
| LE_003590 | 198 | 22 | -3.14 | down | carbohydrate esterase family 3 protein | *C. torrendii* FP15055 ss-10 | 1E-85 | 69.90 |
| LE_008349 | 155 | 17 | -3.15 | down | polysaccharide lyase family 1 protein | *T. atroviride* IMI 206040 | 2E-146 | 71.64 |
| LE_004499 | 225 | 25 | -3.16 | down | NAD-P-binding protein | *S. hirsutum* FP-91666 SS1 | 7E-131 | 68.85 |
| LE_003425 | 3,428 | 383 | -3.16 | down | hypothetical protein GYMLUDRAFT_33285 | *G. luxurians* FD-317 M1 | 0 | 57.09 |
| LE_002025 | 274 | 30 | -3.16 | down | hypothetical protein GYMLUDRAFT_40539 | *G. luxurians* FD-317 M1 | 1E-140 | 63.84 |
| LE_000924 | 1,329 | 147 | -3.17 | down | phospholipid-translocating P-type ATPase | *C. torrendii* FP15055 ss-10 | 0 | 59.55 |
| LE_009169 | 255 | 28 | -3.18 | down | hypothetical protein GYMLUDRAFT_159555 | *G. luxurians* FD-317 M1 | 8E-93 | 77.51 |
| LE_009842 | 177 | 19 | -3.18 | down | hypothetical protein GYMLUDRAFT_66562 | *G. luxurians* FD-317 M1 | 2E-145 | 81.23 |
| LE_003399 | 427 | 47 | -3.18 | down |  |  |  |  |
| LE_008598 | 624 | 68 | -3.19 | down | D-lactaldehyde dehydrogenase | *C. puteana* RWD-64-598 SS2 | 1E-132 | 57.87 |
| LE_000274 | 26,713 | 2,925 | -3.19 | down | methionine adenosyltransferase | *Paxillus involutus* ATCC 200175 | 0 | 84.18 |
| LE_000649 | 653 | 71 | -3.19 | down | hypothetical protein GYMLUDRAFT_42206 | *G. luxurians* FD-317 M1 | 4E-12 | 68.42 |
| LE_009371 | 337 | 36 | -3.20 | down |  |  |  |  |
| LE_009212 | 505 | 54 | -3.21 | down | Carboxypeptidase S1 | *Termitomyces sp. J132* | 0 | 70.72 |
| LE_009173 | 2,189 | 237 | -3.21 | down |  |  |  |  |
| LE_008426 | 617 | 66 | -3.22 | down |  |  |  |  |
| LE_002069 | 172 | 18 | -3.22 | down | hypothetical protein GYMLUDRAFT_48009 | *G. luxurians* FD-317 M1 | 2E-125 | 74.25 |
| LE_006592 | 125 | 13 | -3.23 | down | hypothetical protein GYMLUDRAFT_43507 | *G. luxurians* FD-317 M1 | 2E-73 | 72.19 |
| LE_008691 | 325 | 34 | -3.24 | down |  |  |  |  |
| LE_002964 | 1,948 | 206 | -3.24 | down | 1-Cys peroxiredoxin isozyme | *Taiwanofungus camphoratus* | 9E-84 | 77.91 |
| LE_009487 | 532 | 56 | -3.24 | down | hypothetical protein GYMLUDRAFT_240230 | *G. luxurians* FD-317 M1 | 0 | 70.68 |
| LE_001926 | 314 | 33 | -3.25 | down | sulfate permease | *G. trabeum* ATCC 11539 | 0 | 73.59 |
| LE_009019 | 302 | 31 | -3.25 | down | SPOSA6832_02190 | *Sporidiobolus salmonicolor* | 4E-12 | 43.30 |
| LE_000533 | 2,582 | 269 | -3.26 | down | hypothetical protein GYMLUDRAFT_245443 | *G. luxurians* FD-317 M1 | 0 | 50.19 |
| LE_002107 | 390 | 40 | -3.26 | down | hypothetical protein GYMLUDRAFT_51375 | *G. luxurians* FD-317 M1 | 0 | 66.81 |
| LE_000553 | 978 | 100 | -3.28 | down | aldo/keto reductase | *G. trabeum* ATCC 11539 | 4E-135 | 71.76 |
| LE_008147 | 216 | 22 | -3.28 | down |  |  |  |  |
| LE_000298 | 17,129 | 1,763 | -3.28 | down | carbohydrate-binding module family 12 protein | *G. luxurians* FD-317 M1 | 6E-142 | 77.82 |
| LE_005321 | 115 | 11 | -3.29 | down | pantothenate transporter *liz1* | *C. torrendii* FP15055 ss-10 | 0 | 69.40 |
| LE_008943 | 113 | 11 | -3.31 | down | hypothetical protein GYMLUDRAFT_94488 | *G. luxurians* FD-317 M1 | 6E-30 | 41.72 |
| LE_000984 | 1,365 | 137 | -3.31 | down | hypothetical protein GYMLUDRAFT_86838 | *G. luxurians* FD-317 M1 | 0 | 76.69 |
| LE_002063 | 204 | 20 | -3.31 | down | hypothetical protein GYMLUDRAFT_34175 | *G. luxurians* FD-317 M1 | 3E-180 | 65.02 |
| LE_000772 | 4,637 | 468 | -3.31 | down | leptomycin B resistance protein *pmd1* | *Termitomyces sp.* J132 | 0 | 73.69 |
| LE_003406 | 1,224 | 123 | -3.31 | down | hypothetical protein GYMLUDRAFT_240138 | *G. luxurians* FD-317 M1 | 1E-100 | 73.87 |
| LE_003601 | 101 | 10 | -3.33 | down | hypothetical protein GYMLUDRAFT_35936 | *G. luxurians* FD-317 M1 | 0 | 83.75 |
| LE_000308 | 781 | 77 | -3.33 | down | hypothetical protein GYMLUDRAFT_36656 | *G. luxurians* FD-317 M1 | 9E-174 | 73.60 |
| LE_000870 | 1,754 | 174 | -3.33 | down | hypothetical protein GYMLUDRAFT_55274 | *G. luxurians* FD-317 M1 | 1E-130 | 72.13 |
| LE_008782 | 213 | 21 | -3.33 | down | MFS general substrate transporter | *C. torrendii* FP15055 ss-10 | 0 | 60.72 |
| LE_000606 | 7,669 | 757 | -3.34 | down | laccase 10 | *L. edodes* | 0 | 100.00 |
| LE_000985 | 2,396 | 237 | -3.34 | down | hypothetical protein GYMLUDRAFT_74134 | *G. luxurians* FD-317 M1 | 0 | 72.98 |
| LE_000120 | 1,154 | 114 | -3.34 | down | hypothetical protein GYMLUDRAFT_251419 | *G. luxurians* FD-317 M1 | 3E-93 | 57.96 |
| LE_001341 | 641 | 63 | -3.35 | down | hypothetical protein GYMLUDRAFT_240230 | *G. luxurians* FD-317 M1 | 0 | 69.05 |
| LE_003904 | 926 | 91 | -3.35 | down | hypothetical protein GYMLUDRAFT_38405 | *G. luxurians* FD-317 M1 | 3E-116 | 72.46 |
| LE_000272 | 18,010 | 1,748 | -3.36 | down | glycoside hydrolase family 16 protein | *G. luxurians* FD-317 M1 | 3E-152 | 70.00 |
| LE_002328 | 135 | 13 | -3.36 | down | hypothetical protein GYMLUDRAFT_171932 | *G. luxurians* FD-317 M1 | 0 | 68.91 |
| LE_001014 | 133 | 12 | -3.37 | down | hypothetical protein GYMLUDRAFT_237265 | *G. luxurians* FD-317 M1 | 4E-83 | 46.18 |
| LE_000406 | 4,723 | 452 | -3.38 | down | acid protease | *P. strigosozonata* HHB-11173 SS5 | 2E-133 | 57.43 |
| LE_000814 | 624 | 59 | -3.39 | down | NAD P-binding protein | *G. trabeum* ATCC 11539 | 1E-145 | 62.14 |
| LE_001121 | 1,839 | 174 | -3.40 | down | acetyl-CoA synthetase-like protein | *C. torrendii* FP15055 ss-10 | 0 | 65.59 |
| LE_008565 | 137 | 12 | -3.41 | down | alpha/beta-hydrolase | *Trametes versicolor* FP-101664 SS1 | 7E-79 | 45.91 |
| LE_008389 | 1,079 | 101 | -3.41 | down | hypothetical protein GYMLUDRAFT_36950 | *G. luxurians* FD-317 M1 | 2E-18 | 37.67 |
| LE_008400 | 1,496 | 140 | -3.41 | down |  |  |  |  |
| LE_003475 | 1,431 | 134 | -3.41 | down | sulfite reductase [NADPH] flavoprotein component | *Termitomyces sp.* J132 | 0 | 63.50 |
| LE_001685 | 356 | 33 | -3.41 | down | glycoside hydrolase family 3 protein | *G. luxurians* FD-317 M1 | 0 | 81.45 |
| LE_008154 | 1,063 | 100 | -3.41 | down | alcohol dehydrogenase superfamily protein | *C. cinerea* okayama7#130 | 3E-125 | 61.18 |
| LE_002059 | 1,043 | 97 | -3.42 | down | glycoside hydrolase family 3 protein | *G. luxurians* FD-317 M1 | 0 | 79.56 |
| LE_008118 | 3,485 | 326 | -3.42 | down | queuine tRNA-ribosyltransferase | *C. cinerea* okayama7#130 | 0 | 78.88 |
| LE_000556 | 242 | 22 | -3.43 | down | carbohydrate-binding module family 35 protein | *G. luxurians* FD-317 M1 | 0 | 81.94 |
| LE_008334 | 3,426 | 317 | -3.43 | down | hypothetical protein GYMLUDRAFT_991661 | *G. luxurians* FD-317 M1 | 0 | 81.82 |
| LE_009184 | 285 | 26 | -3.43 | down | hypothetical protein GYMLUDRAFT_68332 | *G. luxurians* FD-317 M1 | 3E-105 | 75.98 |
| LE_000309 | 565 | 52 | -3.43 | down | Clavaminate synthase-like protein | *C. torrendii* FP15055 ss-10 | 1E-173 | 63.61 |
| LE_008852 | 125 | 11 | -3.43 | down | ribosomal protein S3 (mitochondrion) | *L. edodes* | 7E-129 | 100.00 |
| LE_009522 | 139 | 12 | -3.44 | down | aldolase | *G. trabeum* ATCC 11539 | 4E-144 | 74.31 |
| LE_008520 | 1,143 | 104 | -3.44 | down | glycoside hydrolase family 61 protein | *P. crispa* FD-325 SS-3 | 2E-114 | 77.35 |
| LE_000648 | 13,659 | 1,251 | -3.45 | down | glycoside hydrolase family 7 protein | *G. luxurians* FD-317 M1 | 0 | 86.99 |
| LE_004788 | 384 | 35 | -3.46 | down | glycoside hydrolase family 3 protein | *G. luxurians* FD-317 M1 | 0 | 86.06 |
| LE_001725 | 142 | 13 | -3.46 | down | lectin | *P. ostreatus* | 2E-25 | 31.41 |
| LE_000173 | 180 | 16 | -3.46 | down | hypothetical protein GYMLUDRAFT_47361 | *G. luxurians* FD-317 M1 | 3E-66 | 42.25 |
| LE_000917 | 1,481 | 133 | -3.47 | down | hypothetical protein GYMLUDRAFT_36967 | *G. luxurians* FD-317 M1 | 0 | 45.28 |
| LE_008180 | 604 | 54 | -3.48 | down | dienelactone hydrolase | *Gelatoporia subvermispora* B | 9E-168 | 82.91 |
| LE_009060 | 486 | 43 | -3.48 | down | glycosyltransferase family 1 protein | *G. luxurians* FD-317 M1 | 2E-71 | 64.50 |
| LE_000774 | 723 | 65 | -3.48 | down | L-aminoadipate-semialdehyde dehydrogenase | *C. torrendii* FP15055 ss-10 | 0 | 65.76 |
| LE_000547 | 2,357 | 209 | -3.49 | down | hypothetical protein GYMLUDRAFT_565955 | *G. luxurians* FD-317 M1 | 2E-174 | 68.00 |
| LE_001829 | 571 | 50 | -3.50 | down | heterotrimeric GTP-binding alpha subunit | *C. torrendii* FP15055 ss-10 | 2E-150 | 55.19 |
| LE_001055 | 377 | 33 | -3.50 | down | hypothetical protein GYMLUDRAFT_238872 | *G. luxurians* FD-317 M1 | 0 | 77.46 |
| LE_008130 | 916 | 80 | -3.51 | down | PEBP protein | *S. hirsutum* FP-91666 SS1 | 3E-66 | 57.58 |
| LE_000214 | 5,848 | 512 | -3.51 | down | Mn superoxide dismutase 1 | *Volvariella volvacea* | 2E-107 | 83.51 |
| LE_008694 | 1,490 | 130 | -3.52 | down | hypothetical protein GYMLUDRAFT_177797 | *G. luxurians* FD-317 M1 | 1E-26 | 76.67 |
| LE_001298 | 1,508 | 130 | -3.53 | down | tartrate transporter | *Termitomyces sp.* J132 | 6E-108 | 64.44 |
| LE_003422 | 124 | 10 | -3.53 | down | hypothetical protein GYMLUDRAFT_88244 | *G. luxurians* FD-317 M1 | 9E-170 | 72.95 |
| LE_003367 | 412 | 35 | -3.54 | down | hypothetical protein GYMLUDRAFT_188807 | *G. luxurians* FD-317 M1 | 6E-90 | 70.05 |
| LE_000221 | 1,046 | 89 | -3.55 | down | short-chain dehydrogenase/reductase SDR | *C. cinerea* okayama7#130 | 2E-96 | 64.29 |
| LE_009241 | 278 | 23 | -3.55 | down |  |  |  |  |
| LE_000220 | 27,429 | 2,337 | -3.55 | down | disulfide isomerase | *L. edodes* | 0 | 98.79 |
| LE_008575 | 234 | 19 | -3.55 | down | hypothetical protein GYMLUDRAFT_67341 | *G. luxurians* FD-317 M1 | 2E-162 | 75.08 |
| LE_000208 | 7,530 | 644 | -3.55 | down |  |  |  |  |
| LE_001590 | 992 | 83 | -3.56 | down | hypothetical protein GYMLUDRAFT_170207 | *G. luxurians* FD-317 M1 | 7E-132 | 67.44 |
| LE_008254 | 826 | 70 | -3.56 | down | amine oxidase, partial | *F. hepatica* ATCC 64428 | 2E-172 | 61.27 |
| LE_000721 | 3,938 | 330 | -3.57 | down | hypothetical protein GYMLUDRAFT_47953 | *G. luxurians* FD-317 M1 | 0 | 83.19 |
| LE_000867 | 10,255 | 861 | -3.57 | down | H+/Ca2+ exchanger Vxc1-like protein | *H. irregulare* TC 32-1 | 1E-137 | 44.83 |
| LE_000313 | 4,358 | 367 | -3.57 | down | tripeptidyl peptidase A | *S. paradoxa* | 0 | 66.17 |
| LE_001569 | 886 | 74 | -3.57 | down | hypothetical protein GYMLUDRAFT_43217 | *G. luxurians* FD-317 M1 | 0 | 83.17 |
| LE_002047 | 713 | 59 | -3.59 | down | hypothetical protein GYMLUDRAFT_32719 | *G. luxurians* FD-317 M1 | 0 | 72.12 |
| LE_000927 | 1,783 | 148 | -3.59 | down | hypothetical protein GALMADRAFT_229731 | *Galerina marginata* CBS 339.88 | 6E-125 | 48.85 |
| LE_000789 | 8,785 | 729 | -3.59 | down | glycoside hydrolase family 27 protein | *G. luxurians* FD-317 M1 | 0 | 78.11 |
| LE_008156 | 4,654 | 381 | -3.61 | down | hypothetical protein HYPSUDRAFT_187841 | *Hypholoma sublateritium* FD-334 SS-4 | 2E-80 | 51.00 |
| LE_000251 | 5,066 | 415 | -3.61 | down | glycoside hydrolase family 16 protein | *G. luxurians* FD-317 M1 | 0 | 83.55 |
| LE_002308 | 1,225 | 99 | -3.62 | down | Zinc/iron permease | *C. torrendii* FP15055 ss-10 | 4E-116 | 52.41 |
| LE_000578 | 3,994 | 322 | -3.63 | down | hypothetical protein GYMLUDRAFT_317892 | *G. luxurians* FD-317 M1 | 0 | 75.89 |
| LE_001108 | 547 | 44 | -3.63 | down | hypothetical protein GYMLUDRAFT_77027 | *G. luxurians* FD-317 M1 | 3E-97 | 76.95 |
| LE_003833 | 8,545 | 692 | -3.63 | down | U3 small nucleolar ribonucleoprotein *mpp10* | *Termitomyces sp.* J132 | 2E-146 | 58.33 |
| LE_006241 | 258 | 20 | -3.64 | down | ICS | *L. edodes* | 0 | 99.66 |
| LE_000511 | 465 | 36 | -3.65 | down | S-adenosyl-L-methionine-dependent methyltransferase | *P. strigosozonata* HHB-11173 SS5 | 3E-104 | 57.10 |
| LE_001514 | 308 | 24 | -3.65 | down | CBD9-like protein | *P. strigosozonata* HHB-11173 SS5 | 2E-106 | 44.19 |
| LE_009005 | 103 | 8 | -3.65 | down |  |  |  |  |
| LE_000650 | 1,459 | 115 | -3.66 | down | UPF0187-domain-containing protein | *C. torrendii* FP15055 ss-10 | 1E-172 | 53.87 |
| LE_004779 | 1,659 | 130 | -3.67 | down | malate synthase | *L. bicolor* S238N-H82 | 0 | 80.19 |
| LE_000948 | 276 | 21 | -3.67 | down | glycoside hydrolase | *Dacryopinax sp.* DJM-731 SS1 | 4E-142 | 65.13 |
| LE_000780 | 1,242 | 97 | -3.68 | down | glycoside hydrolase family 92 protein | *G. luxurians* FD-317 M1 | 0 | 78.52 |
| LE_002945 | 353 | 27 | -3.68 | down | hypothetical protein MPER_12757 | *M. perniciosa* FA553 | 0 | 56.82 |
| LE_002296 | 518 | 40 | -3.69 | down | carbohydrate-binding module family 20 protein | *G. luxurians* FD-317 M1 | 0 | 81.57 |
| LE_003257 | 1,852 | 142 | -3.70 | down | putative GATA type transcriptional activator | *L. edodes* | 2E-139 | 99.68 |
| LE_008427 | 1,014 | 78 | -3.70 | down | hypothetical protein PILCRDRAFT_820439 | *P. croceum* F 1598 | 0 | 74.23 |
| LE_001033 | 854 | 65 | -3.70 | down | hypothetical protein GYMLUDRAFT_47563 | *G. luxurians* FD-317 M1 | 2E-156 | 65.61 |
| LE_002876 | 1,282 | 97 | -3.71 | down | hypothetical protein HYPSUDRAFT_32000 | *H. sublateritium* FD-334 SS-4 | 0 | 65.64 |
| LE_001451 | 411 | 31 | -3.71 | down | Sulfate adenylyltransferase | *Termitomyces sp.* J132 | 0 | 81.63 |
| LE_008224 | 246 | 19 | -3.71 | down |  |  |  |  |
| LE_000140 | 250,118 | 18,970 | -3.72 | down | hypothetical protein GYMLUDRAFT_44778 | *G. luxurians* FD-317 M1 | 0 | 91.23 |
| LE_004914 | 229 | 17 | -3.73 | down | cytochrome P450 | *Dichomitus squalens* LYAD-421 SS1 | 0 | 54.64 |
| LE_008128 | 359 | 27 | -3.73 | down | hypothetical protein GYMLUDRAFT_247307 | *G. luxurians* FD-317 M1 | 2E-150 | 68.62 |
| LE_000561 | 4,480 | 335 | -3.74 | down | NAP-domain-containing protein | *C. puteana* RWD-64-598 SS2 | 6E-164 | 71.82 |
| LE_010150 | 333 | 24 | -3.74 | down | hypothetical protein GYMLUDRAFT_246382 | *G. luxurians* FD-317 M1 | 0 | 77.67 |
| LE_002275 | 4,754 | 356 | -3.74 | down | hypothetical protein GYMLUDRAFT_244896 | *G. luxurians* FD-317 M1 | 0 | 59.85 |
| LE_002934 | 2,909 | 217 | -3.75 | down | neutral/alkaline nonlysosomal ceramidase | *T. versicolor* FP-101664 SS1 | 0 | 76.05 |
| LE_008166 | 1,785 | 130 | -3.77 | down | hypothetical protein GYMLUDRAFT_83412 | *G. luxurians* FD-317 M1 | 5E-164 | 43.13 |
| LE_002704 | 109 | 8 | -3.77 | down | O-methyltransferase family 3 protein | *C. cinerea* okayama7#130 | 3E-104 | 66.22 |
| LE_004665 | 528 | 38 | -3.77 | down | FMN-linked oxidoreductase | *C. torrendii* FP15055 ss-10 | 1E-147 | 57.63 |
| LE_002706 | 46,158 | 3,384 | -3.77 | down | hypothetical protein GLOTRDRAFT_52896 | *G. trabeum* ATCC 11539 | 0 | 86.83 |
| LE_009108 | 6,732 | 486 | -3.79 | down | glycoside hydrolase family 17 protein | *G. luxurians* FD-317 M1 | 0 | 73.48 |
| LE_004030 | 835 | 59 | -3.80 | down | hypothetical protein GYMLUDRAFT_68402 | *G. luxurians* FD-317 M1 | 2E-177 | 72.62 |
| LE_001371 | 126 | 9 | -3.80 | down | hypothetical protein GYMLUDRAFT_57220 | *G. luxurians* FD-317 M1 | 2E-163 | 84.59 |
| LE_008511 | 280 | 19 | -3.82 | down | HLH transcription factor | *L. edodes* | 3E-45 | 41.83 |
| LE_004005 | 938 | 66 | -3.82 | down | aldo/keto reductase | *C. torrendii* FP15055 ss-10 | 6E-127 | 67.8 |
| LE_000323 | 4,547 | 322 | -3.82 | down | hypothetical protein GYMLUDRAFT_41222 | *G. luxurians* FD-317 M1 | 1E-12 | 44.44 |
| LE_002649 | 1,145 | 80 | -3.83 | down | hypothetical protein GYMLUDRAFT_37816 | *G. luxurians* FD-317 M1 | 1E-79 | 90.06 |
| LE_001900 | 253 | 17 | -3.83 | down | hypothetical protein GYMLUDRAFT_45273 | *G. luxurians* FD-317 M1 | 3E-170 | 74.53 |
| LE_002919 | 111 | 7 | -3.83 | down |  |  |  |  |
| LE_002194 | 753 | 52 | -3.84 | down | glycoside hydrolase family 3 protein | *G. luxurians* FD-317 M1 | 0 | 79.18 |
| LE_003101 | 3,059 | 214 | -3.84 | down | DUF866-domain-containing protein | *T. versicolor* FP-101664 SS1 | 2E-73 | 73.46 |
| LE_006229 | 158 | 11 | -3.84 | down | MFS general substrate transporter | *C. torrendii* FP15055 ss-10 | 0 | 72.88 |
| LE_008192 | 523 | 36 | -3.85 | down |  |  |  |  |
| LE_002147 | 115 | 7 | -3.86 | down | hypothetical protein GYMLUDRAFT_196905 | *G. luxurians* FD-317 M1 | 3E-108 | 51.52 |
| LE_008978 | 303 | 20 | -3.87 | down | hypothetical protein GYMLUDRAFT_34490 | *G. luxurians* FD-317 M1 | 1E-18 | 45.58 |
| LE_002269 | 250 | 16 | -3.88 | down | short-chain dehydrogenase/reductase | *L. edodes* | 8E-135 | 70.55 |
| LE_001723 | 297 | 20 | -3.88 | down | hypothetical protein GYMLUDRAFT_43999 | *G. luxurians* FD-317 M1 | 3E-41 | 35.42 |
| LE_000943 | 1,913 | 130 | -3.88 | down | hypothetical protein GYMLUDRAFT_32723 | *G. luxurians* FD-317 M1 | 0 | 84.28 |
| LE_008481 | 602 | 40 | -3.89 | down | NAD dependent epimerase/dehydratase | *Rasamsonia emersonii* CBS 393.64 | 5E-86 | 47.19 |
| LE_008695 | 175 | 11 | -3.89 | down | proteasome (prosome, macropain) activator subunit 3 (pa28 gamma) | *R. solani* AG-3 Rhs1AP | 2E-54 | 48.12 |
| LE_002092 | 284 | 19 | -3.89 | down | glycoside hydrolase family 45 protein | *G. luxurians* FD-317 M1 | 1E-109 | 84.21 |
| LE_005644 | 2,758 | 184 | -3.90 | down | general substrate transporter | *C. torrendii* FP15055 ss-10 | 0 | 68.94 |
| LE_009473 | 150 | 10 | -3.90 | down | hypothetical protein GYMLUDRAFT_43832 | *G. luxurians* FD-317 M1 | 1E-157 | 68.02 |
| LE_003523 | 286 | 19 | -3.90 | down | carbohydrate-binding module family 5 protein | *G. luxurians* FD-317 M1 | 0 | 78.91 |
| LE_008354 | 1,157 | 77 | -3.91 | down | DUF914-domain-containing protein | *S. paradoxa* | 0 | 66.67 |
| LE_000246 | 1,000 | 66 | -3.91 | down | hypothetical protein GYMLUDRAFT_230956 | *G. luxurians* FD-317 M1 | 7E-107 | 81.90 |
| LE_000443 | 10,626 | 690 | -3.94 | down | putative nonribosomal peptide synthetase | *Omphalotus olearius* | 0 | 74.35 |
| LE_002289 | 1,271 | 83 | -3.94 | down | hypothetical protein GYMLUDRAFT_766925 | *G. luxurians* FD-317 M1 | 0 | 72.38 |
| LE_002365 | 110 | 7 | -3.94 | down | hypothetical protein GYMLUDRAFT_54263 | *G. luxurians* FD-317 M1 | 1E-103 | 54.57 |
| LE_000324 | 1,418 | 93 | -3.94 | down | acid protease | *P. strigosozonata* HHB-11173 SS5 | 6E-98 | 53.85 |
| LE_002200 | 1,132 | 73 | -3.95 | down | glycoside hydrolase family 35 protein | *G. luxurians* FD-317 M1 | 0 | 79.19 |
| LE_000862 | 519 | 33 | -3.95 | down | UPF0676 protein | *Termitomyces sp.* J132 | 1E-173 | 66.57 |
| LE_003744 | 157 | 10 | -3.97 | down | hypothetical protein GYMLUDRAFT_41945 | *G. luxurians* FD-317 M1 | 0 | 73.79 |
| LE_004282 | 546 | 35 | -3.97 | down | glycoside hydrolase family 1 protein | *G. luxurians* FD-317 M1 | 0 | 84.38 |
| LE_009445 | 281 | 17 | -3.98 | down | polyamine transporter | *C. torrendii* FP15055 ss-10 | 0 | 65.71 |
| LE_001498 | 278 | 17 | -3.98 | down |  |  |  |  |
| LE_002246 | 302 | 19 | -3.98 | down | hypothetical protein GYMLUDRAFT_186015 | *G. luxurians* FD-317 M1 | 0 | 72.43 |
| LE_009569 | 1,361 | 85 | -3.99 | down | *AtrD*, ABC-transporter | *C. torrendii* FP15055 ss-10 | 0 | 71.26 |
| LE_002006 | 160 | 10 | -3.99 | down | Magnesium transport protein *CorA* | *Termitomyces sp.* J132 | 5E-131 | 43.45 |
| LE_008341 | 319 | 19 | -4.01 | down | serine carboxypeptidase S10 | *H. irregulare* TC 32-1 | 0 | 70.28 |
| LE_004386 | 460 | 28 | -4.01 | down | hypothetical protein GYMLUDRAFT_196081 | *G. luxurians* FD-317 M1 | 4E-57 | 60.62 |
| LE_008149 | 4,179 | 260 | -4.01 | down | glycoside hydrolase family 13 protein | *G. luxurians* FD-317 M1 | 0 | 79.62 |
| LE_000946 | 1,046 | 63 | -4.05 | down | alcohol oxidase | *S. hirsutum* FP-91666 SS1 | 0 | 61.78 |
| LE_008339 | 552 | 33 | -4.05 | down | hypothetical protein GYMLUDRAFT_262104 | *G. luxurians* FD-317 M1 | 5E-29 | 57.39 |
| LE_001045 | 788 | 47 | -4.05 | down | hypothetical protein GYMLUDRAFT_248375 | *G. luxurians* FD-317 M1 | 5E-130 | 60.65 |
| LE_008183 | 1,319 | 79 | -4.05 | down | hypothetical protein GYMLUDRAFT_73797 | *G. luxurians* FD-317 M1 | 3E-75 | 65.05 |
| LE_009139 | 504 | 30 | -4.07 | down | Glutathione S-transferase | *Termitomyces sp.* J132 | 2E-62 | 49.77 |
| LE_002864 | 130 | 7 | -4.08 | down | hypothetical protein GYMLUDRAFT_229492 | *G. luxurians* FD-317 M1 | 8E-126 | 77.53 |
| LE_000581 | 3,986 | 236 | -4.08 | down | exo-beta-1,3-glucanase | *L. edodes* | 0 | 99.60 |
| LE_008678 | 142 | 8 | -4.09 | down | hypothetical protein GYMLUDRAFT_195081 | *G. luxurians* FD-317 M1 | 0 | 75.12 |
| LE_005689 | 150 | 8 | -4.09 | down | hypothetical protein GYMLUDRAFT_250099 | *G. luxurians* FD-317 M1 | 2E-35 | 75.23 |
| LE_000501 | 5,848 | 344 | -4.09 | down |  |  |  |  |
| LE_002024 | 573 | 33 | -4.10 | down | cytochrome P450 | *T. versicolor* FP-101664 SS1 | 6E-133 | 41.72 |
| LE_001661 | 806 | 47 | -4.10 | down | hypothetical protein GYMLUDRAFT_56380 | *G. luxurians* FD-317 M1 | 0 | 78.77 |
| LE_004427 | 205 | 12 | -4.10 | down | general substrate transporter | *C. torrendii* FP15055 ss-10 | 0 | 77.06 |
| LE_000744 | 1,267 | 73 | -4.11 | down | hypothetical protein GYMLUDRAFT_41903 | *G. luxurians* FD-317 M1 | 3E-138 | 41.32 |
| LE_001037 | 1,562 | 88 | -4.14 | down | hypothetical protein GYMLUDRAFT_36722 | *G. luxurians* FD-317 M1 | 0 | 80.11 |
| LE_000960 | 1,015 | 57 | -4.14 | down | hypothetical protein GYMLUDRAFT_195081 | *G. luxurians* FD-317 M1 | 0 | 77.62 |
| LE_000157 | 21,877 | 1,236 | -4.15 | down | hypothetical protein GYMLUDRAFT_245235 | *G. luxurians* FD-317 M1 | 8E-45 | 74.19 |
| LE_003868 | 4,432 | 247 | -4.16 | down | hypothetical protein GYMLUDRAFT_246369 | *G. luxurians* FD-317 M1 | 2E-133 | 62.39 |
| LE_002928 | 145 | 8 | -4.16 | down | hypothetical protein GYMLUDRAFT_431980 | *G. luxurians* FD-317 M1 | 1E-173 | 72.94 |
| LE_000571 | 16,664 | 932 | -4.16 | down | hypothetical protein GYMLUDRAFT_54573 | *G. luxurians* FD-317 M1 | 2E-52 | 69.66 |
| LE_001541 | 221 | 12 | -4.18 | down | hypothetical protein GALMADRAFT_239970 | *G. marginata* CBS 339.88 | 1E-127 | 55.99 |
| LE_008641 | 223 | 12 | -4.18 | down | hypothetical protein GYMLUDRAFT_82433 | *G. luxurians* FD-317 M1 | 0 | 64.46 |
| LE_000358 | 5,228 | 286 | -4.19 | down | glycoside hydrolase family 5 protein | *G. luxurians* FD-317 M1 | 0 | 73.35 |
| LE_008451 | 323 | 17 | -4.19 | down | alpha/beta-hydrolase | *G. trabeum* ATCC 11539 | 0 | 80.23 |
| LE_000255 | 29,782 | 1,617 | -4.20 | down | hypothetical protein GYMLUDRAFT_167026 | *G. luxurians* FD-317 M1 | 0 | 66.20 |
| LE_000445 | 4,015 | 218 | -4.20 | down | hypothetical protein GYMLUDRAFT_265664 | *G. luxurians* FD-317 M1 | 1E-11 | 54.84 |
| LE_003056 | 171 | 9 | -4.20 | down | unnamed protein product, partial | *L. edodes* | 6E-15 | 73.83 |
| LE_003179 | 244 | 13 | -4.21 | down | Sterigmatocystin 8-O-methyltransferase | *Termitomyces sp.* J132 | 3E-105 | 48.80 |
| LE_003020 | 1,036 | 55 | -4.21 | down | hypothetical protein GYMLUDRAFT_54068 | *G. luxurians* FD-317 M1 | 1E-50 | 58.48 |
| LE_001682 | 470 | 25 | -4.23 | down | glycoside hydrolase family 28 protein | *G. luxurians* FD-317 M1 | 0 | 78.29 |
| LE_001482 | 588 | 31 | -4.23 | down | glycoside hydrolase family 30 protein | *G. luxurians* FD-317 M1 | 0 | 82.86 |
| LE_003430 | 176 | 9 | -4.24 | down | hypothetical protein GYMLUDRAFT_623918 | *G. luxurians* FD-317 M1 | 8E-55 | 61.18 |
| LE_000966 | 406 | 21 | -4.24 | down | hypothetical protein GYMLUDRAFT_239395 | *G. luxurians* FD-317 M1 | 3E-112 | 54.68 |
| LE_000860 | 1,218 | 64 | -4.25 | down | hypothetical protein GYMLUDRAFT_44079 | *G. luxurians* FD-317 M1 | 1E-77 | 81.08 |
| LE_000228 | 87,123 | 4,580 | -4.25 | down | hypothetical protein GYMLUDRAFT_150244 | *G. luxurians* FD-317 M1 | 6E-120 | 92.38 |
| LE_008517 | 348 | 18 | -4.26 | down | hypothetical protein HETIRDRAFT_419288 | *H. irregulare* TC 32-1 | 9E-84 | 38.98 |
| LE_003035 | 480 | 25 | -4.27 | down | hypothetical protein GYMLUDRAFT_44704 | *G. luxurians* FD-317 M1 | 3E-164 | 76.95 |
| LE_001493 | 195 | 10 | -4.27 | down | polysaccharide lyase family 4 protein | *G. luxurians* FD-317 M1 | 0 | 84.49 |
| LE_000286 | 1,032 | 53 | -4.28 | down | hypothetical protein GYMLUDRAFT_933232 | *G. luxurians* FD-317 M1 | 0 | 70.57 |
| LE_002260 | 190 | 9 | -4.28 | down |  |  |  |  |
| LE_000767 | 3,069 | 158 | -4.28 | down | hypothetical protein GYMLUDRAFT_46433 | *G. luxurians* FD-317 M1 | 0 | 71.95 |
| LE_000179 | 2,457 | 127 | -4.28 | down | hypothetical protein GYMLUDRAFT_45811 | *G. luxurians* FD-317 M1 | 4E-72 | 57.14 |
| LE_009210 | 233 | 11 | -4.29 | down | aryl-alcohol dehydrogenase | *L. edodes* | 0 | 100.00 |
| LE_002464 | 2,154 | 108 | -4.32 | down | glycoside hydrolase family 125 protein | *G. luxurians* FD-317 M1 | 0 | 85.71 |
| LE_000551 | 3,758 | 186 | -4.33 | down | hypothetical protein GYMLUDRAFT_256126 | *G. luxurians* FD-317 M1 | 5E-148 | 70.91 |
| LE_003256 | 428 | 21 | -4.34 | down | hypothetical protein GYMLUDRAFT_404877 | *G. luxurians* FD-317 M1 | 1E-15 | 31.39 |
| LE_001013 | 1,478 | 72 | -4.35 | down | cytochrome P450 1 | *L. edodes* | 0 | 59.72 |
| LE_000732 | 6,145 | 301 | -4.35 | down | glycoside hydrolase family 5 protein | *G. luxurians* FD-317 M1 | 0 | 80.34 |
| LE_003070 | 180 | 8 | -4.35 | down | hypothetical protein GYMLUDRAFT_41339 | *G. luxurians* FD-317 M1 | 0 | 87.77 |
| LE_004241 | 300 | 14 | -4.36 | down |  |  |  |  |
| LE_003900 | 1,116 | 54 | -4.37 | down | terpenoid synthase | *F. hepatica* ATCC 64428 | 6E-23 | 28.32 |
| LE_008255 | 865 | 42 | -4.37 | down | cytochrome P450 | *P. strigosozonata* HHB-11173 SS5 | 0 | 64.47 |
| LE_001000 | 1,074 | 51 | -4.37 | down | xyloglucan-specific endoglucanase | *L. edodes* | 2E-136 | 99.57 |
| LE_000991 | 502 | 24 | -4.37 | down |  |  |  |  |
| LE_000773 | 941 | 45 | -4.38 | down | hypothetical protein GYMLUDRAFT_55759 | *G. luxurians* FD-317 M1 | 7E-63 | 79.70 |
| LE_009092 | 2,941 | 141 | -4.38 | down | hypothetical protein GYMLUDRAFT_242026 | *G. luxurians* FD-317 M1 | 3E-43 | 60.84 |
| LE_000283 | 1,811 | 86 | -4.39 | down | GroES-like protein | *P. strigosozonata* HHB-11173 SS5 | 2E-128 | 59.76 |
| LE_002353 | 153 | 7 | -4.39 | down |  |  |  |  |
| LE_009164 | 1,390 | 65 | -4.41 | down | hypothetical protein GYMLUDRAFT_195951 | *G. luxurians* FD-317 M1 | 0 | 81.03 |
| LE_003938 | 703 | 32 | -4.42 | down | hypothetical protein GYMLUDRAFT_50065 | *G. luxurians* FD-317 M1 | 0 | 77.84 |
| LE_008355 | 1,964 | 91 | -4.43 | down | hypothetical protein GYMLUDRAFT_50748 | *G. luxurians* FD-317 M1 | 0 | 89.13 |
| LE_003151 | 1,661 | 77 | -4.43 | down | glycoside hydrolase family 1 protein | *G. luxurians* FD-317 M1 | 0 | 89.89 |
| LE_000701 | 1,316 | 61 | -4.43 | down | extracellular GDSL-like lipase/acylhydrolase | *Byssochlamys spectabilis* No. 5 | 9E-178 | 64.46 |
| LE_008747 | 214 | 9 | -4.44 | down | hypothetical protein GYMLUDRAFT_243477 | *G. luxurians* FD-317 M1 | 2E-61 | 51.66 |
| LE_003576 | 1,178 | 54 | -4.44 | down | hypothetical protein GYMLUDRAFT_33750 | *G. luxurians* FD-317 M1 | 0 | 83.24 |
| LE_003942 | 765 | 35 | -4.44 | down | hypothetical protein GYMLUDRAFT_889262 | *G. luxurians* FD-317 M1 | 2E-137 | 50.55 |
| LE_000229 | 4,100 | 189 | -4.44 | down | hypothetical protein GYMLUDRAFT_202684 | *G. luxurians* FD-317 M1 | 4E-33 | 68.42 |
| LE_002051 | 1,371 | 62 | -4.45 | down | hypothetical protein GYMLUDRAFT_39615 | *G. luxurians* FD-317 M1 | 3E-166 | 72.51 |
| LE_002084 | 177 | 8 | -4.45 | down | Serine protease inhibitor |  | 4E-49 | 60.99 |
| LE_009554 | 873 | 40 | -4.45 | down | alpha/beta-hydrolase | *S. paradoxa* | 0 | 72.02 |
| LE_008317 | 473 | 21 | -4.46 | down | lipase | *T. versicolor* FP-101664 SS1 | 2E-124 | 67.75 |
| LE_002794 | 440 | 19 | -4.46 | down | hypothetical protein GYMLUDRAFT_248559 | *G. luxurians* FD-317 M1 | 0 | 61.37 |
| LE_003332 | 378 | 17 | -4.48 | down | hypothetical protein GYMLUDRAFT_91918 | *G. luxurians* FD-317 M1 | 0 | 85.06 |
| LE_001652 | 109 | 4 | -4.50 | down | hypothetical protein GYMLUDRAFT_1004459 | *G. luxurians* FD-317 M1 | 5E-132 | 74.45 |
| LE_001253 | 1,861 | 81 | -4.51 | down | hypothetical protein RHOBADRAFT_49363 | *Rhodotorula graminis* WP1 | 6E-20 | 59.78 |
| LE_010138 | 172 | 7 | -4.52 | down | hypothetical protein GYMLUDRAFT_167102 | *G. luxurians* FD-317 M1 | 3E-137 | 75.39 |
| LE_008155 | 2,000 | 87 | -4.52 | down | hypothetical protein GYMLUDRAFT_98907 | *G. luxurians* FD-317 M1 | 0 | 68.46 |
| LE_002487 | 139 | 6 | -4.56 | down | hypothetical protein GYMLUDRAFT_509850 | *G. luxurians* FD-317 M1 | 7E-61 | 56.04 |
| LE_008484 | 377 | 15 | -4.57 | down | carbohydrate esterase family 15 protein | *G. luxurians* FD-317 M1 | 0 | 88.30 |
| LE_000409 | 8,755 | 365 | -4.58 | down | hypothetical protein GYMLUDRAFT_72277 | *G. luxurians* FD-317 M1 | 1E-48 | 75.66 |
| LE_000125 | 11,374 | 476 | -4.58 | down | hypothetical protein HYPSUDRAFT_48557 | *H. sublateritium* FD-334 SS-4 | 0 | 61.18 |
| LE_008351 | 897 | 37 | -4.59 | down | hypothetical protein GYMLUDRAFT_1020713 | *G. luxurians* FD-317 M1 | 1E-125 | 81.85 |
| LE_001295 | 1,353 | 55 | -4.62 | down | general substrate transporter | *C. torrendii* FP15055 ss-10 | 0 | 75.58 |
| LE_001178 | 1,132 | 46 | -4.62 | down | hypothetical protein HYPSUDRAFT_67708 | *H. sublateritium* FD-334 SS-4 | 3E-171 | 59.96 |
| LE_008311 | 1,002 | 40 | -4.62 | down | acyl-CoA synthetase | *A. bisporus var. bisporus* H97 | 0 | 60.03 |
| LE_009475 | 902 | 36 | -4.63 | down | hypothetical protein GALMADRAFT_147764 | *G. marginata CBS 339.88* | 3E-159 | 47.57 |
| LE_002030 | 684 | 27 | -4.64 | down | DUF1793-domain-containing protein | *P. strigosozonata* HHB-11173 SS5 | 0 | 71.09 |
| LE_008366 | 692 | 27 | -4.64 | down | NAD-P-binding protein | *S. hirsutum* FP-91666 SS1 | 8E-111 | 56.34 |
| LE_009089 | 217 | 8 | -4.65 | down | hypothetical protein GYMLUDRAFT_39757 | *G. luxurians* FD-317 M1 | 1E-148 | 58.91 |
| LE_008206 | 689 | 27 | -4.66 | down |  |  |  |  |
| LE_000493 | 7,503 | 296 | -4.66 | down | 5-aminolevulinate synthase, mitochondrial | *Termitomyces sp.* J132 | 0 | 75.45 |
| LE_000595 | 4,941 | 195 | -4.66 | down | hypothetical protein GYMLUDRAFT_81408 | *G. luxurians* FD-317 M1 | 2E-49 | 64.34 |
| LE_002951 | 1,931 | 76 | -4.67 | down | ZIP superfamily | *Pleurotus ostreatus* PC15 | 3E-131 | 61.88 |
| LE_000612 | 2,371 | 92 | -4.68 | down | glycoside hydrolase family 5 protein | *G. luxurians* FD-317 M1 | 0 | 81.36 |
| LE_000147 | 1,201 | 46 | -4.68 | down | MAP kinase | *L. edodes* | 0 | 72.83 |
| LE_004050 | 884 | 34 | -4.69 | down | hypothetical protein GYMLUDRAFT_228507 | *G. luxurians* FD-317 M1 | 0 | 68.11 |
| LE_002923 | 607 | 23 | -4.69 | down | hypothetical protein GYMLUDRAFT_263841 | *G. luxurians* FD-317 M1 | 1E-147 | 61.68 |
| LE_009223 | 196 | 7 | -4.72 | down | glycoside hydrolase family 25 protein | *G. luxurians* FD-317 M1 | 2E-114 | 84.13 |
| LE_003308 | 1,190 | 45 | -4.72 | down | hypothetical protein GYMLUDRAFT_37569 | *G. luxurians* FD-317 M1 | 0 | 89.38 |
| LE_000599 | 2,140 | 80 | -4.73 | down | hypothetical protein GYMLUDRAFT_201191 | *G. luxurians* FD-317 M1 | 0 | 76.34 |
| LE_001636 | 1,590 | 59 | -4.74 | down | O-methylsterigmatocystin oxidoreductase | *Termitomyces sp.* J132 | 0 | 61.02 |
| LE_008710 | 205 | 7 | -4.75 | down | choline transport protein | *P. strigosozonata* HHB-11173 SS5 | 2E-176 | 58.05 |
| LE_007893 | 779 | 28 | -4.76 | down | clavaminate synthase-like protein | *C. torrendii* FP15055 ss-10 | 0 | 71.27 |
| LE_000676 | 2,297 | 84 | -4.76 | down | glycoside hydrolase family 28 protein | *G. luxurians* FD-317 M1 | 0 | 82.71 |
| LE_000454 | 2,736 | 101 | -4.76 | down | hypothetical protein GYMLUDRAFT_69274 | *G. luxurians* FD-317 M1 | 0 | 65.11 |
| LE_001727 | 177 | 6 | -4.77 | down |  |  |  |  |
| LE_002176 | 132 | 4 | -4.77 | down | hypothetical protein GYMLUDRAFT_34902 | *G. luxurians* FD-317 M1 | 0 | 73.68 |
| LE_008551 | 337 | 12 | -4.77 | down | hypothetical protein PLICRDRAFT_45769 | *P. crispa* FD-325 SS-3 | 8E-43 | 37.45 |
| LE_000192 | 1,014 | 36 | -4.78 | down | putative MAP kinase | *L. edodes* | 0 | 81.20 |
| LE_004132 | 221 | 8 | -4.79 | down | methyltransferase-like protein 7B | *Termitomyces sp.* J132 | 1E-99 | 53.16 |
| LE_002673 | 862 | 30 | -4.82 | down |  |  |  |  |
| LE_009247 | 229 | 7 | -4.82 | down | hypothetical protein SERLADRAFT_464680 | *Serpula lacrymans var. lacrymans* S7.9 | 3E-47 | 37.97 |
| LE_000882 | 4,410 | 156 | -4.82 | down | hypothetical protein GYMLUDRAFT_69434 | *G. luxurians* FD-317 M1 | 0 | 88.40 |
| LE_001296 | 3,805 | 133 | -4.83 | down | alpha/beta-hydrolase | *P. strigosozonata* HHB-11173 SS5 | 0 | 74.60 |
| LE_001123 | 350 | 12 | -4.85 | down | carbonic anhydrase | *C. torrendii* FP15055 ss-10 | 4E-63 | 44.95 |
| LE_009137 | 5,429 | 188 | -4.85 | down | hypothetical protein GYMLUDRAFT_39575 | *G. luxurians FD-317 M1* | 1E-20 | 61.44 |
| LE_000911 | 724 | 24 | -4.86 | down | laccase | *L. edodes* | 0 | 99.81 |
| LE_008572 | 285 | 9 | -4.87 | down | Di-copper centre-containing protein | *F. mediterranea* MF3/22 | 2E-117 | 50.86 |
| LE_002525 | 219 | 7 | -4.87 | down | hypothetical protein HYDPIDRAFT_24675 | *Hydnomerulius pinastri* MD-312 | 0 | 48.73 |
| LE_000487 | 1,161 | 39 | -4.87 | down | hypothetical protein PLEOSDRAFT_1067048 | *P. ostreatus* PC15 | 0 | 57.89 |
| LE_004083 | 745 | 25 | -4.89 | down |  |  |  |  |
| LE_001111 | 810 | 27 | -4.90 | down | fatty acid conjugase | *C. cinerea* okayama7#130 | 0 | 60.20 |
| LE_000673 | 2,568 | 85 | -4.91 | down | cerato-platanin 6 | *Crinipellis campanella* | 1E-40 | 64.84 |
| LE_005698 | 110 | 3 | -4.92 | down |  |  |  |  |
| LE_001489 | 1,058 | 34 | -4.92 | down | hypothetical protein GYMLUDRAFT_66353 | *G. luxurians* FD-317 M1 | 3E-163 | 57.17 |
| LE_001028 | 1,143 | 38 | -4.92 | down | glycoside hydrolase family 16 protein | *G. luxurians* FD-317 M1 | 8E-178 | 81.94 |
| LE_004079 | 499 | 16 | -4.93 | down | hypothetical protein GYMLUDRAFT_920070 | *G. luxurians* FD-317 M1 | 3E-136 | 56.55 |
| LE_001778 | 467 | 15 | -4.93 | down |  |  |  |  |
| LE_009246 | 1,308 | 42 | -4.94 | down | hypothetical protein GYMLUDRAFT_40146 | *G. luxurians* FD-317 M1 | 7E-115 | 59.29 |
| LE_002057 | 263 | 8 | -4.94 | down |  |  |  |  |
| LE_009105 | 2,941 | 96 | -4.94 | down | glutathione S-transferase | *G. trabeum* ATCC 11539 | 1E-86 | 56.57 |
| LE_001733 | 237 | 7 | -4.94 | down | hypothetical protein GYMLUDRAFT_42811 | *G. luxurians* FD-317 M1 | 4E-73 | 50.00 |
| LE_004528 | 1,094 | 35 | -4.95 | down |  |  |  |  |
| LE_000502 | 205 | 6 | -4.97 | down | carbohydrate esterase family 12 protein | *G. luxurians* FD-317 M1 | 1E-122 | 75.00 |
| LE_001087 | 256 | 7 | -4.98 | down | D-amino-acid oxidase | *P. strigosozonata* HHB-11173 SS5 | 5E-102 | 43.88 |
| LE_000341 | 1,107 | 35 | -4.98 | down |  |  |  |  |
| LE_004791 | 721 | 22 | -4.98 | down | clavaminate synthase-like protein | *F. hepatica* ATCC 64428 | 1E-171 | 75.00 |
| LE_002197 | 140 | 4 | -4.99 | down | carbohydrate esterase family 16 protein | *G. luxurians* FD-317 M1 | 0 | 87.12 |
| LE_002364 | 502 | 15 | -5.01 | down | amidase signature enzyme | *F. mediterranea* MF3/22 | 0 | 78.05 |
| LE_008305 | 146 | 4 | -5.02 | down | hypothetical protein GYMLUDRAFT_36678 | *G. luxurians* FD-317 M1 | 2E-99 | 81.77 |
| LE_008538 | 527 | 16 | -5.02 | down | P-loop containing nucleoside triphosphate hydrolase protein | *P. strigosozonata* HHB-11173 SS5 | 1E-113 | 65.71 |
| LE_001017 | 6,124 | 186 | -5.03 | down |  |  |  |  |
| LE_001476 | 709 | 21 | -5.03 | down | putative family protein | *Eutypa lata* UCREL1 | 0 | 64.68 |
| LE_000903 | 1,592 | 48 | -5.04 | down | hypothetical protein GYMLUDRAFT_38479 | *G. luxurians* FD-317 M1 | 0 | 89.56 |
| LE_000134 | 8,277 | 250 | -5.05 | down | hypothetical protein GYMLUDRAFT_53698 | *G. luxurians* FD-317 M1 | 1E-137 | 57.93 |
| LE_006916 | 111 | 3 | -5.06 | down | hypothetical protein CYLTODRAFT_436349 | *C. torrendii* FP15055 ss-10 | 1E-75 | 44.36 |
| LE_000361 | 16,646 | 498 | -5.06 | down | xanthine/uracil permease | *G. trabeum* ATCC 11539 | 0 | 77.82 |
| LE_000825 | 112 | 3 | -5.06 | down | hypothetical protein GYMLUDRAFT_178157 | *G. luxurians* FD-317 M1 | 3E-126 | 45.74 |
| LE_004880 | 312 | 9 | -5.07 | down | hypothetical protein GYMLUDRAFT_222584 | *G. luxurians* FD-317 M1 | 0 | 79.79 |
| LE_003872 | 3,302 | 97 | -5.08 | down | expressed protein | *Schizophyllum commune* H4-8 | 4E-76 | 45.56 |
| LE_000151 | 14,450 | 427 | -5.08 | down | 60S ribosomal protein L23 saccharomyces | *R. solani* | 2E-79 | 94.03 |
| LE_000364 | 8,063 | 239 | -5.08 | down | carbohydrate-binding module family 1 protein | *G. luxurians* FD-317 M1 | 9E-141 | 81.20 |
| LE_003509 | 477 | 13 | -5.11 | down | iron-regulated transporter | *Coccidioides immitis* RS | 0 | 61.15 |
| LE_001752 | 186 | 5 | -5.11 | down | hypothetical protein GYMLUDRAFT_254693 | *G. luxurians* FD-317 M1 | 2E-160 | 71.59 |
| LE_008493 | 462 | 13 | -5.12 | down |  |  |  |  |
| LE_009392 | 141 | 3 | -5.13 | down | hypothetical protein GYMLUDRAFT_36678 | *G. luxurians* FD-317 M1 | 9E-88 | 81.77 |
| LE_009925 | 303 | 8 | -5.13 | down | alpha/beta-hydrolase | *G. trabeum* ATCC 11539 | 4E-115 | 58.90 |
| LE_002914 | 35,557 | 1,003 | -5.15 | down | hypothetical protein GYMLUDRAFT_227867 | *G. luxurians* FD-317 M1 | 3E-112 | 83.51 |
| LE_000680 | 8,539 | 238 | -5.16 | down | thioredoxin-like protein | *C. torrendii* FP15055 ss-10 | 3E-100 | 72.55 |
| LE_000614 | 2,466 | 69 | -5.16 | down | acid protease | *T. versicolor* FP-101664 SS1 | 9E-127 | 58.63 |
| LE_008889 | 178 | 4 | -5.17 | down | alpha/beta-hydrolase | *G. trabeum* ATCC 11539 | 4E-95 | 41.28 |
| LE_001078 | 921 | 25 | -5.17 | down | hypothetical protein GYMLUDRAFT_43375 | *G. luxurians* FD-317 M1 | 0 | 69.55 |
| LE_006001 | 138 | 3 | -5.17 | down | proline-specific peptidase | *G. trabeum* ATCC 11539 | 1E-124 | 59.20 |
| LE_001936 | 203 | 5 | -5.19 | down | Zinc-type alcohol dehydrogenase-like protein PB24D3.08c | *Termitomyces sp.* J132 | 3E-137 | 60.55 |
| LE_008202 | 108 | 2 | -5.20 | down | hypothetical protein GYMLUDRAFT_774192 | *G. luxurians* FD-317 M1 | 1E-105 | 68.89 |
| LE_003073 | 379 | 10 | -5.21 | down | hypothetical protein GYMLUDRAFT_68886 | *G. luxurians* FD-317 M1 | 0 | 76.00 |
| LE_008169 | 582 | 15 | -5.21 | down | hypothetical protein GYMLUDRAFT_49327 | *G. luxurians* FD-317 M1 | 7E-124 | 69.64 |
| LE_004697 | 297 | 7 | -5.22 | down |  |  |  |  |
| LE_001457 | 1,855 | 49 | -5.23 | down | NAD P-binding protein | *G. trabeum* ATCC 11539 | 1E-115 | 62.59 |
| LE_008476 | 193 | 5 | -5.23 | down | hypothetical protein GYMLUDRAFT_673861 | *G. luxurians* FD-317 M1 | 5E-55 | 53.52 |
| LE_000545 | 2,277 | 60 | -5.23 | down | hypothetical protein GYMLUDRAFT_250087 | *G. luxurians* FD-317 M1 | 6E-34 | 80.73 |
| LE_008604 | 258 | 6 | -5.24 | down | alpha beta-hydrolase | *C. puteana* RWD-64-598 SS2 | 4E-59 | 34.33 |
| LE_001998 | 387 | 10 | -5.25 | down |  |  |  |  |
| LE_008570 | 165 | 4 | -5.25 | down |  |  |  |  |
| LE_008310 | 8,330 | 215 | -5.27 | down | hypothetical protein GYMLUDRAFT_40212 | *G. luxurians* FD-317 M1 | 0 | 87.15 |
| LE_001209 | 576 | 14 | -5.27 | down | hypothetical protein GYMLUDRAFT_45751 | *G. luxurians* FD-317 M1 | 0 | 70.58 |
| LE_000485 | 1,097 | 28 | -5.28 | down | hypothetical protein GYMLUDRAFT_358211 | *G. luxurians* FD-317 M1 | 1E-48 | 41.81 |
| LE_010121 | 355 | 8 | -5.29 | down | D-isomer specific 2-hydroxyacid dehydrogenase | *F. mediterranea* MF3/22 | 5E-153 | 66.99 |
| LE_000668 | 2,348 | 59 | -5.29 | down | hypothetical protein GALMADRAFT_269502 | *G. marginata* CBS 339.88 | 1E-134 | 49.58 |
| LE_009546 | 1,079 | 27 | -5.31 | down | prolyl oligopeptidase | *Rhodosporidium toruloides* NP11 | 0 | 44.15 |
| LE_008713 | 203 | 4 | -5.31 | down |  |  |  |  |
| LE_000224 | 36,235 | 910 | -5.32 | down |  |  |  |  |
| LE_009245 | 1,509 | 37 | -5.35 | down | glycosyltransferase family 1 protein | *G. luxurians* FD-317 M1 | 0 | 52.12 |
| LE_001898 | 422 | 10 | -5.37 | down | PLP-dependent transferase | *C. puteana* RWD-64-598 SS2 | 0 | 72.55 |
| LE_001842 | 182 | 4 | -5.37 | down | hypothetical protein M378DRAFT_9838 | *A. muscaria* Koide BX008 | 5E-98 | 49.22 |
| LE_002067 | 183 | 4 | -5.38 | down | hypothetical protein GYMLUDRAFT_39649 | *G. luxurians* FD-317 M1 | 6E-94 | 55.84 |
| LE_000526 | 4,465 | 106 | -5.39 | down | hypothetical protein GYMLUDRAFT_46270 | *G. luxurians* FD-317 M1 | 0 | 81.33 |
| LE_002046 | 930 | 21 | -5.41 | down | hypothetical protein GYMLUDRAFT_72897 | *G. luxurians* FD-317 M1 | 3E-66 | 48.68 |
| LE_002402 | 133 | 3 | -5.42 | down | hypothetical protein PLICRDRAFT_37904 | *P. crispa* FD-325 SS-3 | 8E-100 | 48.24 |
| LE_000233 | 191,685 | 4,349 | -5.46 | down | hypothetical protein GYMLUDRAFT_32667 | *G. luxurians* FD-317 M1 | 1E-66 | 78.03 |
| LE_008286 | 1,377 | 31 | -5.47 | down | hypothetical protein GYMLUDRAFT_145162 | *G. luxurians* FD-317 M1 | 2E-170 | 68.38 |
| LE_008338 | 389 | 8 | -5.49 | down | hypothetical protein GYMLUDRAFT_44883 | *G. luxurians* FD-317 M1 | 6E-151 | 79.02 |
| LE_001310 | 166 | 3 | -5.49 | down |  |  |  |  |
| LE_000769 | 1,059 | 23 | -5.52 | down | aldos-2-ulose dehydratase | *P. chrysosporium* | 0 | 48.61 |
| LE_000189 | 27,799 | 599 | -5.53 | down | hypothetical protein GYMLUDRAFT_239927 | *G. luxurians* FD-317 M1 | 5E-108 | 72.13 |
| LE_002160 | 497 | 10 | -5.53 | down | NAD(P)-binding protein | *C. puteana* RWD-64-598 SS2 | 4E-141 | 76.63 |
| LE_002320 | 203 | 4 | -5.53 | down | hypothetical protein GYMLUDRAFT_207164 | *G. luxurians* FD-317 M1 | 2E-29 | 79.76 |
| LE_003154 | 281 | 5 | -5.54 | down | MFS general substrate transporter | *F. hepatica* ATCC 64428 | 0 | 68.07 |
| LE_008129 | 1,951 | 42 | -5.54 | down | hypothetical protein GYMLUDRAFT_833346 | *G. luxurians* FD-317 M1 | 2E-35 | 43.62 |
| LE_001136 | 609 | 13 | -5.55 | down | hypothetical protein GYMLUDRAFT_255444 | *G. luxurians* FD-317 M1 | 1E-140 | 66.17 |
| LE_000584 | 2,361 | 50 | -5.56 | down | hypothetical protein GYMLUDRAFT_262104 | *G. luxurians* FD-317 M1 | 1E-25 | 55.77 |
| LE_000215 | 117 | 2 | -5.57 | down | alpha/beta hydrolase fold-3 | *Penicillium expansum* | 2E-65 | 38.32 |
| LE_001452 | 570 | 12 | -5.58 | down | glycoside hydrolase family 28 protein | *G. luxurians* FD-317 M1 | 2E-171 | 78.02 |
| LE_000430 | 1,894 | 39 | -5.58 | down | hypothetical protein GYMLUDRAFT_239787 | *G. luxurians* FD-317 M1 | 0 | 60.17 |
| LE_010351 | 479 | 9 | -5.59 | down | laccase *lcc5* | *L. edodes* | 0 | 100.00 |
| LE_000656 | 176 | 3 | -5.59 | down | hypothetical protein GYMLUDRAFT_42342 | *G. luxurians* FD-317 M1 | 1E-133 | 62.12 |
| LE_008269 | 1,175 | 24 | -5.60 | down | hypothetical protein GYMLUDRAFT_89650 | *G. luxurians* FD-317 M1 | 3E-134 | 79.38 |
| LE_000216 | 91,354 | 1,868 | -5.61 | down | hypothetical protein GYMLUDRAFT_169219 | *G. luxurians* FD-317 M1 | 1E-118 | 90.28 |
| LE_008319 | 1,013 | 20 | -5.64 | down | hypothetical protein GYMLUDRAFT_45751 | *G. luxurians* FD-317 M1 | 0 | 70.43 |
| LE_004234 | 102 | 2 | -5.67 | down | hypothetical protein GYMLUDRAFT_40662 | *G. luxurians* FD-317 M1 | 5E-11 | 88.89 |
| LE_000332 | 375 | 7 | -5.67 | down | hypothetical protein GYMLUDRAFT_42251 | *G. luxurians* FD-317 M1 | 0 | 62.84 |
| LE_000564 | 3,184 | 62 | -5.68 | down | heme peroxidase | *S. hirsutum* FP-91666 SS1 | 0 | 63.46 |
| LE_008788 | 243 | 4 | -5.69 | down | unnamed protein product, partial | *L. edodes* | 7E-47 | 94.44 |
| LE_001005 | 627 | 12 | -5.70 | down | hypothetical protein GYMLUDRAFT_73243 | *G. luxurians* FD-317 M1 | 3E-156 | 75.35 |
| LE_001293 | 311 | 5 | -5.71 | down | hypothetical protein GYMLUDRAFT_50517 | *G. luxurians* FD-317 M1 | 4E-41 | 30.11 |
| LE_009402 | 311 | 5 | -5.71 | down | carbohydrate esterase family 16 protein | *G. luxurians* FD-317 M1 | 1E-172 | 80.89 |
| LE_001038 | 1,293 | 24 | -5.72 | down | L30e-like protein | *G. trabeum* ATCC 11539 | 4E-140 | 80.63 |
| LE_009126 | 13,744 | 257 | -5.74 | down | MAP kinase | *L. edodes* | 0 | 99.73 |
| LE_003879 | 630 | 11 | -5.74 | down | hypothetical protein GYMLUDRAFT_34359 | *G. luxurians* FD-317 M1 | 0 | 86.55 |
| LE_000531 | 5,025 | 93 | -5.75 | down | hypothetical protein GYMLUDRAFT_259715 | *G. luxurians* FD-317 M1 | 0 | 61.24 |
| LE_001265 | 140 | 2 | -5.76 | down | hypothetical protein GYMLUDRAFT_232637 | *G. luxurians* FD-317 M1 | 4E-22 | 80.43 |
| LE_008374 | 498 | 9 | -5.78 | down | hypothetical protein GYMLUDRAFT_849204 | *G. luxurians* FD-317 M1 | 1E-105 | 66.44 |
| LE_003415 | 178 | 3 | -5.79 | down | glycoside hydrolase family 10 protein | *G. luxurians* FD-317 M1 | 0 | 82.67 |
| LE_000156 | 3,964 | 71 | -5.79 | down | non-catalytic module family EXPN protein | *H. irregulare* TC 32-1 | 2E-40 | 69.52 |
| LE_000514 | 620 | 11 | -5.84 | down | hypothetical protein GYMLUDRAFT_80703 | *G. luxurians* FD-317 M1 | 6E-14 | 35.23 |
| LE_001065 | 149 | 2 | -5.86 | down | glycoside hydrolase family 45 protein | *G. luxurians* FD-317 M1 | 2E-113 | 85.28 |
| LE_003911 | 4,610 | 76 | -5.92 | down | hypothetical protein GYMLUDRAFT_46314 | *G. luxurians* FD-317 M1 | 1E-17 | 55.84 |
| LE_009177 | 241 | 3 | -5.95 | down | hypothetical protein GYMLUDRAFT_213420 | *G. luxurians* FD-317 M1 | 0 | 73.45 |
| LE_009873 | 1,754 | 27 | -5.99 | down | hypothetical protein GYMLUDRAFT_49488 | *G. luxurians* FD-317 M1 | 2E-62 | 60.20 |
| LE_002892 | 5,447 | 85 | -5.99 | down | hypothetical protein GYMLUDRAFT_242470 | *G. luxurians* FD-317 M1 | 2E-38 | 67.67 |
| LE_008193 | 975 | 15 | -6.00 | down | glycoside hydrolase family 51 protein | *G. luxurians* FD-317 M1 | 0 | 73.11 |
| LE_008745 | 322 | 4 | -6.05 | down | carbohydrate-binding module family 20 protein | *G. luxurians* FD-317 M1 | 0 | 82.62 |
| LE_000694 | 1,017 | 15 | -6.05 | down | hypothetical protein GYMLUDRAFT_48227 | *G. luxurians* FD-317 M1 | 2E-86 | 56.73 |
| LE_000306 | 439 | 6 | -6.05 | down | hypothetical protein GYMLUDRAFT_34764 | *G. luxurians* FD-317 M1 | 0 | 84.17 |
| LE_010057 | 116 | 1 | -6.06 | down |  |  |  |  |
| LE_001139 | 103 | 1 | -6.08 | down |  |  |  |  |
| LE_009516 | 324 | 4 | -6.12 | down | hypothetical protein MPER_10011 | *M. perniciosa* FA553 | 1E-50 | 38.18 |
| LE_003354 | 216 | 3 | -6.12 | down | carbohydrate esterase family 8 protein | *G. luxurians* FD-317 M1 | 2E-158 | 74.53 |
| LE_008487 | 365 | 5 | -6.13 | down | hypothetical protein GYMLUDRAFT_208774 | *G. luxurians* FD-317 M1 | 0 | 69.82 |
| LE_000787 | 1,530 | 22 | -6.13 | down | hypothetical protein GYMLUDRAFT_36042 | *G. luxurians* FD-317 M1 | 4E-50 | 51.93 |
| LE_004374 | 2,296 | 32 | -6.14 | down | acid protease | *S. hirsutum* FP-91666 SS1 | 2E-166 | 62.26 |
| LE_002447 | 998 | 13 | -6.20 | down | aldo/keto reductase | *F. hepatica* ATCC 64428 | 2E-177 | 74.40 |
| LE_008557 | 1,873 | 25 | -6.23 | down | hypothetical protein GYMLUDRAFT_1012811 | *G. luxurians* FD-317 M1 | 0 | 77.98 |
| LE_001472 | 510 | 6 | -6.23 | down |  |  |  |  |
| LE_000227 | 12,441 | 164 | -6.25 | down | hypothetical protein GYMLUDRAFT_721648 | *G. luxurians* FD-317 M1 | 6E-161 | 59.34 |
| LE_001431 | 146 | 2 | -6.25 | down |  |  |  |  |
| LE_001500 | 352 | 4 | -6.27 | down | cytochrome P450 | *F. hepatica* ATCC 64428 | 0 | 59.57 |
| LE_008136 | 1,524 | 19 | -6.27 | down |  |  |  |  |
| LE_000636 | 1,694 | 22 | -6.27 | down | hypothetical protein PUNSTDRAFT_115895 | *P. strigosozonata* HHB-11173 SS5 | 4E-46 | 50.00 |
| LE_000316 | 10,727 | 137 | -6.28 | down | carbohydrate-binding module family 1 protein | *G. luxurians* FD-317 M1 | 5E-172 | 83.05 |
| LE_008143 | 849 | 10 | -6.30 | down | hypothetical protein GALMADRAFT_229731 | *G. marginata* CBS 339.88 | 9E-130 | 46.53 |
| LE_000465 | 5,565 | 70 | -6.31 | down | hypothetical protein GYMLUDRAFT_52628 | *G. luxurians* FD-317 M1 | 0 | 85.08 |
| LE_008528 | 865 | 10 | -6.34 | down | hypothetical protein GYMLUDRAFT_53143 | *G. luxurians* FD-317 M1 | 0 | 76.17 |
| LE_000301 | 2,236 | 27 | -6.34 | down | hypothetical protein GYMLUDRAFT_97031 | *G. luxurians* FD-317 M1 | 4E-19 | 42.08 |
| LE_001695 | 1,090 | 12 | -6.38 | down | hypothetical protein GYMLUDRAFT_50355 | *G. luxurians* FD-317 M1 | 4E-62 | 52.80 |
| LE_000473 | 7,033 | 84 | -6.38 | down | manganese peroxidase | *L. edodes* | 0 | 100.00 |
| LE_000667 | 10,331 | 124 | -6.38 | down | phosphatidylserine decarboxylase 1 | *Grifola frondosa* | 2E-160 | 56.91 |
| LE_001416 | 984 | 11 | -6.39 | down | AF411252_1 xylanase | *L. edodes* | 1E-169 | 99.29 |
| LE_000920 | 1,039 | 12 | -6.39 | down | alpha-keto glutarate-dependent sulfonate dioxygenase | *Termitomyces sp.* J132 | 0 | 80.33 |
| LE_008318 | 411 | 4 | -6.39 | down | hypothetical protein GYMLUDRAFT_171901 | *G. luxurians* FD-317 M1 | 9E-88 | 66.84 |
| LE_000957 | 1,652 | 19 | -6.44 | down | hypothetical protein GYMLUDRAFT_90895 | *G. luxurians* FD-317 M1 | 0 | 84.42 |
| LE_002907 | 7,598 | 87 | -6.45 | down |  |  |  |  |
| LE_003226 | 355 | 3 | -6.45 | down |  |  |  |  |
| LE_004017 | 149 | 1 | -6.47 | down | hypothetical protein STEHIDRAFT_140822 | *S. hirsutum* FP-91666 SS1 | 1E-42 | 39.70 |
| LE_008690 | 1,117 | 12 | -6.48 | down | alpha/beta-hydrolase | *D. squalens* LYAD-421 SS1 | 6E-51 | 30.63 |
| LE_001981 | 310 | 3 | -6.50 | down | hypothetical protein GYMLUDRAFT_59139 | *G. luxurians* FD-317 M1 | 6E-91 | 57.94 |
| LE_000597 | 13,386 | 146 | -6.52 | down | hypothetical protein GYMLUDRAFT_410740 | *G. luxurians* FD-317 M1 | 0 | 82.03 |
| LE_003185 | 590 | 6 | -6.53 | down | hypothetical protein GYMLUDRAFT_40781 | *G. luxurians* FD-317 M1 | 0 | 75.58 |
| LE_008165 | 1,105 | 11 | -6.55 | down | GMC oxidoreductase | *C. torrendii* FP15055 ss-10 | 0 | 59.20 |
| LE_010197 | 130 | 1 | -6.56 | down | alpha beta-hydrolase | *C. puteana* RWD-64-598 SS2 | 2E-75 | 37.01 |
| LE_000922 | 452 | 4 | -6.59 | down | peptidyl-prolyl cis-trans isomerase | *Termitomyces sp.* J132 | 3E-84 | 74.85 |
| LE_008452 | 440 | 4 | -6.62 | down | hypothetical protein GYMLUDRAFT_232637 | *G. luxurians* FD-317 M1 | 4E-37 | 80.73 |
| LE_008442 | 256 | 2 | -6.63 | down | hypothetical protein GYMLUDRAFT_38034 | *G. luxurians* FD-317 M1 | 1E-112 | 55.52 |
| LE_000074 | 8,560 | 84 | -6.66 | down |  |  |  |  |
| LE_008273 | 835 | 8 | -6.67 | down | carbohydrate-binding module family 50 protein | *G. luxurians* FD-317 M1 | 2E-61 | 82.84 |
| LE_000144 | 2,915 | 28 | -6.67 | down | glycoside hydrolase family 5 protein | *G. luxurians* FD-317 M1 | 0 | 89.12 |
| LE_001490 | 276 | 2 | -6.69 | down |  |  |  |  |
| LE_003413 | 152 | 1 | -6.72 | down | hypothetical protein PLICRDRAFT_39708 | *P. crispa* FD-325 SS-3 | 6E-43 | 36.70 |
| LE_001885 | 664 | 6 | -6.73 | down | hypothetical protein GYMLUDRAFT_36189 | *G. luxurians* FD-317 M1 | 0 | 79.69 |
| LE_003227 | 658 | 6 | -6.77 | down |  |  |  |  |
| LE_004681 | 1,089 | 9 | -6.81 | down | hypothetical protein GYMLUDRAFT_46670 | *G. luxurians* FD-317 M1 | 0 | 61.26 |
| LE_008201 | 2,450 | 22 | -6.81 | down | hypothetical protein GYMLUDRAFT_41664 | *G. luxurians* FD-317 M1 | 0 | 66.17 |
| LE_000135 | 2,515 | 21 | -6.86 | down | TauD-domain-containing protein | *G. trabeum* ATCC 11539 | 0 | 75.12 |
| LE_008335 | 407 | 3 | -6.86 | down | hypothetical protein PLEOSDRAFT_1091014 | *P. ostreatus* PC15 | 1E-76 | 53.41 |
| LE_009181 | 2,508 | 21 | -6.88 | down | carbohydrate esterase family 16 protein | *G. luxurians* FD-317 M1 | 6E-156 | 69.82 |
| LE_008360 | 611 | 5 | -6.90 | down | hypothetical protein GYMLUDRAFT_64913 | *G. luxurians* FD-317 M1 | 7E-119 | 80.86 |
| LE_001064 | 3,307 | 27 | -6.92 | down | glycoside hydrolase family 10 protein | *G. luxurians* FD-317 M1 | 0 | 75.60 |
| LE_001166 | 689 | 5 | -6.94 | down |  |  |  |  |
| LE_000305 | 11,319 | 90 | -6.96 | down | oxaloacetate acetylhydrolase | *C. torrendii FP15055 ss-10* | 0 | 88.25 |
| LE_000202 | 1,593 | 12 | -6.97 | down | hypothetical protein GYMLUDRAFT_34551 | *G. luxurians* FD-317 M1 | 8E-128 | 57.33 |
| LE_008992 | 264 | 2 | -6.97 | down | acetyl-CoA synthetase-like protein | *S. hirsutum FP-91666 SS1* | 0 | 44.54 |
| LE_002012 | 491 | 4 | -6.98 | down | hypothetical protein GYMLUDRAFT_34587 | *G. luxurians* FD-317 M1 | 6E-116 | 89.42 |
| LE_001173 | 467 | 3 | -6.98 | down | glycoside hydrolase family 53 protein | *G. luxurians* FD-317 M1 | 0 | 84.89 |
| LE_005945 | 1,064 | 8 | -6.99 | down | carbohydrate esterase family 4 protein | *G. luxurians* FD-317 M1 | 0 | 78.82 |
| LE_009158 | 2,450 | 19 | -7.00 | down | pyranose 2-oxidase |  | 0 | 57.78 |
| LE_003107 | 660 | 4 | -7.01 | down | glycoside hydrolase family 28 protein | *G. luxurians* FD-317 M1 | 0 | 84.80 |
| LE_008367 | 422 | 3 | -7.01 | down | AF411252_1 xylanase | *L. edodes* | 7E-102 | 81.32 |
| LE_008144 | 4,171 | 32 | -7.02 | down | hypothetical protein GYMLUDRAFT_826941 | *G. luxurians* FD-317 M1 | 1E-82 | 62.11 |
| LE_008089 | 1,480 | 11 | -7.03 | down | hypothetical protein GYMLUDRAFT_74859 | *G. luxurians* FD-317 M1 | 2E-35 | 65.31 |
| LE_001467 | 725 | 5 | -7.09 | down | RTA-like protein | *R. toruloides* NP11 | 8E-87 | 52.57 |
| LE_008145 | 8,753 | 63 | -7.11 | down | hypothetical protein GYMLUDRAFT_889262 | *G. luxurians* FD-317 M1 | 1E-143 | 54.03 |
| LE_001544 | 8,305 | 60 | -7.11 | down | polysaccharide lyase family 1 protein | *G. luxurians* FD-317 M1 | 5E-148 | 87.92 |
| LE_008215 | 2,230 | 15 | -7.17 | down | glycoside hydrolase family 28 protein | *G. luxurians* FD-317 M1 | 0 | 90.25 |
| LE_009146 | 3,921 | 27 | -7.18 | down | hypothetical protein GYMLUDRAFT_62973 | *G. luxurians* FD-317 M1 | 2E-85 | 73.37 |
| LE_002570 | 695 | 4 | -7.19 | down | hypothetical protein GYMLUDRAFT_39627 | *G. luxurians* FD-317 M1 | 1E-123 | 61.54 |
| LE_000238 | 32,716 | 220 | -7.21 | down | hypothetical protein GYMLUDRAFT_47696 | *G. luxurians* FD-317 M1 | 5E-75 | 95.93 |
| LE_008428 | 704 | 5 | -7.21 | down | hypothetical protein AGABI2DRAFT_66714 | *A. bisporus var. bisporus* H97 | 1E-60 | 42.48 |
| LE_008502 | 504 | 3 | -7.24 | down | hypothetical protein GYMLUDRAFT_47995 | *G. luxurians* FD-317 M1 | 3E-59 | 48.11 |
| LE_008253 | 117 | 0 | -7.33 | down | hypothetical protein SCHPADRAFT_942228 | *S. paradoxa* | 3E-58 | 41.56 |
| LE_009133 | 1,223 | 7 | -7.35 | down | carbohydrate-binding module family 1 protein | *G. luxurians* FD-317 M1 | 0 | 86.41 |
| LE_000629 | 573 | 3 | -7.39 | down | hypothetical protein PHACADRAFT_214258 | *Phanerochaete carnosa* HHB-10118-sp | 9E-139 | 61.49 |
| LE_000372 | 3,271 | 19 | -7.40 | down | endo-beta-1,4-glucanase D | *Termitomyces sp.* J132 | 4E-108 | 84.21 |
| LE_010381 | 2,065 | 11 | -7.43 | down | hypothetical protein GYMLUDRAFT_239794 | *G. luxurians* FD-317 M1 | 8E-20 | 56.79 |
| LE_008139 | 465 | 2 | -7.45 | down | hypothetical protein UCREL1_359 | *E. lata* UCREL1 | 0 | 72.05 |
| LE_009444 | 993 | 5 | -7.46 | down | MFS general substrate transporter | *D. squalens* LYAD-421 SS1 | 0 | 76.02 |
| LE_000741 | 2,188 | 12 | -7.47 | down | hypothetical protein GYMLUDRAFT_77937 | *G. luxurians* FD-317 M1 | 0 | 79.35 |
| LE_009147 | 1,208 | 6 | -7.47 | down | hypothetical protein GYMLUDRAFT_37955 | *G. luxurians* FD-317 M1 | 0 | 86.96 |
| LE_010137 | 603 | 3 | -7.49 | down |  |  |  |  |
| LE_000217 | 12,727 | 70 | -7.50 | down | glycoside hydrolase family 74 protein | *G. luxurians* FD-317 M1 | 0 | 80.72 |
| LE_006378 | 278 | 1 | -7.54 | down | hypothetical protein M413DRAFT_261550 | *H. cylindrosporum* h7 | 1E-60 | 58.24 |
| LE_002813 | 157 | 0 | -7.56 | down | hypothetical protein GYMLUDRAFT_926557 | *G. luxurians* FD-317 M1 | 2E-36 | 32.24 |
| LE_008333 | 707 | 3 | -7.56 | down | hypothetical protein MPER_10011 | *M. perniciosa* FA553 | 9E-44 | 35.84 |
| LE_008873 | 203 | 1 | -7.57 | down | Serine protease inhibitor |  | 1E-44 | 54.42 |
| LE_000453 | 523 | 2 | -7.58 | down | Serine protease inhibitor |  | 1E-96 | 100.00 |
| LE_001469 | 1,336 | 6 | -7.61 | down | hypothetical protein GYMLUDRAFT_46670 | *G. luxurians* FD-317 M1 | 0 | 70.33 |
| LE_008131 | 225 | 1 | -7.64 | down | predicted protein | *Postia placenta* Mad-698-R | 3E-120 | 59.02 |
| LE_009281 | 1,268 | 6 | -7.67 | down | hypothetical protein GYMLUDRAFT_250099 | *G. luxurians* FD-317 M1 | 1E-22 | 77.06 |
| LE_009193 | 1,324 | 6 | -7.71 | down | hypothetical protein GYMLUDRAFT_49080 | *G. luxurians* FD-317 M1 | 1E-114 | 61.43 |
| LE_008108 | 333 | 1 | -7.71 | down | cytochrome P450 monooxygenase 78 | *H. irregulare* TC 32-1 | 4E-170 | 53.14 |
| LE_001746 | 135 | 0 | -7.71 | down | similar to An01g14980 | *Aspergillus kawachii* IFO 4308 | 2E-53 | 37.74 |
| LE_001120 | 775 | 3 | -7.77 | down | 2,4-dienoyl-CoA reductase | *C. cinerea* okayama7#130 | 3E-161 | 74.74 |
| LE_009344 | 767 | 3 | -7.78 | down |  |  |  |  |
| LE_000439 | 8,560 | 38 | -7.79 | down | laccase *lcc6* | *L. edodes* | 0 | 99.62 |
| LE_009481 | 189 | 0 | -7.83 | down | hypothetical protein PLEOSDRAFT_1106377 | *P. ostreatus* PC15 | 2E-35 | 35.36 |
| LE_000425 | 4,035 | 17 | -7.85 | down | glycoside hydrolase family 5 protein | *G. luxurians* FD-317 M1 | 0 | 83.71 |
| LE_009048 | 3,006 | 12 | -7.86 | down | hypothetical protein GYMLUDRAFT_77556 | *G. luxurians* FD-317 M1 | 2E-154 | 79.04 |
| LE_009090 | 151 | 0 | -7.87 | down | NAD P-binding protein | *G. trabeum* ATCC 11539 | 7E-72 | 45.34 |
| LE_000243 | 39,293 | 168 | -7.87 | down | hypothetical protein GYMLUDRAFT_244595 | *G. luxurians* FD-317 M1 | 2E-33 | 56.56 |
| LE_005103 | 2,189 | 9 | -7.92 | down | hypothetical protein CYLTODRAFT_436349 | *C. torrendii* FP15055 ss-10 | 5E-69 | 42.21 |
| LE_000405 | 4,095 | 16 | -7.94 | down | hypothetical protein GYMLUDRAFT_35584 | *G. luxurians* FD-317 M1 | 9E-118 | 54.31 |
| LE_000513 | 1,003 | 3 | -7.98 | down | Mfs1.1 | *G. trabeum* ATCC 11539 | 0 | 55.45 |
| LE_008260 | 1,798 | 7 | -8.00 | down | carbohydrate-binding module family 1 protein | *G. luxurians* FD-317 M1 | 4E-163 | 82.52 |
| LE_000619 | 178 | 0 | -8.11 | down |  |  |  |  |
| LE_001223 | 756 | 2 | -8.14 | down | hypothetical protein PLEOSDRAFT_1108863 | *P. ostreatus* PC15 | 5E-41 | 42.49 |
| LE_000585 | 14,016 | 49 | -8.16 | down | hypothetical protein GYMLUDRAFT_42425 | *G. luxurians* FD-317 M1 | 0 | 79.05 |
| LE_004837 | 889 | 3 | -8.16 | down | polysaccharide lyase family 7 protein | *G. luxurians* FD-317 M1 | 8E-140 | 78.08 |
| LE_004044 | 296 | 1 | -8.17 | down |  |  |  |  |
| LE_000704 | 9,661 | 33 | -8.17 | down | glycoside hydrolase family 3 protein | *G. luxurians* FD-317 M1 | 0 | 81.62 |
| LE_002593 | 311 | 1 | -8.28 | down | NAD-binding protein | *F. mediterranea* MF3/22 | 1E-130 | 59.09 |
| LE_001282 | 796 | 2 | -8.30 | down | Ribonuclease Le37 | *L. edodes* | 0 | 99.50 |
| LE_008200 | 1,336 | 4 | -8.30 | down | hypothetical protein JAAARDRAFT_40237 | *Jaapia argillacea* MUCL 33604 | 1E-38 | 32.46 |
| LE_000457 | 865 | 2 | -8.32 | down | hydrophobin | *G. subvermispora* B | 7E-24 | 69.41 |
| LE_000310 | 2,667 | 8 | -8.33 | down | hypothetical protein GALMADRAFT_158926 | *G. marginata* CBS 339.88 | 3E-34 | 34.69 |
| LE_003951 | 7,428 | 22 | -8.38 | down |  |  |  |  |
| LE_002917 | 2,303 | 6 | -8.42 | down |  |  |  |  |
| LE_000300 | 931 | 2 | -8.44 | down | UbiA prenyltransferase | *C. torrendii* FP15055 ss-10 | 5E-97 | 50.80 |
| LE_009276 | 3,222 | 9 | -8.44 | down | glutamic protease | *Pholiota nameko* | 7E-73 | 63.86 |
| LE_009520 | 226 | 0 | -8.45 | down |  |  |  |  |
| LE_002843 | 28,160 | 79 | -8.48 | down | hypothetical protein GYMLUDRAFT_235247 | *G. luxurians* FD-317 M1 | 0 | 81.35 |
| LE_000766 | 2,947 | 7 | -8.51 | down | polysaccharide lyase family 7 protein | *G. luxurians* FD-317 M1 | 3E-102 | 65.46 |
| LE_009308 | 307 | 0 | -8.53 | down | hypothetical protein GYMLUDRAFT_67293 | *G. luxurians* FD-317 M1 | 8E-68 | 64.20 |
| LE_001347 | 2,096 | 5 | -8.54 | down | hypothetical protein GYMLUDRAFT_175090 | *G. luxurians* FD-317 M1 | 3E-47 | 76.03 |
| LE_008126 | 15,792 | 42 | -8.55 | down | hypothetical protein GYMLUDRAFT_47361 | *G. luxurians* FD-317 M1 | 9E-152 | 62.59 |
| LE_004773 | 245 | 0 | -8.56 | down | hypothetical protein GALMADRAFT_157818 | *G. marginata* CBS 339.88 | 2E-93 | 50.29 |
| LE_000191 | 20,289 | 52 | -8.59 | down | hypothetical protein GYMLUDRAFT_48399 | *G. luxurians* FD-317 M1 | 4E-117 | 90.05 |
| LE_000236 | 13,205 | 34 | -8.60 | down | cellulose and lignin medium expressed protein *hep6* | *H. irregulare TC 32-1* | 3E-48 | 66.46 |
| LE_001259 | 9,120 | 23 | -8.62 | down | AF411251_1 cellulase CEL6B | *L. edodes* | 0 | 99.10 |
| LE_000346 | 10,790 | 26 | -8.68 | down | manganese peroxidase | *L. edodes* | 0 | 99.47 |
| LE_001627 | 276 | 0 | -8.74 | down | hypothetical protein GYMLUDRAFT_38358 | *G. luxurians* FD-317 M1 | 1E-107 | 68.35 |
| LE_000292 | 7,260 | 15 | -8.87 | down | hypothetical protein GYMLUDRAFT_833171 | *G. luxurians* FD-317 M1 | 1E-171 | 75.00 |
| LE_000263 | 1,010 | 2 | -8.90 | down | hypothetical protein MPER_10114 | *M. perniciosa* FA553 | 1E-33 | 39.84 |
| LE_000448 | 22,883 | 47 | -8.90 | down | carbohydrate-binding module family 1 protein | *G. luxurians* FD-317 M1 | 0 | 88.71 |
| LE_001019 | 847 | 1 | -8.99 | down | *ekdA* | *B. cinerea* T4 | 2E-22 | 58.82 |
| LE_000250 | 3,990 | 7 | -9.03 | down | endoglucanase | *L. edodes* | 2E-162 | 100.00 |
| LE_000471 | 2,728 | 4 | -9.07 | down | hypothetical protein PLEOSDRAFT_1107016 | *P. ostreatus* PC15 | 2E-96 | 62.55 |
| LE_008107 | 4,728 | 7 | -9.22 | down | aspergillopepsin | *D. squalens* LYAD-421 SS1 | 2E-67 | 64.98 |
| LE_000826 | 3,433 | 5 | -9.24 | down | hypothetical protein GYMLUDRAFT_239787 | *G. luxurians* FD-317 M1 | 1E-165 | 54.36 |
| LE_000360 | 7,837 | 10 | -9.55 | down | carbohydrate-binding module family 1 protein | *G. luxurians* FD-317 M1 | 2E-144 | 78.83 |
| LE_001533 | 27,399 | 35 | -9.58 | down | glycoside hydrolase family 28 protein | *G. luxurians* FD-317 M1 | 4E-177 | 79.12 |
| LE_000333 | 12,709 | 16 | -9.61 | down | manganese peroxidase | *L. edodes* | 0 | 92.84 |
| LE_000560 | 8,415 | 10 | -9.70 | down | glycoside hydrolase family 5 protein | *G. luxurians* FD-317 M1 | 0 | 74.61 |
| LE_000201 | 80,627 | 96 | -9.70 | down | hypothetical protein GYMLUDRAFT_76970 | *G. luxurians* FD-317 M1 | 2E-12 | 50.81 |
| LE_000194 | 1,982 | 2 | -9.70 | down | carbohydrate-binding module family 1 protein | *G. luxurians* FD-317 M1 | 2E-81 | 72.40 |
| LE_001219 | 1,573 | 1 | -9.77 | down | glycoside hydrolase family 131 protein | *Piloderma croceum* F 1598 | 4E-116 | 60.51 |
| LE_000146 | 55,176 | 55 | -9.97 | down | hypothetical protein GYMLUDRAFT_237980 | *G. luxurians* FD-317 M1 | 1E-20 | 77.06 |
| LE_008375 | 673 | 0 | -10.02 | down | MFS monocarboxylate transporter | *H. irregulare* TC 32-1 | 1E-137 | 51.65 |
| LE_002769 | 1,801 | 1 | -10.10 | down | hypothetical protein GYMLUDRAFT_574980 | *G. luxurians* FD-317 M1 | 5E-133 | 63.59 |
| LE_003048 | 8,444 | 6 | -10.31 | down | glycoside hydrolase family 61 protein | *G. luxurians* FD-317 M1 | 2E-102 | 82.98 |
| LE_001168 | 7,376 | 5 | -10.33 | down | hypothetical protein GYMLUDRAFT_36630 | *G. luxurians* FD-317 M1 | 4E-51 | 65.87 |
| LE_000136 | 69,611 | 46 | -10.54 | down |  |  |  |  |
| LE_002915 | 10,348 | 4 | -11.04 | down |  |  |  |  |
| LE_000212 | 23,824 | 11 | -11.08 | down | hypothetical protein GYMLUDRAFT_698361 | *G. luxurians* FD-317 M1 | 4E-112 | 82.27 |
| LE_000758 | 28,499 | 11 | -11.30 | down | tetrapyrrole methylase | *F. mediterranea* MF3/22 | 7E-97 | 46.24 |

^a^*Lentinula edodes, L. edodes; Auricularia subglabra, A. subglabra;* *Agaricus bisporus, A. bisporu; Amanita muscaria, A. muscaria; Beauveria bassiana, B. bassiana; Botrytis cinerea, B. cinerea; Coniophora puteana, C. puteana; Coprinopsis cinerea, C. cinerea; Cylindrobasidium torrendii, C. torrendii; Dichomitus squalens, D. squalens; Fistulina hepatica, F. hepatica; Fomitiporia mediterranea, F. mediterranea; Gloeophyllum trabeum, G. trabeum; Galerina marginata, G. marginata; Gelatoporia subvermispora, G. subvermispora; Glarea lozoyensis, G. lozoyensis; Grifola frondosa, G. frondosa; Gymnopus luxurians, G. luxurians; Hebeloma cylindrosporum, H. cylindrosporum; Heterobasidion irregular, H. irregular; Hypholoma sublateritium, H. sublateritium; Laccaria amethystine, L. amethystine; Laccaria bicolor, L. bicolor; Lentinula edodes, L. edodes; Leptosphaeria maculans, L. maculans; Macrophomina phaseolina, M. phaseolina; Marssonina brunnea, M. brunnea; Moniliophthora perniciosa, M. perniciosa; Neofusicoccum parvum, N. parvum; Paxillus involutus, P.involutus; Penicillium rubens, P. rubens; Phanerochaete chrysosporium, P. chrysosporium; Phlebiopsis gigantean, P. gigantea; Pholiota nameko, P. nameko; Piloderma croceum, P. croceum; Pleurotus ostreatus, P. ostreatus; Plicaturopsis crispa, P. crispa; Postia placenta, P. placenta; Punctularia strigosozonata, P. strigosozonata; Rhizoctonia solani, R. solani; Rhodosporidium toruloides, R. toruloides; Schizophyllum commune, S. commune; Schizopora paradoxa, S. paradoxa; Serpula lacrymans, S. lacrymans; Sphaerobolus stellatus, S. stellatus; Stereum hirsutum, S. hirsutum; Trametes cinnabarina, T. cinnabarina; Trametes versicolor, T. versicolor; Tricholoma vaccinum, T. vaccinum; Trichophyton rubrum, T. rubrum; Zea mays, Z. mays.*Table 9. Functional annotation of specific DEGs in the mycelium

| Unigene ID | Mycelium read count | Fruiting body read count | Log2fold change | Description | Species | Evalue | Identity |
| --- | --- | --- | --- | --- | --- | --- | --- |
| LE_000758 | 28,499 | 11 | -11.30 | tetrapyrrole methylase | *F. mediterranea* MF3/22 | 7E-97 | 46.24 |
| LE_000212 | 23,824 | 11 | -11.08 | Pc16g11320 protein (Precursor) | *P. chrysogenum* | 1E-41 | 45.54 |
| LE_002915 | 10,348 | 4 | -11.04 |  |  |  |  |
| LE_000136 | 69,611 | 46 | -10.54 | translation initiation factor | *D. hafniense* Y51 | 1E-72 | 88.00 |
| LE_001168 | 7,376 | 5 | -10.33 | hydrophobic surface binding protein | *L. bicolor* | 1E-26 | 47.62 |
| LE_003048 | 8,444 | 6 | -10.31 | glycoside hydrolase family 61 protein | *G. luxurians* FD-317 M1 | 2E-102 | 82.98 |
| LE_002769 | 1,801 | 1 | -10.10 | hypothetical protein GYMLUDRAFT_574980 | *G. luxurians* FD-317 M1 | 5E-133 | 63.59 |
| LE_008375 | 673 | 0 | -10.02 | MFS monocarboxylate transporter | *H. irregulare* TC 32-1 | 1E-137 | 51.65 |
| LE_001219 | 1,573 | 1 | -9.77 | glycoside hydrolase family 131 protein | *P. croceum* F 1598 | 4E-116 | 60.51 |
| LE_000194 | 1,982 | 2 | -9.70 | carbohydrate-binding module family 1 protein | *G. luxurians* FD-317 M1 | 2E-81 | 72.40 |
| LE_000560 | 8,415 | 10 | -9.70 | glycoside hydrolase family 5 protein | *G. luxurians* FD-317 M1 | 0 | 74.61 |
| LE_000333 | 12,709 | 16 | -9.61 | manganese peroxidase, partial | *L. edodes* | 0 | 92.84 |
| LE_001533 | 27,399 | 35 | -9.58 | glycoside hydrolase family 28 protein | *G. luxurians* FD-317 M1 | 4E-177 | 79.12 |
| LE_000360 | 7,837 | 10 | -9.55 | carbohydrate-binding module family 1 protein | *G. luxurians* FD-317 M1 | 2E-144 | 78.83 |
| LE_000826 | 3,433 | 5 | -9.24 | cytochrome P450 | *C. cinerea* | 2E-117 | 43.71 |
| LE_008107 | 4,728 | 7 | -9.22 | aspergillopepsin | *D. squalens* LYAD-421 SS1 | 2E-67 | 64.98 |
| LE_000471 | 2,728 | 4 | -9.07 | hypothetical protein PLEOSDRAFT_1107016 | *P. ostreatus* PC15 | 2E-96 | 62.55 |
| LE_000250 | 3,990 | 7 | -9.03 | endoglucanase | *L. edodes* | 2E-162 | 100.00 |
| LE_001019 | 847 | 1 | -8.99 | *ekdA* | *B. cinerea* T4 | 2E-22 | 58.82 |
| LE_000263 | 1,010 | 2 | -8.90 | hypothetical protein MPER_10114 | *M. perniciosa* FA553 | 1E-33 | 39.84 |
| LE_000448 | 22,883 | 47 | -8.90 | carbohydrate-binding module family 1 protein | *G. luxurians* FD-317 M1 | 0 | 88.71 |
| LE_000292 | 7,260 | 15 | -8.87 | hypothetical protein GYMLUDRAFT_833171 | *G. luxurians* FD-317 M1 | 1E-171 | 75.00 |
| LE_000346 | 10,790 | 26 | -8.68 | manganese peroxidase | *L. edodes* | 0 | 99.47 |
| LE_001259 | 9,120 | 23 | -8.62 | cellulase *CEL6B* | *L. edodes* | 0 | 99.10 |
| LE_000236 | 13,205 | 34 | -8.60 | cellulose and lignin medium expressed protein | *H. irregulare* TC 32-1 | 3E-48 | 66.46 |
| LE_008126 | 15,792 | 42 | -8.55 | lectin | *P. ostreatus* | 2E-38 | 34.30 |
| LE_001347 | 2,096 | 5 | -8.54 | hypothetical protein GYMLUDRAFT_175090 | *G. luxurians* FD-317 M1 | 3E-47 | 76.03 |
| LE_000766 | 2,947 | 7 | -8.51 | polysaccharide lyase family 7 protein | *G. luxurians* FD-317 M1 | 3E-102 | 65.46 |
| LE_000300 | 931 | 2 | -8.44 | UbiA prenyltransferase | *C. torrendii* FP15055 ss-10 | 5E-97 | 50.80 |
| LE_009276 | 3,222 | 9 | -8.44 | glutamic protease | *P. nameko* | 7E-73 | 63.86 |
| LE_002917 | 2,303 | 6 | -8.42 |  |  |  |  |
| LE_003951 | 7,428 | 22 | -8.38 |  |  |  |  |
| LE_000310 | 2,667 | 8 | -8.33 | hypothetical protein GALMADRAFT_158926 | *G. marginata* CBS 339.88 | 3E-34 | 34.69 |
| LE_000457 | 865 | 2 | -8.32 | hydrophobin | *G. subvermispora* | 7E-24 | 69.41 |
| LE_001282 | 796 | 2 | -8.30 | ribonuclease *Le37* | *L. edodes* | 0 | 99.50 |
| LE_008200 | 1,336 | 4 | -8.30 | hypothetical protein JAAARDRAFT_40237 | *J. argillacea* MUCL 33604 | 1E-38 | 32.46 |
| LE_000704 | 9,661 | 33 | -8.17 | glycoside hydrolase family 3 protein | *G. luxurians* FD-317 M1 | 0 | 81.62 |
| LE_004837 | 889 | 3 | -8.16 | polysaccharide lyase family 7 protein | *G. luxurians* FD-317 M1 | 8E-140 | 78.08 |
| LE_000585 | 14,016 | 49 | -8.16 | tripeptidyl-peptidase | *A. otae* ATCC MYA-4605 | 0 | 99.00 |
| LE_001223 | 756 | 2 | -8.14 | hypothetical protein PLEOSDRAFT_1108863 | *P. ostreatus* PC15 | 5E-41 | 42.49 |
| LE_008260 | 1,798 | 7 | -8.00 | carbohydrate-binding module family 1 protein | *G. luxurians* FD-317 M1 | 4E-163 | 82.52 |
| LE_000513 | 1,003 | 3 | -7.98 | Mfs1.1 | *G. trabeum* ATCC 11539 | 0 | 55.45 |
| LE_000405 | 4,095 | 16 | -7.94 | hypothetical protein GYMLUDRAFT_35584 | *G. luxurians* FD-317 M1 | 9E-118 | 54.31 |
| LE_005103 | 2,189 | 9 | -7.92 | hypothetical protein CYLTODRAFT_436349 | *C. torrendii* FP15055 ss-10 | 5E-69 | 42.21 |
| LE_009048 | 3,006 | 12 | -7.86 | aromatic peroxygenase | *C. radians* | 0 | 99.00 |
| LE_000425 | 4,035 | 17 | -7.85 | glycoside hydrolase family 5 protein | *G. luxurians* FD-317 M1 | 0 | 83.71 |
| LE_000439 | 8,560 | 38 | -7.79 | laccase *lcc6* | *L. edodes* | 0 | 99.62 |
| LE_009344 | 767 | 3 | -7.78 | hydrophobin-3 | *A. bisporus* | 1E-119 | 98.00 |
| LE_001120 | 775 | 3 | -7.77 | 2,4-dienoyl-CoA reductase | *C. cinerea okayama*7#130 | 3E-161 | 74.74 |
| LE_009193 | 1,324 | 6 | -7.71 | pinoresinol reductase | *A. thaliana* | 0 | 99.00 |
| LE_009281 | 1,268 | 6 | -7.67 | hydrophobin-3 | *A. bisporus* GN=abh3 PE=3 SV=1 | 4E-87 | 88.00 |
| LE_001469 | 1,336 | 6 | -7.61 | cytochrome P450 | *R. norvegicus* GN=Cyp3a9 PE=2 SV=2 | 1E-178 | 100.00 |
| LE_000453 | 523 | 2 | -7.58 | serine protease inhibitor |  | 1E-96 | 100.00 |
| LE_008333 | 707 | 3 | -7.56 | hypothetical protein MPER_10011 | *M. perniciosa* FA553 | 9E-44 | 35.84 |
| LE_010137 | 603 | 3 | -7.49 |  |  |  |  |
| LE_009147 | 1,208 | 6 | -7.47 | glyoxal oxidase (Precursor) | *P. chrysosporium* | 0 | 61.12 |
| LE_000741 | 2,188 | 12 | -7.47 | hypothetical protein GYMLUDRAFT_77937 | *G. luxurians* FD-317 M1 | 0 | 79.35 |
| LE_009444 | 993 | 5 | -7.46 | MFS general substrate transporter | *D. squalens* LYAD-421 SS1 | 0 | 76.02 |
| LE_010381 | 2,065 | 11 | -7.43 | hypothetical protein GYMLUDRAFT_239794 | *G. luxurians* FD-317 M1 | 8E-20 | 56.79 |
| LE_000372 | 3,271 | 19 | -7.40 | putative endo-beta-1,4-glucanase | *Termitomyces sp.* J132 | 4E-108 | 84.21 |
| LE_000629 | 573 | 3 | -7.39 | hypothetical protein PHACADRAFT_214258 | *P. carnosa* HHB-10118-sp | 9E-139 | 61.49 |
| LE_009133 | 1,223 | 7 | -7.35 | carbohydrate-binding module family 1 protein | *G. luxurians* FD-317 M1 | 0 | 86.41 |
| LE_008502 | 504 | 3 | -7.24 | hypothetical protein GYMLUDRAFT_47995 | *G. luxurians* FD-317 M1 | 3E-59 | 48.11 |
| LE_008428 | 704 | 5 | -7.21 | hypothetical protein AGABI2DRAFT_66714 | *A. bisporus var. bisporus* H97 | 1E-60 | 42.48 |
| LE_002570 | 695 | 4 | -7.19 | hypothetical protein GYMLUDRAFT_39627 | *G. luxurians* FD-317 M1 | 1E-123 | 61.54 |
| LE_009146 | 3,921 | 27 | -7.18 | hypothetical protein GYMLUDRAFT_62973 | *G. luxurians* FD-317 M1 | 2E-85 | 73.37 |
| LE_008215 | 2,230 | 15 | -7.17 | glycoside hydrolase family 28 protein | *G. luxurians* FD-317 M1 | 0 | 90.25 |
| LE_001467 | 725 | 5 | -7.09 | RTA-like protein | *R. toruloides* NP11 | 8E-87 | 52.57 |
| LE_008089 | 1,480 | 11 | -7.03 | hypothetical protein GYMLUDRAFT_74859 | *G. luxurians* FD-317 M1 | 2E-35 | 65.31 |
| LE_008144 | 4,171 | 32 | -7.02 | hypothetical protein GYMLUDRAFT_826941 | *G. luxurians* FD-317 M1 | 1E-82 | 62.11 |
| LE_003107 | 660 | 4 | -7.01 | glycoside hydrolase family 28 protein | *G. luxurians* FD-317 M1 | 0 | 84.80 |
| LE_009158 | 2,450 | 19 | -7.00 | pyranose 2-oxidase |  | 0 | 57.78 |
| LE_005945 | 1,064 | 8 | -6.99 | carbohydrate esterase family 4 protein | *G. luxurians* FD-317 M1 | 0 | 78.82 |
| LE_000202 | 1,593 | 12 | -6.97 | hypothetical protein GYMLUDRAFT_34551 | *G. luxurians* FD-317 M1 | 8E-128 | 57.33 |
| LE_001166 | 689 | 5 | -6.94 |  |  |  |  |
| LE_001064 | 3,307 | 27 | -6.92 | glycoside hydrolase family 10 protein | *G. luxurians* FD-317 M1 | 0 | 75.60 |
| LE_008360 | 611 | 5 | -6.90 | hypothetical protein GYMLUDRAFT_64913 | *G. luxurians* FD-317 M1 | 7E-119 | 80.86 |
| LE_009181 | 2,508 | 21 | -6.88 | carbohydrate esterase family 16 protein | *G. luxurians* FD-317 M1 | 6E-156 | 69.82 |
| LE_000135 | 2,515 | 21 | -6.86 | tauD-domain-containing protein | *G. trabeum* ATCC 11539 | 0 | 75.12 |
| LE_000468 | 1,089 | 9 | -6.81 | cytochrome P450 | *A. thaliana* GN=CYP72A13 PE=2 SV=1 | 1E-141 | 100.00 |
| LE_008201 | 2,450 | 22 | -6.81 | cyclopentanone 1,2-monooxygenase | *P. brasiliensis* | 3E-168 | 48.22 |
| LE_003227 | 658 | 6 | -6.77 |  |  |  |  |
| LE_001885 | 664 | 6 | -6.73 | inactive dehydrogenase *EasA* | *C. purpurea* GN=easA PE=3 SV=2 | 0 | 100.00 |
| LE_008273 | 835 | 8 | -6.67 | carbohydrate-binding module family 50 protein | *G. luxurians* FD-317 M1 | 2E-61 | 82.84 |
| LE_000144 | 2,915 | 28 | -6.67 | glycoside hydrolase family 5 protein | *G. luxurians* FD-317 M1 | 0 | 89.12 |
| LE_008165 | 1,105 | 11 | -6.55 | GMC oxidoreductase | *C. torrendii* FP15055 ss-10 | 0 | 59.20 |
| LE_003185 | 590 | 6 | -6.53 | hypothetical protein GYMLUDRAFT_40781 | *G. luxurians* FD-317 M1 | 0 | 75.58 |
| LE_008690 | 1,117 | 12 | -6.48 | alpha/beta-hydrolase | *D. squalens* LYAD-421 SS1 | 6E-51 | 30.63 |
| LE_000957 | 1,652 | 19 | -6.44 | riboflavin transporter MCH5 | *S. cerevisiae* ATCC 204508 | 0 | 98.00 |
| LE_001416 | 984 | 11 | -6.39 | xylanase | *L. edodes* | 1E-169 | 99.29 |
| LE_000920 | 1,039 | 12 | -6.39 | alpha-ketoglutarate-dependent sulfonate dioxygenase | *Termitomyces sp.* J132 | 0 | 80.33 |
| LE_001695 | 1,090 | 12 | -6.38 | hypothetical protein GYMLUDRAFT_50355 | *G. luxurians* FD-317 M1 | 4E-62 | 52.8 |
| LE_008528 | 865 | 10 | -6.34 | rhamnogalacturonyl hydrolase *YesR* | *B. subtilis* 168 GN=yesR PE=1 SV=1 | 0 | 97.00 |
| LE_000301 | 2,236 | 27 | -6.34 | hypothetical protein GYMLUDRAFT_97031 | *G. luxurians* FD-317 M1 | 4E-19 | 42.08 |
| LE_008143 | 849 | 10 | -6.30 | Bifunctional solanapyrone synthase | *A. solani* GN=sol5 PE=1 SV=1 | 0 | 100.00 |
| LE_008136 | 1,524 | 19 | -6.27 |  |  |  |  |
| LE_000636 | 1,694 | 22 | -6.27 | hypothetical protein PUNSTDRAFT_115895 | *P. strigosozonata* HHB-11173 SS5 | 4E-46 | 50.00 |
| LE_001472 | 510 | 6 | -6.23 |  |  |  |  |
| LE_008557 | 1,873 | 25 | -6.23 | hypothetical protein GYMLUDRAFT_1012811 | *G. luxurians* FD-317 M1 | 0 | 77.98 |
| LE_002447 | 998 | 13 | -6.20 | aldo/keto reductase | *F. hepatica* ATCC 64428 | 2E-177 | 74.40 |
| LE_004374 | 2,296 | 32 | -6.14 | acid protease | *S. hirsutum* FP-91666 SS1 | 2E-166 | 62.26 |
| LE_000787 | 1,530 | 22 | -6.13 | hypothetical protein GYMLUDRAFT_36042 | *G. luxurians* FD-317 M1 | 4E-50 | 51.93 |
| LE_000694 | 1,017 | 15 | -6.05 | hypothetical protein GYMLUDRAFT_48227 | *G. luxurians* FD-317 M1 | 2E-86 | 56.73 |
| LE_008193 | 975 | 15 | -6.00 | glycoside hydrolase family 51 protein | *G. luxurians* FD-317 M1 | 0 | 73.11 |
| LE_009873 | 1,754 | 27 | -5.99 | hypothetical protein GYMLUDRAFT_49488 | *G. luxurians* FD-317 M1 | 2E-62 | 60.20 |
| LE_000514 | 620 | 11 | -5.84 | hypothetical protein GYMLUDRAFT_80703 | *G. luxurians* FD-317 M1 | 6E-14 | 35.23 |
| LE_003879 | 630 | 11 | -5.74 | endo-1,6-alpha-mannosidase | *L. bicolor* | 8E-153 | 56.17 |
| LE_001038 | 1,293 | 24 | -5.72 | L30e-like protein | *G. trabeum* ATCC 11539 | 4E-140 | 80.63 |
| LE_001005 | 627 | 12 | -5.70 | hypothetical protein GYMLUDRAFT_73243 | *G. luxurians* FD-317 M1 | 3E-156 | 75.35 |
| LE_008319 | 1,013 | 20 | -5.64 | Cytochrome P450 72A14 | *A. thaliana* GN=CYP72A14 PE=2 SV=1 | 1E-169 | 100.00 |
| LE_008269 | 1,175 | 24 | -5.60 | hypothetical protein GYMLUDRAFT_89650 | *G. luxurians* FD-317 M1 | 3E-134 | 79.38 |
| LE_001452 | 570 | 12 | -5.58 | glycoside hydrolase family 28 protein | *G. luxurians* FD-317 M1 | 2E-171 | 78.02 |
| LE_000430 | 1,894 | 39 | -5.58 | cytochrome P450 | *C. cinerea* | 2E-142 | 45.26 |
| LE_001136 | 609 | 13 | -5.55 | hypothetical protein GYMLUDRAFT_255444 | *G. luxurians* FD-317 M1 | 1E-140 | 66.17 |
| LE_008129 | 1,951 | 42 | -5.54 | hypothetical protein GYMLUDRAFT_833346 | *G. luxurians* FD-317 M1 | 2E-35 | 43.62 |
| LE_000769 | 1,059 | 23 | -5.52 | aldos-2-ulose dehydratase | *P. chrysosporium* | 0 | 48.61 |
| LE_008286 | 1,377 | 31 | -5.47 | hypothetical protein GYMLUDRAFT_145162 | *G. luxurians* FD-317 M1 | 2E-170 | 68.38 |
| LE_002046 | 930 | 21 | -5.41 | hypothetical protein GYMLUDRAFT_72897 | *G. luxurians* FD-317 M1 | 3E-66 | 48.68 |
| LE_009245 | 1,509 | 37 | -5.35 | glycosyltransferase family 1 protein | *G. luxurians* FD-317 M1 | 0 | 52.12 |
| LE_009546 | 1,079 | 27 | -5.31 | prolyl oligopeptidase | *R. toruloides NP11* | 0 | 44.15 |
| LE_000485 | 1,097 | 28 | -5.28 | hypothetical protein GYMLUDRAFT_358211 | *G. luxurians* FD-317 M1 | 1E-48 | 41.81 |
| LE_001209 | 576 | 14 | -5.27 | cytochrome P450 | *A. thaliana* GN=CYP72A14 PE=2 SV=1 | 0 | 99.00 |
| LE_001457 | 1,855 | 49 | -5.23 | NAD P-binding protein | *G. trabeum* ATCC 11539 | 1E-115 | 62.59 |
| LE_008169 | 582 | 15 | -5.21 | hypothetical protein GYMLUDRAFT_49327 | *G. luxurians* FD-317 M1 | 7E-124 | 69.64 |
| LE_001078 | 921 | 25 | -5.17 | hypothetical protein GYMLUDRAFT_43375 | *G. luxurians* FD-317 M1 | 0E+00 | 69.55 |
| LE_000903 | 1,592 | 48 | -5.04 | purine-cytosine permease *fcyB* | *E. nidulans* ATCC 38163 | 0 | 98.00 |
| LE_001476 | 709 | 21 | -5.03 | Cis-aconitate decarboxylase | *M. musculus* GN=Irg1 PE=1 SV=2 | 0 | 99.00 |
| LE_008538 | 527 | 16 | -5.02 | P-loop containing nucleoside triphosphate hydrolase protein | *P. strigosozonata* HHB-11173 SS5 | 1E-113 | 65.71 |
| LE_002364 | 502 | 15 | -5.01 | amidase signature enzyme | *F. mediterranea* MF3/22 | 0 | 78.05 |
| LE_004791 | 721 | 22 | -4.98 | clavaminate synthase-like protein | *F. hepatica* ATCC 64428 | 1E-171 | 75.00 |
| LE_000341 | 1,107 | 35 | -4.98 |  |  |  |  |
| LE_004528 | 1,094 | 35 | -4.95 |  |  |  |  |
| LE_009246 | 1,308 | 42 | -4.94 | hypothetical protein GYMLUDRAFT_40146 | *G. luxurians* FD-317 M1 | 7E-115 | 59.29 |
| LE_001489 | 1,058 | 34 | -4.92 | hypothetical protein GYMLUDRAFT_66353 | *G. luxurians* FD-317 M1 | 3E-163 | 57.17 |
| LE_001028 | 1,143 | 38 | -4.92 | glycoside hydrolase family 16 protein | *G. luxurians* FD-317 M1 | 8E-178 | 81.94 |
| LE_001111 | 810 | 27 | -4.90 | fatty acid conjugase | *C. cinerea okayama*7#130 | 0 | 60.20 |
| LE_004083 | 745 | 25 | -4.89 |  |  |  |  |
| LE_000487 | 1,161 | 39 | -4.87 | dimethylaniline monooxygenase | *M. musculus* GN=Fmo4 PE=2 SV=3 | 0 | 99.00 |
| LE_000911 | 724 | 24 | -4.86 | laccase | *L. edodes* | 0 | 99.81 |
| LE_002673 | 862 | 30 | -4.82 |  |  |  |  |
| LE_000192 | 1,014 | 36 | -4.78 | MAP kinase | *L. edodes* | 0 | 81.20 |
| LE_007893 | 779 | 28 | -4.76 | clavaminate synthase-like protein | *C. torrendii* FP15055 ss-10 | 0 | 71.27 |
| LE_003308 | 1,190 | 45 | -4.72 | cytochrome b2, mitochondrial | *H. anomala GN=CYB2* PE=1 SV=2 | 0 | 100.00 |
| LE_002923 | 607 | 23 | -4.69 | neutral ceramidase | *A. thaliana* GN=At2g38010 PE=3 SV=1 | 0 | 99.00 |
| LE_004050 | 884 | 34 | -4.69 | hypothetical protein GYMLUDRAFT_228507 | *G. luxurians* FD-317 M1 | 0 | 68.11 |
| LE_000147 | 1,201 | 46 | -4.68 | MAP kinase | *L. edodes* | 0 | 72.83 |
| LE_008206 | 689 | 27 | -4.66 |  |  |  |  |
| LE_002030 | 684 | 27 | -4.64 | DUF1793-domain-containing protein | *P. strigosozonata* HHB-11173 SS5 | 0 | 71.09 |
| LE_008366 | 692 | 27 | -4.64 | NAD-P-binding protein | *S. hirsutum* FP-91666 SS1 | 8E-111 | 56.34 |
| LE_009475 | 902 | 36 | -4.63 | O-methylsterigmatocystin oxidoreductase | *A. parasiticus* GN=ordA PE=1 SV=1 | 0 | 99.00 |
| LE_008311 | 1,002 | 40 | -4.62 | acyl-CoA synthetase | *A. bisporus var. bisporus* H97 | 0 | 60.03 |
| LE_001178 | 1,132 | 46 | -4.62 | facilitator superfamily | *L. bicolor* | 9E-159 | 59.55 |
| LE_008351 | 897 | 37 | -4.59 | hypothetical protein GYMLUDRAFT_1020713 | *G. luxurians* FD-317 M1 | 1E-125 | 81.85 |
| LE_009554 | 873 | 40 | -4.45 | alpha/beta-hydrolase | *S. paradoxa* | 0 | 72.02 |
| LE_003942 | 765 | 35 | -4.44 | glutamine amidotransferase DUG3 | *S. cerevisiae* ATCC 204508 | 2E-137 | 50.55 |
| LE_003938 | 703 | 32 | -4.42 | aryl-alcohol dehydrogenase | *S. pombe* ATCC 24843 | 0 | 77.84 |
| LE_000773 | 941 | 45 | -4.38 | hypothetical protein GYMLUDRAFT_55759 | *G. luxurians* FD-317 M1 | 7E-63 | 79.70 |
| LE_000991 | 502 | 24 | -4.37 |  |  |  |  |
| LE_008255 | 865 | 42 | -4.37 | cytochrome P450 | *P. strigosozonata* HHB-11173 SS5 | 0 | 64.47 |
| LE_001482 | 588 | 31 | -4.23 | glycoside hydrolase family 30 protein | *G. luxurians* FD-317 M1 | 0 | 82.86 |
| LE_002024 | 573 | 33 | -4.10 | cytochrome P450 | *T. versicolor* FP-101664 SS1 | 6E-133 | 41.72 |
| LE_001661 | 806 | 47 | -4.10 | isochorismatase domain-containing protein 1 | *S. salar* GN=isoc1 PE=2 SV=1 | 0 | 99.00 |
| LE_009139 | 504 | 30 | -4.07 | glutathione S-transferase | *Termitomyces sp.* J132 | 2E-62 | 49.77 |
| LE_008339 | 552 | 33 | -4.05 | hypothetical protein GYMLUDRAFT_262104 | *G. luxurians* FD-317 M1 | 5E-29 | 57.39 |
| LE_001045 | 788 | 47 | -4.05 | hypothetical protein GYMLUDRAFT_248375 | *G. luxurians* FD-317 M1 | 5E-130 | 60.65 |
| LE_004282 | 546 | 35 | -3.97 | glycoside hydrolase family 1 protein | *G. luxurians* FD-317 M1 | 0 | 84.38 |
| LE_000862 | 519 | 33 | -3.95 | Gibberellin 2-oxidase | *E. dermatitidis* | 3E-123 | 55.92 |
| LE_008481 | 602 | 40 | -3.89 | NAD dependent epimerase/dehydratase | *R. emersonii* CBS 393.64 | 5E-86 | 47.19 |
| LE_008192 | 523 | 36 | -3.85 |  |  |  |  |
| LE_004665 | 528 | 38 | -3.77 | FMN-linked oxidoreductase | *C. torrendii* FP15055 ss-10 | 1E-147 | 57.63 |
| LE_002296 | 518 | 40 | -3.69 | carbohydrate-binding module family 20 protein | *G. luxurians* FD-317 M1 | 0 | 81.57 |
| LE_001108 | 547 | 44 | -3.63 | ammonia transport outward protein | *S cerevisiae* ATCC 204508 | 1E-160 | 98.00 |

Table 10. Genes involved in replication, recombination, repair, and chromosome segregation in *L. edodes*.

| Unigene ID | Mycelium read count | Fruiting body read count | Log2fold change | Description | Species | Evalue | Identity | DB | Subclass/Pathway |
| --- | --- | --- | --- | --- | --- | --- | --- | --- | --- |
| LE_000498 | 54 | 243 | 2.18 | alpha-ketoglutarate-dependent dioxygenase *alkB* | *H. sapiens* | 0 | 99.00 | KOG | replication, recombination and repair |
| LE_001170 | 157 | 380 | 1.27 | glycosyltransferase family 90 protein | *G. luxurians* FD-317 M1 | 0 | 58.72 | KOG | replication, recombination and repair |
|  |  |  |  |  |  |  |  | GO | DNA repair |
| LE_001447 | 131 | 1,950 | 3.90 | histone acetyltransferase | *S. cerevisiae* | 0 | 100.00 | KOG | chromatin structure and dynamics |
| LE_001793 | 34 | 112 | 1.74 | transcription initiation factor IIA gamma subunit | *S. hirsutum* FP-91666 SS1 | 8E-56 | 85.15 | KEGG | basal transcription factors |
| LE_002613 | 130 | 296 | 1.18 | hypothetical protein GYMLUDRAFT_238472 | *G. luxurians* FD-317 M1 | 0 | 59.49 | KOG | replication, recombination and repair |
| LE_003435 | 280 | 748 | 1.42 | DNA repair protein, SNF2 family | *L. bicolor* S238N-H82 | 0 | 83.05 | KEGG | homologous recombination |
|  |  |  |  |  |  |  |  | KOG | replication, recombination and repair |
| LE_003671 | 51 | 536 | 3.38 | poly(A) RNA polymerase *cid14* | *Termitomyces sp.* J132 | 1E-165 | 46.83 | KOG | replication, recombination and repair |
| LE_004118 | 489 | 1,219 | 1.32 | chromosome 1 | *T. hirsuta* strain 072 | 6E-06 | 92.00 | GO | germ cell development |
| LE_004329 | 87 | 296 | 1.76 | transcription elongation regulator | *M. musculus* | 0 | 99.00 | KEGG | spliceosome |
| LE_004351 | 45 | 127 | 1.48 | F-box protein At3g54460 | *A. thaliana* | 0 | 100.00 | KOG | replication, recombination and repair |
| LE_005104 | 61 | 155 | 1.33 | ribonuclease H | *Termitomyces sp.* J132 | 5E-79 | 48.67 | KEGG | DNA replication |
|  |  |  |  |  |  |  |  | KOG | replication, recombination and repair |
| LE_005169 | 94 | 260 | 1.46 | condensin complex subunit SMC2 | *F. mediterranea* MF3/22 | 0 | 57.17 | KOG | chromatin structure and dynamics |
|  |  |  |  |  |  |  |  | GO | chromosome segregation |
| LE005258 | 12 | 119 | 3.22 | MutS protein homolog 5 | *H. sapiens* | 0 | 99.00 | KOG | replication, recombination and repair |
|  |  |  |  |  |  |  |  | GO | DNA repair |
|  |  |  |  |  |  |  |  | GO | chromosome segregation |
|  |  |  |  |  |  |  |  | GO | reciprocal meiotic recombination |
| LE_005383 | 33 | 121 | 1.84 | SNF2 family DNA-dependent ATPase | *L. bicolor* S238N-H82 | 0 | 59.66 | KOG | replication, recombination and repair |
| LE_005674 | 44 | 127 | 1.53 | DNA mismatch repair protein MutL | *G. trabeum* ATCC 11539 | 3E-124 | 55.53 | KEGG | mismatch repair |
|  |  |  |  |  |  |  |  | KOG | replication, recombination and repair |
|  |  |  |  |  |  |  |  | GO | DNA repair |
| LE_005791 | 37 | 167 | 2.18 | SET domain-containing protein 5 | *S. pombe* | 0 | 97.00 | KOG | chromatin structure and dynamics |
| LE_005828 | 117 | 289 | 1.30 | ATP-dependent RNA helicase | *C. tanzawaensis* NRRL Y-17324 | 2E-36 | 76.00 | KEGG | spliceosome |
| LE_005887 | 67 | 199 | 1.55 | kinesin | *C. cinerea okayama*7#130 | 3E-75 | 73.00 | GO | microtubule associated complex |
|  |  |  |  |  |  |  |  | GO | spliceosomal complex |
| LE_006012 | 30 | 124 | 2.03 | DNA ligase/mRNA capping enzyme, partial | *P. strigosozonata* HHB-11173 SS5 | 4E-134 | 42.20 | KEGG | non-homologous end-joining |
|  |  |  |  |  |  |  |  | GO | DNA repair |
| LE_006205 | 85 | 314 | 1.89 | cell division control protein 6 homolog | *H. sapiens* | 0 | 99.00 | KOG | replication, recombination and repair |
|  |  |  |  |  |  |  |  | GO | chromosome segregation |
| LE_006537 | 14 | 114 | 3.00 | DNA repair and recombination protein | *Termitomyces sp.* J132 | 0 | 49.64 | KOG | replication, recombination and repair |
| LE_006608 | 32 | 165 | 2.34 | LeR2153 microsatellite | *L. edodes* | 3E-126 | 97.00 | KOG | replication, recombination and repair |
| LE_006626 | 103 | 617 | 2.58 | cell division control protein 45 homolog | *D. kikkawai* | 2E-03 | 93.00 | KOG | chromatin structure and dynamics |
|  |  |  |  |  |  |  |  | GO | chromosome segregation |
| LE_006749 | 53 | 177 | 1.73 | survival of motor neuron-related-splicing factor 30 | *Termitomyces sp.* J132 | 4E-86 | 68.62 | KEGG | spliceosome |
| LE_006852 | 21 | 127 | 2.54 | origin recognition complex subunit 2 | *Termitomyces sp.* J132 | 1E-143 | 54.63 | KOG | replication, recombination and repair |
| LE_008576 | 32 | 105 | 1.72 | NAD-dependent histone deacetylase | *Termitomyces sp.* J132 | 4E-159 | 52.52 | KOG | chromatin structure and dynamics |
|  |  |  |  |  |  |  |  | GO | DNA repair |
|  |  |  |  |  |  |  |  | GO | chromosome segregation |
| LE_009426 | 42 | 531 | 3.64 | hypothetical protein GYMLUDRAFT_254265 | *G. luxurians* FD-317 M1 | 0 | 66.29 | GO | chromosome segregation |
| LE_009910 | 120 | 588 | 2.29 | Nucleosome | *B. napus* | 8E-02 | 100.00 | KEGG | spliceosome |
|  |  |  |  |  |  |  |  | KOG | replication, recombination and repair |
| LE_010297 | 38 | 299 | 2.95 | P-loop containing nucleoside triphosphate hydrolase | *C. torrendii* FP15055 ss-10 | 4E-88 | 47.56 | KOG | replication, recombination and repair |
| LE_010778 | 39 | 181 | 2.18 | DNA mismatch repair enzyme mRNA | *P. coatneyi* | 6E-03 | 97.00 | KEGG | mismatch repair |
|  |  |  |  |  |  |  |  | KOG | replication, recombination and repair |
|  |  |  |  |  |  |  |  | GO | DNA repair |
| LE_011045 | 50 | 135 | 1.43 | dynactin subunit 2 | *D. discoideum* | 0 | 99.00 | GO | microtubule associated complex |

Table S11. Primers used for the quantitative RT-PCR analysis of *L. edodes* unigenes.

| KEGG subclassification | Transcript ID | Mycelium read count | Fruiting body read count | Log2fold  change | Gene description | Sequence (5΄-3΄) |
| --- | --- | --- | --- | --- | --- | --- |
|  |  |  |  |  | 18S rRNA (Tang, L. H. *et al*., 2013) | F: AAACGGCTACCACATCCA  R: CACCAGACTTGCCCTCCA |
| Cell growth and death | LE_005169 | 94 | 260 | 1.46 | condensin complex subunit SMC2 | F: GTTCGTTCAACCATAGAC  R: CACAGTTCATCGTAGTATC |
|  | LE_000976 | 2,213 | 433 | -2.35 | cohesin subunit *psc3* | F: CTCTTGCTTGCTGTGTAG  R: TGTACTCCGAGGTTGATG |
| Transport and catabolism | LE_005408 | 31 | 202 | 2.69 | carnitine acetyl transferase | F: ATATCTGGATGTGGTTGAG  R: CCTGGATTCTTCTTGTCTAT |
|  | LE_002706 | 46,158 | 3,384 | -3.77 | NADPH oxidase | F: CTAAGGTTCAGTGGTTGT  R: TAATGTGCCGTAGTATGG |
| Membrane transport | LE_009569 | 1,361 | 85 | -3.99 | AtrD, ABC-transporter | F: TCACCTTCTCATCAACATC  R: TATTCCGTCGTGGTCATA |
|  | LE_000772 | 4,637 | 468 | -3.31 | leptomycin B resistance protein *pmd1* | F: TCGGCACTAAGTATGAAC  R: GTATCGTAACCAAGAGGAA |
| Signal transduction | LE_006894 | 86 | 306 | 1.83 | pheromone receptor | F: CTCTTATGATGCTGGTCTAAC  R: CCTTCAATGTCAGTCAGTATC |
|  | LE_009126 | 13,744 | 257 | -5.74 | MAP kinase | F: CCGTGATAATGTTCTCGTTA  R: AGTGGCTATCAAGAAGATTC |
| Folding, sorting and degradation | LE_003671 | 51 | 536 | 3.38 | Poly(A) RNA polymerase cid14 | F: AGAGTAGTTCAGGAGGTT  R: CCATTAGACATCACGAGAG |
|  | LE_000220 | 27,429 | 2,337 | -3.55 | disulfide isomerase | F: TTACCCTTACCCTATCCT  R: CACTAGAGACTTCATCAATG |
| Replication and repair | LE_003435 | 280 | 748 | 1.42 | DNA repair protein, SNF2 family | F: TACAGGCGGAATCCAATA  R: CTAATGCTTCATCACCAGTA |
|  | LE_000713 | 3,915 | 449 | -3.12 | hypothetical protein GYMLUDRAFT_49011 | F: TAGTTCCGTTGATGAGAGT  R: CAGTTGCTTGACAGGTAG |
| Transcription | LE_009910 | 120 | 588 | 2.29 | transcript variant | F: GAAGGAGCGGCAAGTATA  R: CTGTTGGAACATCATCTGAG |
|  | LE_001189 | 4,462 | 1,781 | -1.33 | aminopeptidase 2, mitochondrial | F: TATTCTCTGCGTCACTGAT  R: TCTGTTCCTTCTGCTGAG |
|  |  |  |  |  |  |  |
| Translation | LE_002605 | 615 | 2,963 | 2.27 | translation initiation factor *eif2* gamma subunit | F: AAGGCACGAAGGTATGTAA  R: GGCACGGTATTAGAAGTAGA |
|  | LE_000189 | 27,799 | 599 | -5.53 | nuclear pore glycoprotein p62 | F: CGATTCTGGAAGTCAAGT  R: GAGGTTGCTTACTATCACT |
| Amino acid metabolism | LE_007442 | 12 | 2,826 | 7.83 | O-acetylhomoserine (thiol)-lyase | F: CGGCAAATTAGGGCAAAT  R: ATGATGGATGTAGCGACTA |
|  | LE_000274 | 26,713 | 2,925 | -3.19 | methionine adenosyltransferase | F: CGAGATGATGCGAGTAAT  R: AGAAGACTGCTTGCTATG |
| Biosynthesis of other secondary metabolites | LE_008180 | 604 | 54 | -3.48 | dienelactone hydrolase | F: TCCGAATATCATCACCAACT  R: ATGTTCACTCTGCGACTT |
|  | LE_010317 | 27 | 155 | 2.51 | hypothetical protein GYMLUDRAFT_50564 | F: GCATCAACCTATCAACTG  R: CTCTATTCCGACCTTCAT |
| Carbohydrate metabolism | LE_006358 | 1,860 | 6,142 | 1.72 | Aldehyde dehydrogenase | F: TGATAACGACACCGACTG  R: GTTGGCACAGAAGGATTC |
|  | LE_000560 | 8,415 | 10 | -9.70 | glycoside hydrolase family 5 protein | F: CAATAATCGCCACAACATC  R: TTGACTACACAGGAACGA |
| Energy metabolism | LE_006067 | 358 | 2,889 | 3.01 | GMC oxidoreductase | F: GACCTTCTTCCACTCATG  R: GTTCCACCGTTACTAATCG |
|  | LE_000222 | 13,584 | 3,496 | -1.96 | plasma membrane H^+^-transporting ATPase | F: AATAGCGGAAGGAACCAT  R: CCACCACTAGATTCATAAGC |
| Glycan biosynthesis and metabolism | LE_002628 | 64 | 279 | 2.13 | glycoside hydrolase family 2 protein | F: ATCAGGTGTTGAGTAAGAG  R: GACGAAGAAGAAGAGGAA |
|  | LE_001244 | 811 | 164 | -2.31 | beta-N-acetylhexosaminidase | F: CTCCGTTAAGTGATAGTTC  R: GTTAAGATGACCACCTTC |
| Lipid metabolism | LE_000916 | 2,028 | 8,433 | 2.06 | phosphatidylserine decarboxylase | F: CTTCCAGTTCCATTCAGA  R: GACCAACCTACAATCAAC |
|  | LE_002934 | 2,909 | 217 | -3.75 | neutral/alkaline nonlysosomal ceramidase | F: AAGGAGCGTCAACCATAT  R: GTAACCGTATCCGCAATG |
| Metabolism of cofactors and vitamins | LE_003268 | 32 | 134 | 2.05 | hypothetical protein GYMLUDRAFT_154015 | F: CGGACGATGAATTGATAGTT  R: GGTATGACAGGATGTAGGT |
|  | LE_008168 | 1,347 | 326 | -2.05 | C-1-tetrahydrofolate synthase, cytoplasmic | F: GACACAACAGAACCAGAC  R: GACCAAAGCCTCAACTAC |
| Metabolism of other amino acids | LE_007464 | 24 | 3,310 | 7.10 | gamma-glutamyl transpeptidase | F: CAACGAGCGATACATAAG  R: CTATAATGGCACCTCAATC |
|  | LE_000704 | 9,661 | 33 | -8.17 | glycoside hydrolase family 3 protein | F: GCGTGATACCTTGAGAAT  R: AGCCTCTTATGGAACAAC |
| Metabolism of terpenoids and polyketides | LE_003886 | 134 | 358 | 1.42 | farnesyltranstransferase | F: CAACACTCAGTAGGCATT  R: CGAACTTCTCAACCGTAT |
|  | LE_000141 | 780 | 179 | -2.12 | Diphosphomevalonate decarboxylase | F: GGCATGGAATTGGTTAGAG  R: AGGATGGAGGCGATTATC |
| Nucleotide metabolism | LE_007303 | 35 | 238 | 2.77 | glycoside hydrolase family 18 protein | F: GGTTGTAGAAGAAGGTTGGA  R: CGTTTGCCCGAAATGTAAT |
|  | LE_001451 | 411 | 31 | -3.71 | sulfate adenylyltransferase | F: CGTCAGAGTGTTAAGGATAC  R: CAGTCTCACAGTGTTC |
